# A gigantic new dinosaur from Argentina and the evolution of the sauropod hind foot

**DOI:** 10.1038/srep19165

**Published:** 2016-01-18

**Authors:** Bernardo J. González Riga, Matthew C. Lamanna, Leonardo D. Ortiz David, Jorge O. Calvo, Juan P. Coria

**Affiliations:** 1 Laboratorio de Dinosaurios, Facultad de Ciencias Exactas y Naturales, Universidad Nacional de Cuyo, Avenida Padre Contreras 1300, Edificio ECT, Parque Gene San Martín, (5500) Mendoza, Argentina; 2 Consejo Nacional de Investigaciones Científicas y Técnicas (CONICET), IANIGLA-CCT-Mendoza, Argentina; 3 Section of Vertebrate Paleontology, Carnegie Museum of Natural History, 4400 Forbes Avenue, Pittsburgh, PA, 15213 USA; 4 Centro Paleontológico Lago Barreales, Proyecto Dino, Universidad Nacional del Comahue, Ruta Provincial 51, km. 65, Neuquén, Argentina

## Abstract

Titanosauria is an exceptionally diverse, globally-distributed clade of sauropod dinosaurs that includes the largest known land animals. Knowledge of titanosaurian pedal structure is critical to understanding the stance and locomotion of these enormous herbivores and, by extension, gigantic terrestrial vertebrates as a whole. However, completely preserved pedes are extremely rare among Titanosauria, especially as regards the truly giant members of the group. Here we describe *Notocolossus gonzalezparejasi* gen. et sp. nov. from the Upper Cretaceous of Mendoza Province, Argentina. With a powerfully-constructed humerus 1.76 m in length, *Notocolossus* is one of the largest known dinosaurs. Furthermore, the complete pes of the new taxon exhibits a strikingly compact, homogeneous metatarsus—seemingly adapted for bearing extraordinary weight—and truncated unguals, morphologies that are otherwise unknown in Sauropoda. The pes underwent a near-progressive reduction in the number of phalanges along the line to derived titanosaurs, eventually resulting in the reduced hind foot of these sauropods.

Titanosaurian sauropod dinosaurs were the most diverse and abundant large-bodied terrestrial herbivores in the Southern Hemisphere landmasses during the Cretaceous Period^1^,^2^,^3^,^4^. Their fossils have been discovered on all continents, and titanosaur species comprise approximately one third of known sauropod diversity^1^. Some taxa are regarded as the most massive terrestrial animals known to science^4^,^5^,^6^, whereas others were apparently no heavier than modern cattle^7^. Titanosaurs were particularly diverse during the Late Cretaceous, and encompass a taxonomic richness that rivals that of the hadrosaurid ornithischians that dominated Northern Hemisphere palaeoecosystems at the same time.

Anatomical and phylogenetic analyses of titanosaurs form the foundation for insights into the evolution and palaeobiology of this diverse dinosaur group, but such studies have been hampered by missing data, since the osteology of many titanosaurian species is not well understood. Fortunately, this situation is beginning to change with recent discoveries of well-preserved specimens of taxa such as *Tapuiasaurus* from the Early Cretaceous of Brazil^8^, *Dreadnoughtus*^4^, *Futalognkosaurus*^6^, and *Mendozasaurus*^9^ from the Late Cretaceous of Argentina, and *Rapetosaurus* from the latest Cretaceous of Madagascar^10^. Nevertheless, some areas of the titanosaurian skeleton remain poorly documented, particularly the skull, posterior-most caudal vertebrae, and pes^3^. This problem is especially pronounced in the exceptionally gigantic members of the clade, in which these skeletal regions remain all but unknown. Here we describe a new Late Cretaceous sauropod, *Notocolossus gonzalezparejasi* gen. et sp. nov., that offers important new data on the pedal morphology of giant titanosaurs. Specimens of *Notocolossus* were discovered in southern-most Mendoza Province, Argentina (Fig. 1a) by the senior author (B.J.G.R.), and are housed at the Laboratorio de Dinosaurios of the Universidad Nacional de Cuyo (UNCUYO-LD) in the city of Mendoza, Argentina.

## Results

### Systematic palaeontology

Dinosauria Owen, 1842

Saurischia Seeley, 1887

Sauropoda Marsh, 1878

Titanosauriformes Salgado, Coria, and Calvo, 1997

Somphospondyli Wilson and Sereno, 1998

Titanosauria Bonaparte and Coria, 1993

Lithostrotia Upchurch, Barrett, and Dodson, 2004

*Notocolossus gonzalezparejasi* gen. et sp. nov.

### Etymology

From the Greek *notos* (southern) and the Latin *colossus*, in reference to the gigantic size and Gondwanan provenance of the new taxon. Species name honours Dr. Jorge González Parejas, who has collaborated and provided legal guidance on the research, protection, and preservation of dinosaur fossils from Mendoza Province for nearly two decades. In so doing, he has advised researchers on the creation of a natural park that serves to protect dinosaur footprints in Mendoza.

### Holotype

**Figure 1:**
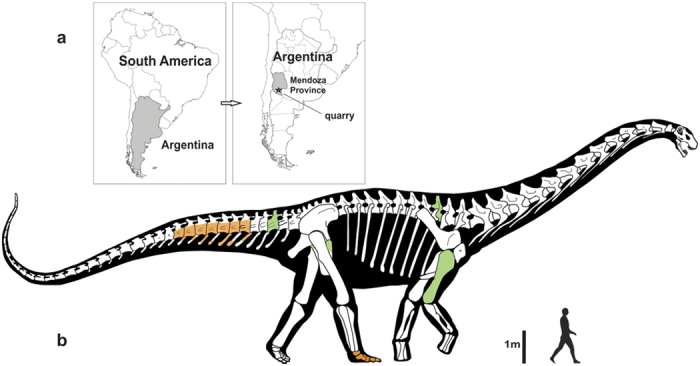
Geographic provenance and speculative reconstruction of the gigantic titanosaurian sauropod dinosaur *Notocolossus gonzalezparejasi* gen. et sp. nov. (**a**) Type locality of *Notocolossus* (indicated by star) in southern-most Mendoza Province, Argentina. (**b**) Reconstructed skeleton and body silhouette in right lateral view, with preserved elements of the holotype (UNCUYO-LD 301) in light green and those of the referred specimen (UNCUYO-LD 302) in orange. Scale bar, 1 m. (All images were hand drawn by the senior author [B.J.G.R.] and subsequently edited using Adobe Illustrator software.)

**Figure 2:**
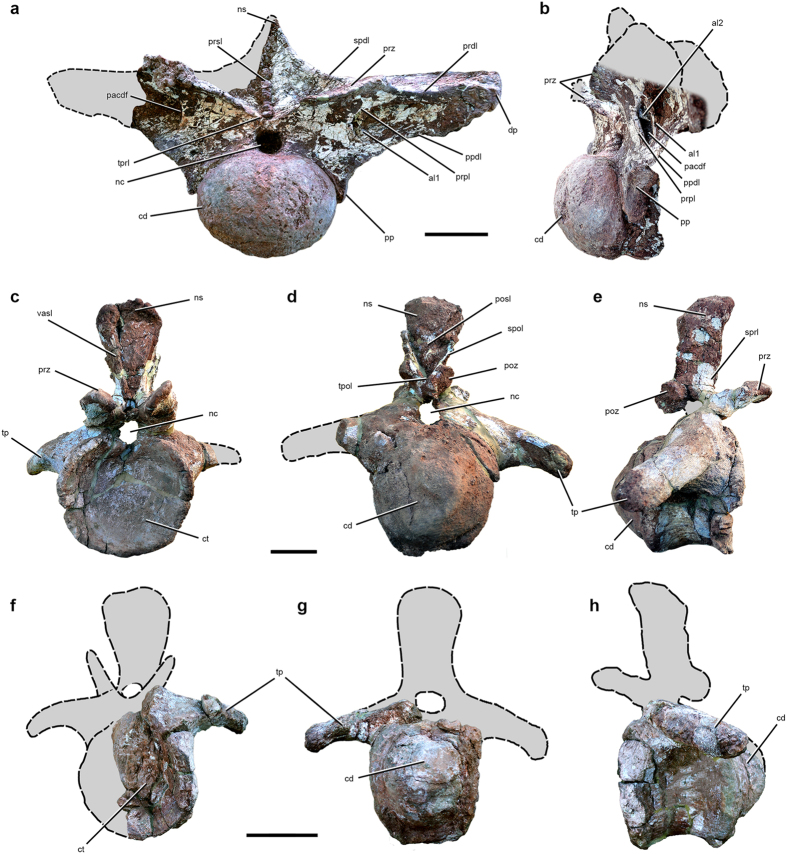
Vertebral morphology of *Notocolossus gonzalezparejasi*. Anterior (second or third) dorsal vertebra of the holotype (UNCUYO-LD 301) in (**a**) anterior and (**b**) left anterolateral views. Anterior caudal vertebra of the holotype (UNCUYO-LD 301) in (**c**) anterior, (**d**) posterior, and (**e**) right lateral views. Anterior caudal vertebra of the referred specimen (UNCUYO-LD 302) in (**f**) anterior, (**g**) posterior, and (**h**) left lateral views. Abbreviations: al1, ‘accessory’ lamina 1; al2, ‘accessory’ lamina 2; cd, condyle; ct, cotyle; dp, diapophysis; nc, neural canal; ns, neural spine; pacdf, parapophyseal centrodiapophyseal fossa; posl, postspinal lamina; poz, postzygapophysis; pp, parapophysis; ppdl, paradiapophyseal lamina; prdl, prezygodiapophyseal lamina; prpl, prezygoparapophyseal lamina; prsl, prespinal lamina; prz, prezygapophysis; spdl, spinodiapophyseal lamina; spol, spinopostzygapophyseal lamina; sprl, spinoprezygapophyseal lamina; tp, transverse process; tpol, intrapostzygapophyseal lamina; tprl, intraprezygapophyseal lamina; vasl, ‘V-shaped’ anterior spinal lamina. Scale bars, 20 cm (**a**,**b**), 10 cm (**c**–**h**).

**Figure 3:**
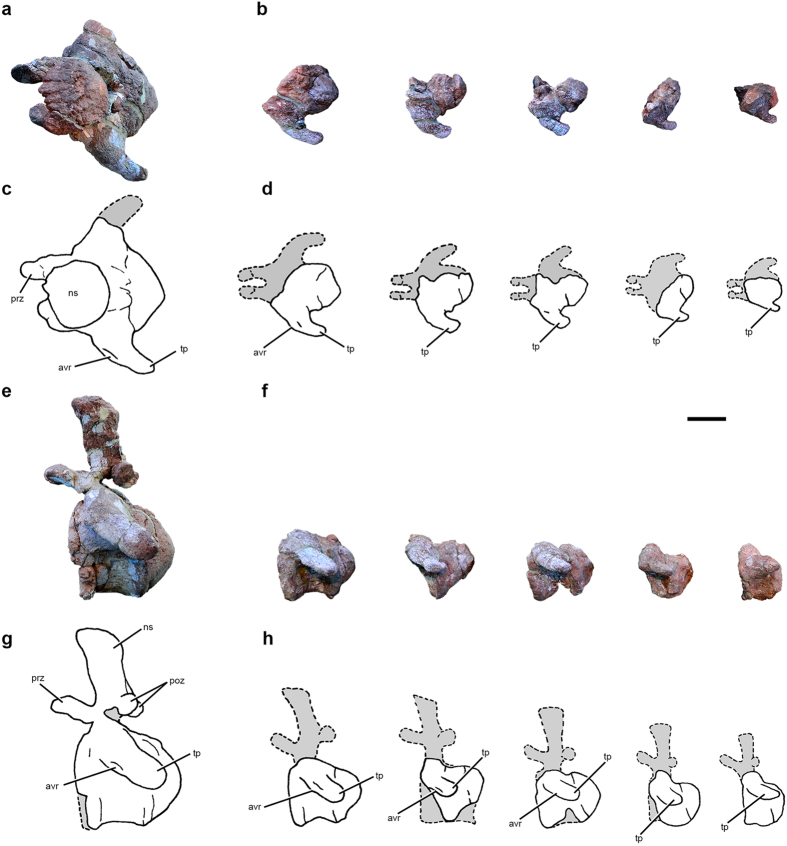
Comparison of anterior caudal vertebrae of *Notocolossus gonzalezparejasi*. Photographs (**a**,**b**,**e**,**f**) and interpretive drawings (**c**,**d**,**g**,**h**) of the anterior caudal vertebra of the holotype (UNCUYO-LD 301) (**a**,**c**,**e**,**g**) and the first five anterior caudal vertebrae of the referred specimen (UNCUYO-LD 302) (**b**,**d**,**f**,**h**) in dorsal (**a**–**d**) and left lateral (**e**–**h**) views (**e** and **g** reversed). Abbreviations, avr, anteroventral ridge; ns, neural spine; poz, postzygapophysis; prz, prezygapophysis; tp, transverse process. Scale bar, 10 cm.

**Figure 4:**
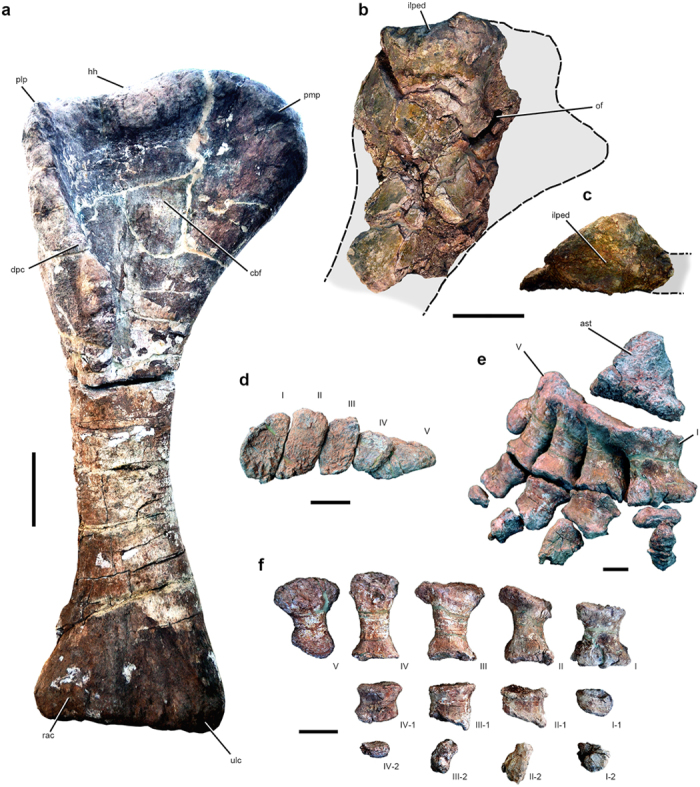
Appendicular skeletal morphology of *Notocolossus gonzalezparejasi*. (**a**) Right humerus of the holotype (UNCUYO-LD 301) in anterior view. Proximal end of the left pubis of the holotype (UNCUYO-LD 301) in lateral (**b**) and proximal (**c**) views. Right tarsus and pes of the referred specimen (UNCUYO-LD 302) in (**d**) proximal (articulated, metatarsus only, dorsal [=anterior] to top), (**e**) dorsomedial (articulated), and (**f**) dorsal (disarticulated) views. Abbreviations: I–V, metatarsal/digit number; 1–2, phalanx number; ast, astragalus; cbf, coracobrachialis fossa; dpc, deltopectoral crest; hh, humeral head; ilped, iliac peduncle; of, obturator foramen; plp, proximolateral process; pmp, proximomedial process; rac, radial condyle; ulc, ulnar condyle. Scale bars, 20 cm (**a**–**c**), 10 cm (**d**–**f)**.

UNCUYO-LD 301, an associated partial skeleton of a very large individual consisting of an anterior dorsal vertebra, an anterior caudal vertebra, the right humerus, and the proximal end of the left pubis (Figs 1b, 2a–e, 3a,c,e,g and 4a–c; Supplementary Figs S1, S3). We consider these elements to represent a single titanosaurian individual because they were found within an area of 8 m by 8 m at the same stratigraphic level and are of the appropriate size and morphology to have been derived from a single skeleton.

### Referred specimen

UNCUYO-LD 302, an associated partial skeleton of a second, smaller-bodied individual that includes an articulated anterior caudal series (consisting of seven partial vertebrae and haemal arches) and the complete and articulated right astragalus and pes (Figs 1b, 2f–h, 3b,d,f,h and 4d–f; Supplementary Figs S2, S4–S8). As with the holotype, we consider these elements to represent a single titanosaurian individual because they were found within an area of 5 m by 5 m at the same stratigraphic level and are of the appropriate size and morphology to have come from a single skeleton. See Supplementary Information for justification of the referral of this specimen to *Notocolossus gonzalezparejasi*.

### Type locality and horizon

Cerro Guillermo, Malargüe Department, southern-most Mendoza Province, Argentina (Fig. 1a; coordinates on file at UNCUYO-LD). The holotype and referred specimen were collected 403 m apart in the basal-most bed of the Upper Cretaceous (upper Coniacian–lower Santonian, ~86 Ma) Plottier Formation of the Neuquén Group (see Supplementary Information for details).

### Diagnosis

Large titanosaurian sauropod dinosaur diagnosed by the following autapomorphies: (1) anterior dorsal vertebra with parapophyseal centrodiapophyseal fossa subdivided by two ‘accessory’ laminae (one subvertical and visible in anterior and lateral views, the other anterodorsally oriented and visible only in lateral view); (2) anterior caudal vertebrae with laminae that converge ventrally on the anterior surface of the neural spine, not reaching the prezygapophyses and forming a ‘V-shaped’ conformation in anterior view; humerus with (3) greatly expanded proximomedial process, the proximal apex of which lies well medial to the humeral midshaft, (4) proportionally wide proximal end (proximal width: midshaft width ≈ 2.9), and (5) proximolaterally–distomedially oriented ridge bounding distal edge of ‘coracobrachialis fossa’; and pes with (6) metatarsal I with proximal dorsoventral diameter greater than the proximodistal length of the bone, (7) relatively short metatarsal III (only 1.2 times the length of metatarsal I), (8) proximal phalanges more than half as wide as their corresponding metatarsals are long, and (9) pedal unguals reduced, rugose, and distally truncated. These characters are associated with a unique combination of synapomorphies of the anterior caudal vertebrae that is observable in both known specimens: centra with (1) deeply concave anterior articular cotyles and strongly convex posterior articular condyles; (2) circular anterior articular surfaces and slightly quadrangular posterior articular surfaces; (3) anteroposteriorly concave lateral surfaces; (4) multiple vascular foramina on the lateral surfaces, ventral to the transverse processes; and (5) anteroposteriorly narrow, slightly concave ventral surfaces; transverse processes that are (6) robust, elongate, and posteroventrally directed, nearly reaching the anteroposterior level of the posterior condyle of the centrum; (7) wide and rounded at their lateral ends; and (8) ornamented by longitudinal ridges on their anteroventral margins at the approximate midlength of the process; and (9) neural arches that are anteriorly placed. *N. gonzalezparejasi* also exhibits the following distinctive morphologies: (1) humerus with markedly asymmetrical proximal margin in anterior view (nearly straight laterally but strongly expanded and rounded proximomedially); metatarsal V (2) 90 percent the length of metatarsal IV and (3) longer than metatarsal I; and (4) pedal phalangeal formula 2-2-2-2-0, with digits I–III bearing unguals.

### Description

The holotypic specimen of *Notocolossus* (UNCUYO-LD 301) preserves an almost complete anterior dorsal vertebra (Fig. 2a,b; Supplementary Fig. S1) that is missing only the lateral end of the right diapophysis and most of the right side of the neural spine. The bone is very large; if complete, it would measure approximately 1500 mm in maximum transverse dimension (i.e., width across the diapophyses), only 180 mm less than in dorsal vertebra 2 of the gigantic southern Patagonian titanosaur *Puertasaurus*^11^ (Supplementary Table S1). Moreover, the width across the diapophyses is substantially greater than in anterior dorsal vertebrae of another colossal titanosaur, *Argentinosaurus* (generally regarded as the most massive known terrestrial animal^12^), which reach only 1290 mm^5^. When considered in light of the exceptionally long, robust humerus of UNCUYO-LD 301, as well as the femoral length and body mass estimates generated from that bone (see below and Supplementary Information), the size of the dorsal vertebra of this specimen suggests that it represents an exceptionally large-bodied titanosaurian individual. Based on the positions of the parapophyses and prezygapophyses, as well on comparisons with anterior dorsal vertebrae of other titanosaurs (e.g., *Futalognkosaurus*^6^, *Mendozasaurus*^9^, *Rapetosaurus*^13^), we identify the *Notocolossus* dorsal vertebra as the second or third in the series. The centrum is opisthocoelous with a strongly convex, hemispherical anterior articular condyle, proportionally anteroposteriorly short, and considerably wider than tall (anterior transverse width/dorsoventral height = 1.36). Damage to a few areas of the anterior condyle reveals that it is internally comprised by camellate (i.e., ‘spongy’ or ‘cancellous’) bone; this is also the case for the left parapophysis and diapophysis. The posterior cotyle is strongly concave. The ventral surface of the centrum is smoothly convex, lacking a keel or fossa. A small, deep lateral pneumatic fossa (‘pleurocoel’) is located anterior to the parapophysis.

The parapophyses extend from the dorsal end of the centrum to the base of the neural arch, with their dorsoventral midline positioned at the approximate dorsoventral level of the ventral margin of the neural canal. They are large, well developed, strongly concave in anterior view, and much taller dorsoventrally than wide anteroposteriorly. Their articular facets face ventrolaterally. The parapophyseal articular facets are proportionally larger than in anterior dorsal vertebrae of *Mendozasaurus* (pers. obs.), though this discrepancy may well be due to serial variation along the dorsal column. The neural canal is large and subcircular in anterior view, slightly wider than tall. It is bordered dorsally by the intraprezygapophyseal lamina. The prezygapophyses are strongly developed, extending far anteriorly. Their facets are ovoid in dorsal contour, more than two times wider mediolaterally than long anteroposteriorly, and flat dorsally. They face dorsomedially, and their lateral ends slightly dorsally surpass the level of the diapophyses, as in the first dorsal vertebra of *Rapetosaurus*^13^. The diapophyses extend laterally well beyond the lateral margins of the prezygapophyses. The neural spine is slightly incomplete dorsally but was almost certainly low and subtriangular in anterior view.

Several neural arch laminae and fossae are evident. The parapophysis is linked to the diapophysis by the anteroposteriorly thin paradiapophyseal lamina. The laterally concave prezygoparapophyseal lamina comprises the anteromedial margin of a deep, teardrop-shaped fossa (here regarded as the parapophyseal centrodiapophyseal fossa following Wilson *et al.*^14^) that embays the anterior surface of the diapophysis and is better defined dorsally than ventrally. A thin, subvertical ‘accessory’ lamina subdivides this fossa, which extends posteromedially beyond the prezygoparapophyseal lamina, forming a deep, probably pneumatic cavity. This cavity is in turn subdivided by a second, anterodorsally–posteroventrally oriented ‘accessory’ lamina that is visible only in lateral view. The prezygapophyses are connected to the lateral margins of the diapophyses by the robust prezygodiapophyseal laminae, and to each other by the much lower, boomerang-shaped intraprezygapophyseal lamina. At least the ventral part of the anterior face of the neural spine is bisected by the thick, rugose prespinal lamina, whereas the lateral margins of the spine are comprised by the spinodiapophyseal laminae.

UNCUYO-LD 301 also includes an anterior caudal vertebra (Figs 2c-e and 3a,c,e,g), probably the third or fourth in the series based on comparisons with titanosaurs with complete, well-preserved anterior caudal sequences (e.g., *Alamosaurus*, *Baurutitan*, *Dreadnoughtus*, *Epachthosaurus*). The posterior face of its anteroposteriorly short, strongly procoelous centrum is as tall as wide, whereas the anterior face is slightly wider than tall. The subcircular anterior cotyle is substantially larger than the subquadrangular posterior condyle (Supplementary Table S1), suggesting that, in *Notocolossus*, the anterior-most caudal centra rapidly decreased in diameter posteriorly. There is no evidence of pneumatic fossae on either lateral surface of the centrum, but these surfaces are anteroposteriorly concave and pierced by several vascular foramina, as in many other sauropods. The ventral surface is anteroposteriorly narrow and gently concave. There are no ventrolateral ridges extending between the haemal arch facets, nor is there an associated midline sulcus. The transverse processes are powerfully developed and curve ventrally and posterolaterally; the complete, club-shaped right process sweeps far posteriorly, with its end approaching the anteroposterior plane of the posterior margin of the centrum. The lateral extent of the transverse process is approximately 60 percent the posterior width of the centrum. A low, rugose ridge—possibly for attachment of the M. caudofemoralis longus^15^—extends across much of the anteroventral surface of the transverse process.

The prezygapophyses have large, subcircular, dorsomedially-directed articular facets, but they are comparatively anteroposteriorly shorter than in many other titanosaurs. The postzygapophyses are correspondingly elongate, with their articular facets connected to spinopostzygapophyseal laminae that extend posteriorly beyond the remainder of the neural arch. The postzygapophyseal facets are dorsoventrally elongate, slightly concave, ventrolaterally oriented, and connected by a robust intrapostzygapophyseal lamina located dorsal to the neural canal.

The neural spine is vertically oriented and low relative to the size of the centrum. The dorsal end of the spine is wider transversely than long anteroposteriorly, and is ‘D-shaped’ in dorsal view, with a straight anterior border. In lateral view, the spine is rectangular and gently concave anteriorly approaching its anterodorsal corner. Short, low spinoprezygapophyseal laminae appear to connect the bases of the prezygapophyses to that of the neural spine. Another, better-developed pair of anterior laminae, seemingly distinct from the spinoprezygapophyseal laminae, extend the length of the neural spine, occupying the anterolateral corners of the spine dorsally but converging ventrally to form a ‘V-shaped’ conformation in anterior view. There is no clear evidence of a prespinal lamina. The posterior surface of the spine is framed by two prominent, posteriorly-projected spinopostzygapophyseal laminae that rapidly diverge from one another dorsally, becoming well separated at the approximate dorsoventral midline of the spine. A sagittally-positioned postspinal lamina spans much of the length of the posterior surface of the neural spine. Though damaged, it appears to expand markedly in transverse dimension dorsally, and seemingly does not reach the base of the spine.

The holotypic specimen UNCUYO-LD 301 also preserves the complete right humerus (Fig. 4a; Supplementary Fig. S3). It is 1760 mm in proximodistal length, with a mediolaterally expanded proximal end and a much narrower diaphysis (Table 1; Supplementary Table S1). To our knowledge, it is the longest humerus yet recovered from the Cretaceous, or for any titanosaurian, being 70 mm longer than that of the giant Egyptian titanosaur *Paralititan*^16^. Using the length of the *Notocolossus* humerus in conjunction with the stylopodial proportions of more completely preserved titanosaurs, we estimate the length of the missing femur of UNCUYO-LD 301 at 2166 mm (see Supplementary Information for details). Furthermore, no other sauropod humerus has the anatomical proportions of that of *Notocolossus*. The Proximal Humeral Robusticity, proposed herein as the ratio of proximal to midshaft mediolateral width, is nearly 2.9, which is substantially greater than that of all other titanosaurians (Table 1). As in *Futalognkosaurus*^17^, the proximal end is highly asymmetrical in anterior view, almost straight laterally but markedly proximomedially expanded and rounded medially. In *Notocolossus*, however, the proximal apex of the humerus is positioned well medial to the medial margin of the humeral midshaft. This greatly enlarged proximomedial expansion is here considered an autapomorphy of the new taxon. By contrast, previous studies^9^ have recognized that the proximal ends of other titanosauriform humeri are smoothly rounded (as in *Ligabuesaurus*), straight (e.g., *Mendozasaurus*, *Rapetosaurus*) or sigmoidal (e.g., *Opisthocoelicaudia*, *Paralititan*, *Quetecsaurus*, *Saltasaurus*) in anterior view, without the extraordinary degree of proximomedial expansion seen in *Notocolossus*.

Proximally, there is a slight proximolateral process—smaller than that present in *Epachthosaurus*, *Opisthocoelicaudia*, and *Saltasaurus*—and a shallow anteromedial fossa, possibly for the insertion of the M. supracoracoideus and M. coracobrachialis brevis, respectively^18^,^19^. This ‘coracobrachialis fossa’ is less defined than in some titanosaurs (e.g., *Paralititan*, pers. obs.), and is bounded distally by a proximolaterally–distomedially oriented crest. The deltopectoral crest is prominent, as in titanosaurs such as *Epachthosaurus*, *Futalognkosaurus*^17^, and *Mendozasaurus*, and extends approximately 41 percent of the total length of the bone. In *Neuquensaurus*^1^, *Opisthocoelicaudia*^18^, *Paralititan*^16^, and *Saltasaurus*^19^, by contrast, the crest occupies 50 percent or more of total length. The distal end of the deltopectoral crest of *Notocolossus* is medially deflected and mediolaterally thicker than the proximal end. Distally, at its anterior apex, the crest possesses a strongly developed, subcircular, centrally concave process for the attachment of the abductor musculature (i.e., M. pectoralis, M. dorsalis scapulae, M. deltoides scapularis). A strong process on the deltopectoral crest is also present in adult and juvenile specimens of *Mendozasaurus* (pers. obs.). The humeral head is prominent posteriorly, as in *Futalognkosaurus*. There is a pronounced longitudinal crest near the lateral margin of the posterior surface of the proximal end; it is approximately 300 mm in length and its distal terminus is roughly 600 mm from the proximal margin of the bone.

The diaphysis is elliptical in cross-section, with its long axis oriented mediolaterally, and measures 770 mm in minimum circumference. Based on that figure, the consistent relationship between humeral and femoral shaft circumference in associated titanosaurian skeletons that preserve both of these dimensions permits an estimate of the circumference of the missing femur of UNCUYO-LD 301 at 936 mm (see Supplementary Information). (Note, however, that the dataset that is the source of this estimate does not include many gigantic titanosaurs, such as *Argentinosaurus*^5^, *Paralititan*^16^, and *Puertasaurus*^11^, since no specimens that preserve an associated humerus and femur are known for these taxa.) In turn, using a scaling equation proposed by Campione and Evans^20^, the combined circumferences of the *Notocolossus* stylopodial elements generate a mean estimated body mass of ~60.4 metric tons, which exceeds the ~59.3 and ~38.1 metric ton masses estimated for the giant titanosaurs *Dreadnoughtus* and *Futalognkosaurus*, respectively, using the same equation (see Supplementary Information). It is important to note, however, that subtracting the mean percent prediction error of this equation (25.6% of calculated mass^20^) yields a substantially lower estimate of ~44.9 metric tons for UNCUYO-LD 301. Furthermore, Bates *et al.*^21^ recently used a volumetric method to propose a revised maximum mass of ~38.2 metric tons for *Dreadnoughtus*, which suggests that the Campione and Evans^20^ equation may substantially overestimate the masses of large sauropods, particularly giant titanosaurs. Unfortunately, however, the incompleteness of the *Notocolossus* specimens prohibits the construction of a well-supported volumetric model of this taxon, and therefore precludes the application of the Bates *et al.*^21^ method. The discrepancies in mass estimation produced by the Campione and Evans^20^ and Bates *et al.*^21^ methods indicate a need to compare the predictions of these methods across a broad range of terrestrial tetrapod taxa^21^. Nevertheless, even if the body mass of the *Notocolossus* holotype was closer to 40 than 60 metric tons, this, coupled with the linear dimensions of its skeletal elements, would still suggest that it represents one of the largest land animals yet discovered.

The radial and ulnar condyles on the distal end of the UNCUYO-LD 301 humerus are similarly developed and undivided, with the radial condyle being more poorly defined anteriorly than in some other titanosaurs (e.g., *Epachthosaurus*, *Futalognkosaurus*, *Paralititan*). The anterior face of this condyle is not divided by a notch. The posterior surface of the distal end of the humerus is badly damaged, but it bears an olecranon fossa that is bounded by supracondylar ridges, as in many other titanosaurs (e.g., *Mendozasaurus*^9^, *Paralititan*^16^).

The articulated right tarsus and pes of the referred specimen (UNCUYO-LD 302) are complete and well preserved (Fig. 4d–f; Supplementary Figs S4–S8). The astragalus is the only ossified element of the tarsus, as in all other unquestionable titanosaurians in which this skeletal region has been preserved in articulation (i.e., *Epachthosaurus*^22^, *Opisthocoelicaudia*^18^, an unidentified titanosaur from Agua del Padrillo, Argentina [UNCUYO-LD 313^23^], and another unidentified taxon from La Invernada, Argentina [Museo de la Universidad Nacional del Comahue (MUCPv-)1533^24^]). The astragalus is mediolaterally reduced and has a slightly concave lateral face for the articulation of the fibula. The anterior face has a triangular contour, and the anteroposteriorly convex distal surface articulates with the proximal ends of the metatarsals (presumably metatarsals I–IV only; Fig. 4e; Supplementary Fig. S4). The low ascending process would have articulated with a depression in the distal end of the tibia. The distal surface is strongly rugose as in other titanosaurians. The tibial face is not strongly inclined as in *Aeolosaurus*^25^. The lateral face of the astragalus exhibits a large foramen (measuring ~40 by 10 mm) near its posteroventral border.

The pes is mediolaterally asymmetrical, though less so than in other neosauropods, and includes five short, robust metatarsals that have highly rugose proximal ends. The articulated metatarsus measures approximately 450 mm in mediolateral dimension across its proximal extreme. In contrast to most other titanosauriforms (e.g., *Aeolosaurus*^25^, *Epachthosaurus*^22^, *Gobititan*^26^, *Ligabuesaurus*^27^, *Rapetosaurus*^13^, the La Invernada titanosaur^24^, New Mexico Museum of Natural History [NMMNH] P-49967 [a nearly complete distal hind limb tentatively referred to *Alamosaurus*^28^], and an unnamed Early Cretaceous titanosauriform from Siberia, Russia [Paleontological Museum, Tomsk State University (PM TGU) 16/0^29^]), metatarsals III and IV are not substantially longer than the others (Table 2; Supplementary Table S3). Indeed, in all other titanosauriforms for which the lengths of metatarsals I and III have been published, metatarsal III is at least 27 percent longer than metatarsal I; typically, it is approximately 40 percent longer (Table 2). In *Notocolossus*, by contrast, metatarsal III is only 20 percent longer than metatarsal I. The metatarsus of *Notocolossus* exhibits other distinctive features as well. The minimum mediolateral breadth of metatarsal IV is nearly 70 percent that of metatarsal I (a reversal of character 224 of Wilson^30^, regarded as a synapomorphy of the eusauropod clade Mamenchisauridae [ = ‘Omeisauridae’] + [*Jobaria* + Neosauropoda] in that analysis). Moreover, metatarsal IV is slightly longer than the other metatarsals, as in *Aeolosaurus*, *Bonitasaura*, *Epachthosaurus*, *Mendozasaurus*, the Agua del Padrillo and La Invernada titanosaurs, and NMMNH P-49967 (?*Alamosaurus*). In *Antarctosaurus wichmannianus*, *Euhelopus*, *Opisthocoelicaudia*, *Rapetosaurus*, and *Tastavinsaurus*, by contrast, metatarsal III is the longest. In *Notocolossus*, metatarsal V is also relatively long: it is 90 percent the length of metatarsal IV, and, as in all other titanosaurs except *Opisthocoelicaudia* and *Rapetosaurus*, it is longer than metatarsal I. Only the even larger pes of ?*Alamosaurus* has a proportionally longer metatarsal V (Table 2).

Metatarsals I and II are twisted about their long axes such that they are dorsoventrally (i.e., anteroposteriorly) deepest proximally and mediolaterally widest distally. Metatarsal I has a ‘D-shaped’ proximal end, and its proximal dorsoventral diameter exceeds the proximodistal length of the entire element (Supplementary Table S3). We regard these proportions as autapomorphic of *Notocolossus*. The medial margin of metatarsal I is convex, whereas the lateral margin is slightly concave for articulation with metatarsal II. The proximal outline of metatarsal II is also ‘D-shaped’, but conversely, the lateral face is slightly convex and the medial face is gently concave. Thus, the proximal contour of the articulated metatarsals I and II is subcircular. The proximal ends of metatarsals III and IV are subquadrangular in shape, whereas that of metatarsal V is slightly semilunar, with the most acute end pointing dorsolaterally. The shafts of all metatarsals are constricted both dorsoventrally and mediolaterally. Their distal ends are mediolaterally broad, and range in distal profile from quadrangular in metatarsal I to elliptical in metatarsal V. The latter is proportionally more distally expanded than in most other titanosauriforms (e.g., *Bonitasaura*, *Epachthosaurus*, *Mendozasaurus*, *Opisthocoelicaudia*, *Rapetosaurus*, *Tastavinsaurus*, the La Invernada titanosaur, PM TGU 16/0, Field Museum of Natural History [FMNH] PR 977 [an isolated titanosauriform pes from Texas^31^ referred to *Cedarosaurus* by D’Emic^32^]), with the exception of NMMNH P-49967 (?*Alamosaurus*).

The pedal phalangeal formula is 2-2-2-2-0, as in *Mendozasaurus* (pers. obs. of an associated but disarticulated specimen) and the Agua del Padrillo and La Invernada titanosaurs^23^ (Table 3). Other titanosauriforms, by contrast, differ in the number of phalanges on digits III–V. Notable differences involve digit III, where, as in UNCUYO-LD 302, most taxa (*Gobititan*, *Opisthocoelicaudia*, the Padrillo and Invernada taxa) carry two phalanges, but *Epachthosaurus* has three, and FMNH PR 977 may retain four^31^ (though the pedal phalangeal formula of this specimen has recently been reinterpreted^32^). Furthermore, *Opisthocoelicaudia* is reported to possess only a single phalanx on digit IV^18^, whereas *Gobititan* apparently retains two phalanges on digit V^26^. Phalanx I-1 of *Notocolossus* is considerably proximodistally shorter than the other proximal phalanges, but it remains large and well-developed, unlike in the Invernada titanosaur, where this phalanx is apomorphically reduced^24^. All proximal phalanges are much mediolaterally wider than dorsoventrally deep, with their widths exceeding half the lengths of their corresponding metatarsals (Supplementary Table S3). Those of digits II–IV are robust, proximodistally elongate, and quadrangular in dorsal view. The distal articular surfaces of phalanges II-1 and III-1 are bevelled such that they angle sharply proximolaterally in dorsal view, and the medial faces of these phalanges are considerably longer than the lateral. Unlike the other proximal phalanges, phalanx IV-1 is notably ‘waisted’ in dorsal view, such that it is mediolaterally narrowest at midshaft.

The appearance of the three pedal unguals (phalanges I-2, II-2, and III-2) of UNCUYO-LD 302 is unique within Sauropoda (Fig. 4d,e; Supplementary Figs S4, S5, S8). Their proximal extremes closely resemble those of the pedal unguals of other titanosaurs (e.g., *Dreadnoughtus*, *Epachthosaurus*, *Mendozasaurus*, *Rapetosaurus*, ?*Alamosaurus*, the Padrillo and Invernada forms) in being dorsoventrally elongate and elliptical in proximal view; this is especially true for ungual II. Nevertheless, each ungual terminates in a blunt, extremely rugose and irregular distal end. As preserved, unguals II and III are concave distally and longer than ungual I. Whether this condition represents the ‘typical’ morphology of *Notocolossus* or is pathological is presently unclear (see below). Phalanx IV-2 is an amorphous, proximodistally compressed bony ‘nubbin’ that is flat proximally and convex distally.

## Discussion

The fossil record of titanosaurian pedes is sparse. Only five titanosaurs are currently known from complete, articulated hind feet: *Epachthosaurus*, *Notocolossus*, *Opisthocoelicaudia*, and the unnamed Agua del Padrillo and La Invernada taxa. The holotype of the Brazilian titanosaur *Tapuiasaurus* is reported to include a nearly complete left pes^8^, but this has not been described. Many other titanosaur specimens—including some that pertain to very large-bodied individuals—include pedal elements, although none of these preserve the pes in its entirety. For example, the isolated distal hind limb NMMNH P-49967 (?*Alamosaurus*) represents a titanosaur with an estimated femoral length of 1.6–2.1 m (see D’Emic *et al.*^28^ and Supplementary Information), but its pes is missing at least two phalanges, precluding a definitive assessment of the phalangeal formula of the taxon to which it belongs. Similarly, pedal elements are known for the giant titanosaur *Dreadnoughtus*, but as these consist of only metatarsals I and II and the ungual of digit I, knowledge of the hind foot anatomy of this taxon is limited^4^. As noted above, the humerus of the *Notocolossus* holotype (UNCUYO-LD 301) is longer than that of any other titanosaur for which this element is known (Table 1); moreover, the anterior dorsal vertebra of this specimen exceeds those of *Argentinosaurus* and approaches that of *Puertasaurus* in transverse width. The estimated femoral length and body mass of UNCUYO-LD 301 are greater than those of almost all other titanosaurs (see Supplementary Information). Therefore, assuming that the referred specimen UNCUYO-LD 302 pertains to this taxon, *Notocolossus* is significant in being the largest titanosaur—and possibly the most massive terrestrial animal—for which the pedal skeleton is completely represented.

The pes of *Notocolossus* exhibits several characters that are unique within Titanosauria, or, in some cases, Sauropoda as a whole. It has short, thick metatarsals, all of which are approximately the same length (Table 2; Supplementary Table S3); among these, the relative length and robusticity of metatarsals I and V is remarkable. This morphology results in a pes that is comparatively shorter and more mediolaterally symmetrical than those of other titanosaurs, and indeed, most other sauropods – a foot in which the weight of the animal appears to have been more evenly distributed through the metatarsus. The *Notocolossus* pes differs considerably from those of other neosauropods, in which metatarsals I–IV exhibit a significant increase in length and a concomitant decrease in robusticity. In these taxa, metatarsal IV is generally 40–50 percent longer than metatarsal I; furthermore, the proximal phalanges are often proportionally less robust than are those of *Notocolossus*. In these neosauropods, the hind foot is strongly entaxonic (i.e., more robustly constructed medially than laterally), and weight was presumably borne primarily by the first three digits. Given the enormous size of *Notocolossus*, its distinctive, relatively homogeneous pedal morphology may constitute an adaptation for supporting a greatly elevated body mass. The fact that the only other sauropod specimen with a comparably robust and elongate metatarsal V is the even larger titanosaurian pes NMMNH P-49967 (?*Alamosaurus*) is consistent with this interpretation (although the metatarsus of that specimen differs in other respects from that of *Notocolossus*).

A number of pedal morphologies evident in *Notocolossus* (e.g., metatarsal I with proximal dorsoventral diameter greater than the proximodistal length of the bone, metatarsal III only 1.2 times the length of metatarsal I, proximal phalanges more than half as wide as their corresponding metatarsals are long, pedal unguals reduced, rugose, and distally truncated) are currently unique within Titanosauria. Because the titanosaurian fossil record is highly incomplete, and many taxa do not preserve much (or, in some cases, any) pedal material, it has yet to be established whether the unusual morphology of the *Notocolossus* pes is diagnostic of this taxon, or, alternatively, if it characterized a more inclusive titanosaur clade. However, the new taxon indicates that titanosaurian morphological diversity was even greater than previously appreciated, and that members of this group exhibited at least two principal pedal morphotypes: (1) comparatively short, robust, and mediolaterally symmetrical (as in *Notocolossus*), and (2) elongate and strongly entaxonic (e.g., *A*. *wichmannianus*, *Bonitasaura*, *Epachthosaurus*, *Rapetosaurus*, ?*Alamosaurus*, the Agua del Padrillo and La Invernada titanosaurs). Given the remarkable diversity in body size and proportions within Titanosauria (e.g., relatively small forms such as *Magyarosaurus* and *Saltasaurus* versus gigantic taxa like *Argentinosaurus*, *Notocolossus*, and *Puertasaurus*; long-necked titanosaurs such as *Rapetosaurus* versus short-necked forms such as *Isisaurus* and *Mendozasaurus*), it is not surprising that pedal structure also varies appreciably between different members of the clade.

Among sauropods, the pedal unguals of *Notocolossus* are unique in being unusually short and distally truncated. Although, as mentioned above, it is possible that their peculiar appearance is pathological—pathologies have been documented in sauropod pedes before^33^,^34^—we consider this less likely because all three unguals exhibit similar morphologies and there is no evidence of pathology in the other pedal elements. Nevertheless, additional pedal material of *Notocolossus* will be required to evaluate this hypothesis.

**Figure 5:**
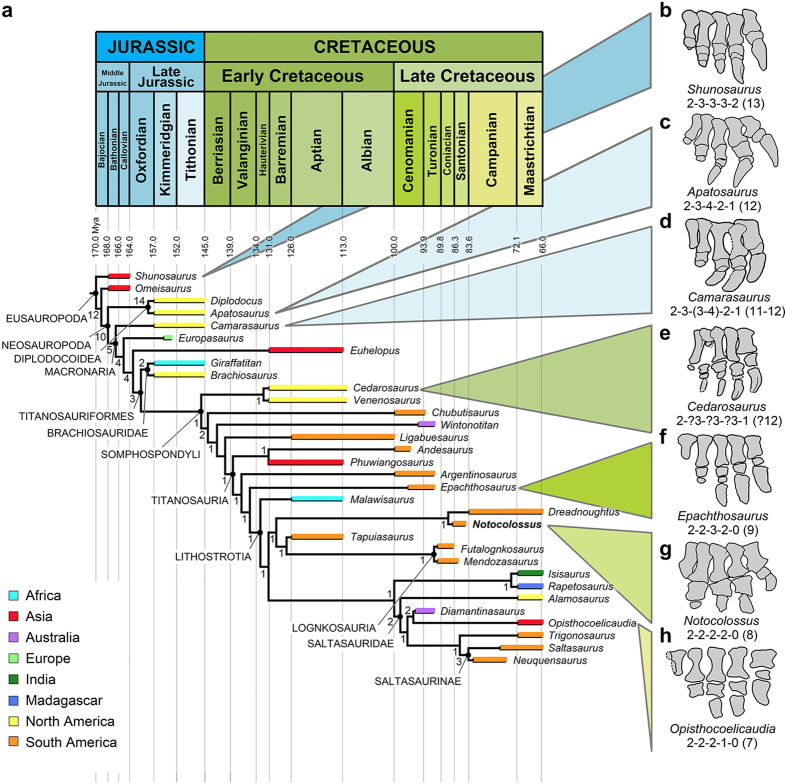
Hypothesized phylogenetic position of *Notocolossus gonzalezparejasi* and pedal evolution of Sauropoda. (**a**) Time-calibrated hypothesis of phylogenetic relationships of *Notocolossus* with relevant clades labelled. Depicted topology is that of the single most parsimonious tree of 720 steps in length (Consistency Index = 0.52; Retention Index = 0.65). Stratigraphic ranges (indicated by coloured bars) for most taxa follow Lacovara *et al.*^4^: fig. 3 and references therein. Additional age sources are as follows: *Apatosaurus*^55^, *Cedarosaurus*^58^, *Diamantinasaurus*^59^, *Diplodocus*^35^, *Europasaurus*^35^, *Ligabuesaurus*^35^, *Neuquensaurus*^60^, *Omeisaurus*^55^, *Saltasaurus*^60^, *Shunosaurus*^55^, *Trigonosaurus*^35^, *Venenosaurus*^58^, *Wintonotitan*^59^. Stratigraphic ranges are colour-coded to also indicate geographic provenance of each taxon: Africa (excluding Madagascar), light blue; Asia (excluding India), red; Australia, purple; Europe, light green; India, dark green; Madagascar, dark blue; North America, yellow; South America, orange. (**b–h**) Drawings of articulated or closely associated sauropod right pedes in dorsal (=anterior) view, with respective pedal phalangeal formulae and total number of phalanges per pes provided (the latter in parentheses). (**b**) *Shunosaurus* (ZDM T5402, reversed and redrawn from Zhang^45^); (**c**) *Apatosaurus* (CM 89); (**d**) *Camarasaurus* (USNM 13786); (**e**) *Cedarosaurus* (FMNH PR 977, reversed from D’Emic^32^); (**f**) *Epachthosaurus* (UNPSJB-PV 920, redrawn and modified from Martínez *et al.*^22^); (**g**) *Notocolossus*; (**h**) *Opisthocoelicaudia* (ZPAL MgD-I-48). Note near-progressive decrease in total number of pedal phalanges and trend toward phalangeal reduction on pedal digits II–V throughout sauropod evolutionary history (culminating in phalangeal formula of 2-2-2-1-0 [seven total phalanges per pes] in the latest Cretaceous derived titanosaur *Opisthocoelicaudia*). Abbreviation: Mya, million years ago. Institutional abbreviations see Supplementary Information.

To further investigate the distinctive pedal morphology of *Notocolossus* within an evolutionary context, we conducted a phylogenetic analysis. The analysis was based primarily on that of Carballido and Sander^35^ and references therein, but also incorporated nine additional (including four newly formulated) characters and four more titanosaurs while excluding a large number of non-titanosaurian taxa (see Methods and Supplementary Information for details). The analysis yielded a single most parsimonious tree (Fig. 5a), the topology of which is consistent in most respects with those advanced by other recent studies^30^,^35^,^36^,^37^,^38^. Here, we focus on proposed relationships within Titanosauria, which we treat as a node-based group following the definition proposed by Salgado *et al.*^39^ and subsequently modified by Wilson and Upchurch^40^. *Argentinosaurus* and *Epachthosaurus* are resolved as basal titanosaurians and successively proximal outgroups to the node-based Lithostrotia, the definition of which follows Upchurch *et al.*^36^. *Malawisaurus* is the basal-most lithostrotian; the remaining members of this group are distributed among two unnamed clades, here informally termed lithostrotian ‘clade A’ and ‘clade B’. Within ‘clade A’, *Notocolossus* is recovered as the sister taxon of the recently-described Patagonian titanosaur *Dreadnoughtus*; this relationship is supported by three synapomorphies of the humerus (deltopectoral crest markedly expanded distally, distal condyle exposed on anterior portion of humeral shaft, distal condyle flat). In turn, the *Dreadnoughtus* + *Notocolossus* clade forms the sister group to *Tapuiasaurus* + Lognkosauria (i.e., *Futalognkosaurus* + *Mendozasaurus*). Interestingly, and perhaps not coincidentally, except for *Tapuiasaurus*, all members of this newly recognized South American lithostrotian clade are gigantic, with humeral lengths in excess of 1.56 m (*Dreadnoughtus*, *Futalognkosaurus*, *Notocolossus*) and/or femoral lengths greater than 1.8 m (*Dreadnoughtus*^4^, *Futalognkosaurus*^17^, pers. obs. of undescribed *Mendozasaurus* specimen^9^). In Lacovara *et al.*’s^4^ analysis, conversely, both *Dreadnoughtus* and Lognkosauria were excluded from Lithostrotia. Within ‘clade B’, an Indo-Madagascan *Isisaurus* + *Rapetosaurus* subclade is the sister taxon of Saltasauridae.

Previous authors^24^,^41^,^42^,^43^ have noted that, in general, the evolution of the sauropod hind foot is characterized by an overall reduction in the number and proportional size of the phalanges. Indeed, when pedal morphology is mapped onto our phylogenetic hypothesis (Fig. 5b–h; Table 3), an intriguing trend emerges: namely that, through the reduction of digits II–V, sauropods appear to have reduced their total number of pedal phalanges in a nearly progressive fashion over the course of their evolutionary history. The basal eusauropods *Shunosaurus* and *Omeisaurus* have a pedal phalangeal formula of 2-3-3-3-2, for a total of 13 phalanges in each pes^44^,^45^. By contrast, the phalangeal formulae of the diplodocids *Apatosaurus*, *Diplodocus*, and an indeterminate diplodocine are 2-3-4-2-1 (12 total phalanges^46^), 2-3-3-2-2 (12 phalanges^47^), and 2-3-3-2-1 (11 phalanges^48^), respectively, whereas those of the basal macronarians *Camarasaurus* and *Janenschia* are respectively 2-3-(3 or 4)-2-1 (11–12 phalanges^49^) and 2-3-3-2-1 (11 phalanges^50^,^51^). This suggests that an initial phase of phalangeal reduction involving the lateral-most two digits may have taken place at or in the vicinity of the origin of Neosauropoda (as was previously noted by Upchurch^41^ in the case of digit IV). In this context, the reported presence of phalanx V-2 in *Diplodocus* and the Chinese Early Cretaceous macronarian *Gobititan*^26^ is interpreted as an autapomorphic reversal. The pedal phalangeal formula of the latter taxon is stated as 2-2-2-2-2 (ten phalanges). The affinities of *Gobititan* are not well understood; nevertheless, most authors^37^,^52^ have regarded this genus as a non-titanosaurian titanosauriform. Its pedal morphology therefore suggests that (1) the trend toward reduction in total phalangeal number continued within Titanosauriformes, and (2) phalanx II-3 was permanently lost prior to the origin of Titanosauria.

Among sauropods, pedal phalangeal reduction reached its extreme in Titanosauria. The pedal phalangeal formula of the basal titanosaurian *Epachthosaurus* is 2-2-3-2-0 (nine phalanges); consequently, the loss of phalanges on digit V appears to have taken place at or near the origin of this clade. Moreover, the phalangeal formulae of titanosaurs indicate a progressive reduction of the total number of phalanges via the loss of phalanges on pedal digits III and IV through the evolution of these dinosaurs^24^. The phalangeal formulae of the basal lithostrotians *Notocolossus* and (probably) *Mendozasaurus* are 2-2-2-2-0 (eight), whereas that of the saltasaurid *Opisthocoelicaudia* is 2-2-2-1-0 (seven). As such, it appears that a single phalanx was lost from digit III at or near the origin of Lithostrotia; similarly, a digit IV phalanx may have been lost in the vicinity of Saltasauridae. Pedal digit I was reduced in at least one titanosaur as well: although, as in *Mendozasaurus* and *Notocolossus*, the unnamed La Invernada taxon has a phalangeal formula of 2-2-2-2-0, the proximal-most phalanx of digit I is strikingly small^24^.

Because the definitive pedal phalangeal formulae of most sauropods remain unknown, additional discoveries may well alter the pattern observed herein; i.e., the reduction in the total number of pedal phalanges over the course of sauropod evolution may eventually be shown to be less ‘progressive’ than it currently appears. Nevertheless, the tendency toward phalangeal reduction through sauropod evolutionary history is striking when viewed in light of pedal evolutionary trends in proboscidean mammals, another tetrapod clade that produced exceptionally large-bodied, graviportal representatives. Rather than a reduced number of pedal bones, extant elephants have substantially increased this number over the condition in their earlier-diverging relatives through the retention of most phalanges and the addition of ossified sesamoids (‘predigits’)^53^. These opposing trends in pedal ossification in proboscideans and sauropods add to the numerous distinctions already noted between the pedes of these animals (e.g., sub-unguligrade posture in elephants versus semi-plantigrade posture in sauropods)^42^, underscoring the fact that different vertebrate clades have evolved distinct osteological solutions for supporting massive body weights in terrestrial environments.

Derived titanosaurs had the most reduced pedes of all sauropods, with the fewest number of phalanges. Interestingly, it has long been recognized^39^ that the manus of titanosaurs was the most reduced within Sauropoda as well, with almost all taxa for which this skeletal region is completely known exhibiting only a rudimentary phalanx on manual digit IV (*Epachthosaurus*, *Opisthocoelicaudia*) or no manual phalanges at all (e.g., *Alamosaurus*^54^, the La Invernada titanosaur^24^). This raises the intriguing possibility that phalangeal reduction on both the titanosaurian manus and pes may have been due to common functional, behavioural, or even genetic factors. Evaluation of this hypothesis must await the description of additional titanosaurian specimens, ideally skeletons that preserve both the manus and pes in their entirety. In the interim, this much is clear: the titanosaur *Notocolossus*, one of the largest terrestrial vertebrates ever discovered, exhibits an extreme case of reduced yet robustly constructed pedes, a morphology that is, to date, unique among sauropods.

## Methods

### Phylogenetic analysis

We conducted a phylogenetic analysis to assess the affinities of *Notocolossus gonzalezparejasi* within Titanosauria and to evaluate the significance of the pedal morphologies of this taxon in an evolutionary context. We assembled a matrix of 33 taxa (32 sauropod ingroups plus the basal sauropodomorph *Plateosaurus engelhardti* as an outgroup) scored for 350 morphological characters (see Supplementary Information). In choosing ingroup taxa, we placed emphasis on including a diversity of titanosaurs (especially gigantic titanosaurs) and sauropods for which the pes is well represented. The vast majority (341) of the characters employed were taken from Carballido and Sander^35^ and sources therein, but one of these (number 133) was slightly modified from that analysis, and the literature attributions of a few other characters were corrected. Two characters (131 and 132) were taken from González Riga and Ortiz David^38^ and sources therein, whereas character 257 was modified from Mannion *et al.*^55^. Character 258 was modified from Curry Rogers^1^ and character 350 was modified from Upchurch^41^. The most significant contribution of the present phylogenetic analysis is the addition of four newly formulated characters (numbers 331, 334, 348, and 349) that pertain to pedal morphology (see Supplementary Information). We analysed the matrix using the methods outlined in Carballido and Sander^35^; specifically, 24 characters (12, 58, 95, 96, 102, 106, 108, 115, 116, 119, 120, 156, 166, 215, 218, 234–237, 260, 271, 302, 303, and 305) were treated as ordered, and the matrix was subjected to a heuristic (traditional) search in TNT (Tree analysis using New Technology) v. 1.1^56^ (1000 replicates of Wagner trees, random addition sequence, tree bisection reconnection branch swapping algorithm, ten trees saved per replicate). Note that the three characters that pertain to pedal phalangeal reduction (numbers 348–350) were treated as unordered. The analysis yielded a single most parsimonious tree of 720 steps (Consistency Index = 0.52; Retention Index = 0.65) the topology of which is shown in Fig. 5a. An identical result was obtained using the heuristic analysis function of NONA v. 2.0^57^.

### Data archiving

Data reported in this paper are available as Supplementary Information. Specimens UNCUYO-LD 301 and 302 are reposited at the Laboratorio de Dinosaurios of the Facultad de Ciencias Exactas y Naturales of the Universidad Nacional de Cuyo in Mendoza City, Mendoza Province, Argentina.

### Nomenclatural acts

This published work and the nomenclatural acts it contains have been registered in ZooBank, the proposed online registration system for the International Code of Zoological Nomenclature (ICZN). The ZooBank LSIDs (Life Science Identifiers) can be resolved and the associated information viewed through any standard web browser by appending the LSID to the prefix ‘ http://zoobank.org/’. The LSIDs for this publication are urn:lsid:zoobank.org:act:01FFA8B8-BA10-4D80-BB94-4CB3071597B2 (*Notocolossus*) and urn:lsid:zoobank.org:act:34979D66-9C7E-469C-B072-AB9FB8F6B705 (*N*. *gonzalezparejasi*).

## Additional Information

**How to cite this article**: González Riga, B. J. *et al.* A gigantic new dinosaur from Argentina and the evolution of the sauropod hind foot. *Sci. Rep.* **6**, 19165; doi: 10.1038/srep19165 (2016).

## Supplementary Material

Supplementary Information

## Figures and Tables

**Table 1:** Proximodistal length and proximal and midshaft mediolateral width (mm) of the humerus of the holotype of *Notocolossus gonzalezparejasi* (UNCUYO-LD 301) compared to those of other titanosaurian sauropods, including other gigantic species.

Species	Specimen	Length	Width, proximal	Width, midshaft	PHR	Source(s)
*Notocolossus gonzalezparejasi*	UNCUYO-LD 301	1760	720	250	2.88	this paper
*Paralititan stromeri*	CGM 81119	1690	562	234	2.40	pers. obs.
Titanosauria indet.	MMCH NA	1660	NA	NA	—	61
*Dreadnoughtus schrani*	MPM-PV 1156	1600	740	320	2.31	4
*Futalognkosaurus dukei*	MUCPv-323	1560	600	250	2.40	17; pers. obs.
*Alamosaurus sanjuanensis*	TMM 41541-1	1503	NA	NA	—	62
*Alamosaurus sanjuanensis*	USNM 15560	1360	NA	230	—	54
*Mendozasaurus neguyelap*	IANIGLA-PV 069	1060	350	145	2.41	9
*Opisthocoelicaudia skarzynskii*	ZPAL MgD-I/48	1000a	540a	220a	2.45	18
*Epachthosaurus sciuttoi*	UNPSJB-PV 920	910a	310a	163a	1.90	22
*Rapetosaurus krausei*	FMNH PR 2209	524	203	86	2.36	13

(See Supplementary Table S1 for additional measurements of the *Notocolossus* humerus.) Specimens are listed by decreasing humeral length. The *Notocolossus* humerus is the longest yet reported for Titanosauria, and also exhibits the greatest Proximal Humeral Robusticity (i.e., ratio of proximal to midshaft width) value known within this sauropod clade. Abbreviations: NA, not available (i.e., not preserved or not reported); PHR, Proximal Humeral Robusticity. Institutional abbreviations see Supplementary Information. a = measurement averaged from left and right elements of specimen in question; — = calculation not possible based on available data.

**Table 2:** Proximodistal lengths (mm) of the metatarsals of the referred specimen of *Notocolossus gonzalezparejasi* (UNCUYO-LD 302) compared to those of other titanosauriform sauropods.

Species	Specimen	I	II	III	IV	V	III/I	IV/I	V/III	V/IV	Source
*Notocolossus gonzalezparejasi*	UNCUYO-LD 302	164	185	197	218	196	1.20	1.33	0.99	0.90	this paper
*Mendozasaurus neguyelap*	IANIGLA-PV 077	140	156	178	205	165	1.27	1.46	0.93	0.81	9
*Opisthocoelicaudia skarzynskii*	ZPAL MgD-I/48	150	180	200	180	140	1.33	1.20	0.70	0.78	18
Agua del Padrillo titanosaur	UNCUYO-LD 313	109	138	146	152	130	1.34	1.39	0.89	0.86	this paper
?*Alamosaurus sanjuanensis*	NMMNH P-49967	195	245	270	291	281	1.38	1.49	1.04	0.97	28
*Euhelopus zdanskyi*	PMU 234	98	122	136	121	NA	1.39	1.23	—	—	52
La Invernada titanosaur	MUCPv-1533	120	137	168	172	127	1.40	1.43	0.76	0.74	24
*Bonitasaura salgadoi*	MPCA 460	120	154	169	180	142	1.41	1.50	0.84	0.79	64
*Rapetosaurus krausei*	FMNH PR 2209	63	82	89	88	59	1.41	1.40	0.66	0.67	13
*Epachthosaurus sciuttoi*	UNPSJB-PV 920	125	153	177	185	153	1.42	1.48	0.86	0.83	22
*Tastavinsaurus sanzi*	MPZ 99/9	162	190	230	212	180	1.42	1.31	0.78	0.85	65
*Ligabuesaurus leanzai*	MCF-PVPH-233	140	190	220	220	180	1.57	1.57	0.82	0.82	27
*Antarctosaurus wichmannianus*	MACN-PV 6904	140	200	225	215	—	1.61	1.54	—	—	63
*Dreadnoughtus schrani*	MPM-PV 1156	210	250	NA	NA	NA	—	—	—	—	4
*Cedarosaurus weiskopfae*	FMNH PR 977	120	200	165*	NA	200	—	—	—	—	31

(See Supplementary Table S3 for additional measurements of the *Notocolossus* metatarsus.) Specimens are listed by increasing metatarsal III to metatarsal I length ratio. Note that (1) although probable, it is not certain that all metatarsals of *Mendozasaurus* specimen IANIGLA-PV 077 pertain to a single individual^9^, and (2) although Huene^63^:73 provided a length measurement (120 mm) for a purported metatarsal V of the *Antarctosaurus wichmannianus* holotype, this element may actually represent metatarsal I of another individual; as such, we have omitted this measurement here. Abbreviations: I–V, metatarsal number; III/I, metatarsal III to metatarsal I length ratio; IV/I, metatarsal IV to metatarsal I length ratio; V/III, metatarsal V to metatarsal III length ratio; V/IV, metatarsal V to metatarsal IV length ratio; NA, not available (i.e., element not preserved or measurement not reported). Institutional abbreviations see Supplementary Information. * = element incomplete, measurement as preserved; — = calculation not possible based on available data.

**Table 3:** Pedal phalangeal formulae and total number of pedal phalanges of sauropods for which complete hind feet are known.

Higher taxon/species	Specimen(s)	I	II	III	IV	V	Sum	Source(s)
Eusauropoda								
* Shunosaurus lii*	ZDM T5402	2	3	3	3	2	13	45
* Omeisaurus tianfuensis*	ZDM T5701, T5704	2	3	3	3	2	13	44
Neosauropoda								
* Apatosaurus* sp.	CM 89	2	3	4	2	1	12	46
* Diplodocus hallorum*	USNM 10865	2	3	3	2	2	12	47
* *FS Quarry diplodocine	WDC-FS001A	2	3	3	2	1	11	48
Macronaria								
* Janenschia robusta*	SMNS 12144	2	3	3	2	1	11	50,51
* Camarasaurus lentus*	USNM 13786	2	3	3	2	1	11	49
Titanosauriformes								
* Gobititan shenzhouensis*	IVPP 12579	2	2	2	2	2	10	26
Titanosauria								
* Epachthosaurus sciuttoi*	UNPSJB-PV 920	2	2	3	2	0	9	22
Lithostrotia								
* Notocolossus gonzalezparejasi*	UNCUYO-LD 302	2	2	2	2	0	8	this paper
* Mendozasaurus neguyelap*	IANIGLA-PV 077	2	2	2	2	0	8	pers. obs.
* *Agua del Padrillo titanosaur	UNCUYO-LD 313	2	2	2	2	0	8	23
* *La Invernada titanosaur	MUCPv-1533	2	2	2	2	0	8	24
Saltasauridae								
* Opisthocoelicaudia skarzynskii*	ZPAL MgD-I/48	2	2	2	1	0	7	18

Specimens are listed by decreasing total number of phalanges. Higher taxonomic assignment follows Fig. 5a for taxa included therein, Mannion *et al.*^55^: fig. 22 for *Janenschia* and *Gobititan*, and González Riga *et al.*^23^,^24^ for the Agua del Padrillo and La Invernada titanosaurs. Note overall decrease in total phalangeal number through sauropod evolution, and apparently progressive loss of phalanges on digits III and IV within Titanosauria. Abbreviations: I–V, digit number. Institutional abbreviations see Supplementary Information.
