# Supplementary material for: A gigantic new dinosaur from Argentina and the evolution of the sauropod hind foot
Source: Sci Rep. 2016 Jan 18;6:19165. doi: 10.1038/srep19165 (PMC4725985; doi:10.1038/srep19165)
Supplement: Supplementary Information [file srep19165-s1.pdf]

A gigantic new dinosaur from Argentina and the evolution of the sauropod hind foot

Bernardo J. González Riga, Matthew C. Lamanna, Leonardo D. Ortiz David, Jorge O. Calvo, Juan P. Coria

Supplementary Information:

I. Institutional abbreviations

II. Geologic context

III. Justification for referral of UNCUYO-LD 302 to *Notocolossus gonzalezparejasi*

IV. Supplemental description

V. Comparison with other Plottier Formation titanosaurs

VI. Comparison with *Mendozasaurus neguyelap*

VII. Estimates of body dimensions

VIII. Photogrammetric models

IX. Phylogenetic character list

X. Phylogenetic data matrix

XI. Supplementary Figures S1–S11

XII. Supplementary Tables S1–S6

XIII. References for Supplementary Information

I. Institutional abbreviations

AODF, Australian Age of Dinosaurs Natural History Museum, Winton, Australia; **BSPG**, Bayerische Staatssammlung für Paläontologie und Geologie, Munich, Germany; **CGM**, Egyptian Geological Museum, Cairo, Egypt; **CM**, Carnegie Museum of Natural History, Pittsburgh, U.S.A.; **DMNH**, Denver Museum of Nature and Science, Denver, U.S.A.; **FMNH**, Field Museum of Natural History, Chicago, U.S.A.; **IANIGLA-PV**, Instituto Argentino de Nivología, Glaciología y Ciencias Ambientales, Colección Paleovertebrados, Mendoza, Argentina; **IVPP**, Institute of Vertebrate Paleontology and Paleoanthropology, Beijing, P.R. China; **MACN-PV**, Museo Argentino de Ciencias Naturales, Colección de Paleontología de Vertebrados, Buenos Aires, Argentina; **MAU**, Museo Argentino Urquiza, Rincón de Los Sauces, Argentina; **MCF-PVPH**, Museo Carmen Funes, Plaza Huincul, Argentina; **MLP**, Museo de La Plata, La Plata, Argentina; **MMCH**, Museo Municipal Ernesto Bachmann, Villa El Chocón, Argentina; **MPCA**, Museo Provincial Carlos Ameghino, Cipolletti, Argentina; **MPM**, Museo Padre Molina, Río Gallegos, Argentina; **MPZ**, Museo Paleontológico de la Universidad de Zaragoza, Zaragoza, Spain; **MUCPv**, Museo de la Universidad Nacional del Comahue, Neuquén, Argentina; **NHMUK**, Natural History Museum, London, U.K.; **NMMNH**, New Mexico Museum of Natural History, Albuquerque, U.S.A.; **PM TGU**, Paleontological Museum, Tomsk State University, Tomsk, Russia; **PMU**, Palaeontological Museum, Uppsala, Sweden; **PVL**, Fundación-Instituto

Miguel Lillo, Tucumán, Argentina; **SMNS**, Staatliches Museum für Naturkunde, Stuttgart, Germany; **TMM**, Texas Memorial Museum, Austin, U.S.A.; **UNCUYO-LD**, Universidad Nacional de Cuyo, Laboratorio de Dinosaurios, Mendoza, Argentina; **UNPSJB-PV**, Universidad Nacional de la Patagonia San Juan Bosco, Paleontología de Vertebrados, Comodoro Rivadavia, Argentina; **USNM**, National Museum of Natural History, Washington, D.C., U.S.A.; **WDC**, Wyoming Dinosaur Center, Thermopolis, U.S.A.; **ZDM**, Zigong Dinosaur Museum, Zigong, P.R. China; **ZPAL**, Institute of Paleobiology, Polish Academy of Sciences, Warsaw, Poland.

II. Geologic context

The two specimens of *Notocolossus gonzalezparejasi* were collected from Upper Cretaceous sediments exposed in southern-most Mendoza Province, Argentina (Fig. 1a). Dinosaur remains are abundant in this part of Mendoza. To date, the record includes three titanosauriform sauropods in addition to the taxon described here (the titanosaurs *Mendozasaurus neguyelap*[1–3] and *Quetecsaurus rusconii*[4] and the non-titanosaurian somphospondylan *Malarguesaurus florenciae*[5]) and the megaraptoran theropod *Aerosteon riocoloradensis*[6]. An abundance of undescribed material pertains to at least one additional titanosaurian taxon (represented in part by specimen UNCUYO-LD 313[7]) plus one or more abelisauroid theropods, pterosaurs, chelid turtles, and dipnoan and teleost fishes[8–10].

In Mendoza, the most fossiliferous Cretaceous sedimentary sequences were deposited in the Neuquén Basin, which extends between the active magmatic arc along the Andes to the west, the Sierra Pintada System to the northeast, and the North Patagonian Massif to the southeast. Sediments within the basin consist of marine, littoral, and continental deposits related to transgressive–regressive cycles of both the Pacific and the Atlantic oceans[11,12]. The richest titanosaurian record in South America comes from the Neuquén Basin, primarily from Upper Cretaceous strata of the Neuquén Group and the Allen Formation[13–16].

The Upper Cretaceous (Cenomanian–lower Campanian) Neuquén Group is the most important dinosaur-bearing unit in the Neuquén Basin[13]. It comprises a thick succession of continental sandstones, conglomerates, and claystones that represent alluvial fan, fluvial, and playa lake depositional environments[17], and is divided into the Río Limay, Río Neuquén, and Río Colorado subgroups[18]. Recently, Garrido[19] recognized two new geologic units, the Los Bastos and Sierra Barrosa formations, from deposits previously assigned to the Portezuelo Formation of the Río Neuquén Subgroup. The Portezuelo, Los Bastos, and Sierra Barrosa formations collectively range from late Turonian to late Coniacian in age.

In Mendoza and Neuquén provinces, the change of fluvial facies of the Neuquén Group renders the correlation of formations and members difficult[3]; however, detailed stratigraphic observations of the region allow us to place the various titanosauriform discoveries in context. The holotype of *Quetecsaurus* was discovered in red mudstones of the middle–upper Turonian Cerro Lisandro Formation exposed in the Cañada del Pichanal, whereas *Malarguesaurus* comes from the lower–middle Coniacian Los Bastos Formation of the Paso de las Bardas area 20 km to the west. *Mendozasaurus* was discovered south of Cerro Guillermo, in overbank facies of sandy fluvial systems corresponding to the top of the middle–upper Coniacian Sierra Barrosa Formation.

The *Notocolossus* specimens come from the same area in the northern part of Cerro Guillermo. They were found 403 m apart in the same, basal-most stratigraphic horizon—a red mudstone facies—of the upper Coniacian–lower Santonian Plottier Formation. Based on the contacts of the strata that crop out in this area, the specimens were deposited simultaneously under the same sedimentological regime. Each specimen represents a single individual. Following the sizes of their respective anterior caudal vertebrae and the inferred lengths of their femora (see below), the holotype (UNCUYO-LD 301) pertains to a larger individual than the referred specimen (UNCUYO-LD 302).

III. Justification for referral of UNCUYO-LD 302 to *Notocolossus gonzalezparejasi*

The referral of UNCUYO-LD 302 to *Notocolossus gonzalezparejasi* is based on a unique combination of synapomorphic characters of the anterior caudal vertebrae that this specimen shares with the holotype of this species (UNCUYO-LD 301). To our knowledge, no other titanosaurian anterior caudal vertebrae exhibit the following character combination that is present in these two specimens: centra with (1) deeply concave anterior articular cotyles and strongly convex posterior articular condyles (i.e., strong procoely); (2) circular anterior articular surfaces and slightly quadrangular posterior articular surfaces; (3) anteroposteriorly concave lateral surfaces; (4) multiple vascular foramina on the lateral surfaces ventral to the transverse processes; and (5) anteroposteriorly narrow, slightly concave ventral surfaces; transverse processes that are (6) robust, elongate, and posteroventrally directed, nearly reaching the anteroposterior level of the posterior condyle of the centrum; (7) wide and rounded at their lateral ends; and (8) ornamented by longitudinal ridges on their anteroventral margins at the approximate midlength of the process; and (9) neural arches that are anteriorly placed. Qualitatively, the anterior caudal vertebrae of UNCUYO-LD 301 and 302 are nearly indistinguishable (see Fig. 3), with the few differences between them presumably attributable to the larger body size of the holotype and/or the more anterior position of the only preserved caudal vertebra of this individual (Fig. 1b). Furthermore, the two specimens are almost identical in stratigraphic and geographic provenance; as indicated above, they were recovered from the same bed of the Upper Cretaceous Plottier Formation at sites only 403 m apart.

IV. Supplemental description

The referred specimen of *Notocolossus* (UNCUYO-LD 302) preserves an articulated series of seven partial anterior caudal vertebrae and seven incomplete haemal arches (Fig. 2f,g,h; Fig. 3b,d,f,h; Supplementary Fig. S2; Supplementary Table S2). Except for their smaller size (which is due in part to their slightly more posterior position in the series), the anterior-most vertebrae are nearly identical to the anterior caudal vertebra of UNCUYO-LD 301. The centra exhibit the vascular foramina evident in the latter, and the transverse processes are elongate and swept strongly posterolaterally; furthermore, the complete left transverse processes of the anterior-most three vertebrae exhibit a rugose anteroventral ridge, as in UNCUYO-LD 301 (Fig. 3). The centrum of the anterior-most and most complete vertebra of the referred specimen is quadrangular in posterior view, and its posterior articular condyle is offset dorsally (Fig. 2f,g,h,i). Its transverse process is approximately 60 percent as long as the posterior end of the centrum is wide, as is also the case in the anterior caudal of the holotype. The centra rapidly

become less strongly procoelous posteriorly. Their ventral faces are slightly concave anteroposteriorly and relatively narrow (Fig. 3).

One of the haemal arches is nearly complete, but the remaining six are fragmentary. All are proximally open ('unbridged'), lack ridges on their lateral surfaces, and seem unusually elongate relative to the sizes of the centra. The most anteriorly-positioned of these haemal arches corresponds to the anterior part of the tail (Supplementary Fig. S2a,c,e). It includes both proximal rami, the proximal articular surfaces of which exhibit a central groove (which is better preserved on the right side) such that they are separated into distinct anterior and posterior portions, as in *Mendozasaurus*[1]. In lateral view, the proximal-most part of each ramus curves posteriorly. In more posteriorly-situated haemal arches, the proximal facets have a single articular surface that is subtriangular in proximal contour (Supplementary Fig. S2b,d,f). In the nearly complete haemal arch (the third in the preserved sequence), the depth of the haemal canal is 40–45% the total proximodistal length of the bone, as is the case in many other titanosauriforms (Supplementary Table S2). The distal blades are straight.

The holotype of *Notocolossus* (UNCUYO-LD 301) preserves the proximal end of the left pubis, including structures such as the iliac peduncle and obturator foramen (Fig. 4b,c; Supplementary Table S1). The iliac peduncle is subtriangular in proximal view, broader anteroposteriorly than mediolaterally. The obturator foramen is proximodistally elongate and slit-like in lateral view, though its morphology has probably been modified by crushing.

Although the complete right tarsus and pes of the referred specimen (UNCUYO-LD 302) were disarticulated during preparation, a cast was made prior to the initiation of this process (Supplementary Fig. S4). This cast, as well as photos and field observations, demonstrates that all five metatarsals were preserved in contact at their proximal ends. As articulated, the orientations of the proximodistal axes of the metatarsals differed dramatically from one another. Metatarsal I was strongly inclined, such that its proximal end was situated much more dorsally and laterally than its distal end. Metatarsal II was oriented in generally the same fashion but tilted less steeply. In sharp contrast, metatarsals III–V were oriented essentially vertically in the mediolateral plane but angled strongly proximoventrally–distodorsally in the dorsoventral plane. This proximoventral–distodorsal angle was at least 60° in metatarsal III and approximately 45° in metatarsals IV and V. Thus, in the field, and probably also in life, the metatarsals of *Notocolossus* were arrayed in a semi-plantigrade conformation, as in other sauropods. The proximal phalanges were slightly displaced with respect to the distal ends of their respective metatarsals.

The articulated tarsus and pes were found 60 cm beneath the surface of the modern outcrop (Supplementary Fig. S5). As such, we regard the peculiar morphology of the pedal unguals as authentic, either the 'normal' condition for *Notocolossus* or a pathology of this particular individual. The remainder of the pes, including the diminutive phalanx IV-2, was also well preserved. None of the other phalanges possess the truncated, highly irregular distal surfaces evident in the three unguals.

V. Comparison with other Plottier Formation titanosaurs

Although this situation is beginning to change, the fossil vertebrate record of the Plottier Formation is, at present, relatively poorer than those of most other geologic units of the Neuquén

Group[13]. Nevertheless, *Notocolossus gonzalezparejasi* is at least the third titanosaurian species to be erected based on material from this formation; the others are '*Antarctosaurus*' *giganteus*[20–22] and *Petrobrasaurus puestohernandezi*[23]. According to Garrido[19], the titanosaur *Muyelensaurus pecheni* also comes from the Plottier Formation, although this taxon was assigned to the Portezuelo Formation by its describers[24]. Furthermore, at least two additional titanosaurian specimens have been reported from the Plottier Formation, although these currently remain unidentified at the generic level: four anterior caudal vertebrae of an indeterminate aeolosaurine (MAU-Pv-N-414[25]) and UNCUYO-LD 313, a partial appendicular skeleton that includes, among other elements, the complete left pes[7]. *Notocolossus* is easily distinguished from *Muyelensaurus* and *Petrobrasaurus* in, for example, the morphology of the humerus, which is much more slender, especially proximally, in these two taxa than it is in the new form (see Calvo *et al.*[24]:fig. 12b; Filippi *et al.*[23]:fig. 6a). Unfortunately, however, comparisons with '*Antarctosaurus*' *giganteus* are more difficult. The holotype of '*A.*' *giganteus* (MLP 26-316) consists of rib fragments, two partial posterior caudal vertebrae, both incomplete pubes, both femora, a distal tibia, and other poorly preserved and indeterminate limb elements[20,22]; some recent authors (e.g., Upchurch *et al.*[26]) have regarded this taxon as a *nomen dubium*. Like *Notocolossus*, '*A.*' *giganteus* was undoubtedly very large: for instance, at 2310 mm, the left femur of MLP 26-316 is the longest complete limb bone that has been described for any titanosaur (see Paul[27]:table 1; Lacovara *et al.*[28]:table 1). Nevertheless, the only skeletal element preserved in common to *Notocolossus* and '*A.*' *giganteus* is the pubis, and it is highly incomplete in both taxa, precluding any meaningful comparisons between them.

VI. Comparison with *Mendozasaurus neguyelap*

Mendozasaurus neguyelap is the most completely known titanosaur from Mendoza Province[1,2]. Here, we compare skeletal elements of this taxon to overlapping bones in *Notocolossus gonzalezparejasi* to definitively differentiate these taxa. As noted above, although the localities that have produced fossils of *Mendozasaurus* and *Notocolossus* are close to one another—the former comes from south of Cerro Guillermo in southern-most Mendoza, the latter from Cerro Guillermo itself—these sites are stratigraphically separated: *Mendozasaurus* was recovered from the middle–upper Coniacian Sierra Barrosa Formation, whereas *Notocolossus* comes from the upper Coniacian–lower Santonian Plottier Formation.

Most elements preserved in *Notocolossus* are also represented in *Mendozasaurus*, facilitating comparisons between these taxa. The anterior dorsal vertebrae of the two forms differ in multiple respects. Although some of these differences could conceivably be due to positional variation, the single known anterior dorsal vertebra of *Notocolossus* and the best-preserved anterior dorsal of *Mendozasaurus* (IANIGLA-PV 066[1,2]) are thought to be closely comparable in serial position (the *Notocolossus* dorsal is here regarded as the second or third; the *Mendozasaurus* dorsal is considered to be the third[2]). The centrum of the anterior dorsal vertebra of *Notocolossus* has a proportionally more prominent, anteriorly-projecting articular condyle and relatively small lateral pneumatic fossae ('pleurocoels'). The centrum is much larger relative to the diameter of the neural canal than is the centrum of IANIGLA-PV 066. Overall, the *Notocolossus* neural arch is considerably lower and wider due to its longer transverse processes and shorter neural spine; moreover, the dorsal edge of the left transverse process meets the neural spine at an obtuse angle, whereas in *Mendozasaurus* the processes intersect the spine at nearly right angles. In IANIGLA-PV 066, the ventral edge of the intraprezygapophyseal lamina is linked to the dorsal margin of

the neural canal by a stout ‘medial pillar’[1], whereas in *Notocolossus* the intraprezygapophyseal lamina forms the dorsal margin of the neural canal. In *Mendozasaurus*, the base of the prespinal lamina is connected to the prezygapophyses by short spinoprezygapophyseal laminae; these latter laminae do not occur in the UNCUYO-LD 301 dorsal vertebra. Furthermore, the two ‘accessory’ laminae within the parapophyseal centrodiapophyseal fossa of *Notocolossus* are not present in *Mendozasaurus* (or, to our knowledge, any other titanosaur). Unfortunately, the posterior surface of the UNCUYO-LD 301 vertebra is currently obscured by a protective ‘cradle’. As such, we are unable to determine whether the two hypothesized autapomorphies evident on the posterior aspect of the anterior dorsal neural arch of *Mendozasaurus*[1] ([1] subtriangular centropostzygapophyseal [= ‘infrapostzygapophyseal’] fossae and [2] dorsolaterally–ventromedially-oriented ‘postzygapostspinal’ laminae connecting the postzygapophyses to the base of the postspinal lamina) are also present in *Notocolossus*.

The anterior caudal vertebrae of *Mendozasaurus* and *Notocolossus* may be easily distinguished as well. As with the dorsal vertebrae, a few of their differences may be attributable to serial variation; however, because one of the best preserved anterior caudals of *Mendozasaurus* (IANIGLA-PV 065/1, regarded as the first or second in the holotypic series[1]) is thought to be close in position to the most complete caudal of *Notocolossus* (that of the holotype, UNCUYO-LD 301, here considered the third or fourth), most of these distinctions are probably taxonomic in nature. The centrum of IANIGLA-PV 065/1 is much shorter anteroposteriorly than is that of the caudal of UNCUYO-LD 301; this could, however, be due to taphonomic deformation, especially since another anterior caudal vertebra of the *Mendozasaurus* holotype (IANIGLA-PV 065/4) has a much longer centrum[1] that is more comparable to those of *Notocolossus* (both specimens, UNCUYO-LD 301 and 302) in this regard. Perhaps more significantly, the transverse processes of IANIGLA-PV 065/1 are substantially shorter than are those of the anterior caudals of UNCUYO-LD 301 and 302, despite the fact that this *Mendozasaurus* vertebra is thought to have been situated more anteriorly in the series than are all known caudals of *Notocolossus*. Because, in titanosaurs represented by complete anterior–middle caudal series (e.g., *Alamosaurus*[29], *Baurutitan*[30], *Dreadnoughtus*[28], *Epachthosaurus*[31]), the transverse processes gradually decrease in length as one moves posteriorly through the series, we regard the considerably longer anterior caudal transverse processes of *Notocolossus* as a well-supported distinction between this taxon and *Mendozasaurus*.

Several differences are evident between the anterior caudal neural arches of IANIGLA-PV 065 and UNCUYO-LD 301 as well. In *Notocolossus*, there is no evidence of the dorsal prominences of the prezygapophyses or the deep ‘interzygapophyseal fossa’ (the confluent postzygapophyseal spinodiapophyseal/postzygapophyseal centrodiapophyseal fossa of Wilson *et al.*[32]) that characterize *Mendozasaurus* (e.g., IANIGLA-PV 065/4). Also, whereas *Mendozasaurus* has elongate, well-developed spinoprezygapophyseal and prespinal laminae, in *Notocolossus*, there is no clear evidence of the prespinal lamina, and the spinoprezygapophyseal laminae are short. The anterolateral margins of the neural spine of the new taxon are instead comprised by seemingly novel laminae that converge ventrally and merge immediately dorsal to the prezygapophyses, forming a ‘V-shape’ in anterior view. The haemal arches of *Notocolossus* (UNCUYO-LD 302) are too incomplete for meaningful comparisons with those of *Mendozasaurus*.

The humeri of *Notocolossus* (UNCUYO-LD 301) and *Mendozasaurus* (IANIGLA-PV 069) differ dramatically, especially at their proximal ends. The mediolateral width of the proximal end of the *Notocolossus* humerus is 2.88 times the width at midshaft, substantially greater than in any

other titanosaur; in *Mendozasaurus*, by contrast, this ratio is only 2.41 (Table 1). The medial half of the proximal margin of the *Notocolossus* humerus is strongly projected proximomedially, whereas in *Mendozasaurus* this same margin is horizontal. *Notocolossus* also bears a small proximolateral process (= 'supracoracoideus tuberosity') that is absent in *Mendozasaurus*.

Finally, the complete, articulated pes of the referred specimen of *Notocolossus* (UNCUYO-LD 302) differs from the known pedal material of *Mendozasaurus* as well. Compared to that of metatarsal I, the proximodistal length of metatarsal III is shorter in *Notocolossus* than in any other titanosaurian taxon, including *Mendozasaurus*, though it is perhaps notable that a probably associated metatarsus of the latter (IANIGLA-PV 077) comes the closest in this regard (Table 2). *Notocolossus* also exhibits a substantially lower metatarsal IV:metatarsal I length ratio (1.33) than does *Mendozasaurus* (1.46) (Table 2). Metatarsal V is only minimally distally expanded in *Mendozasaurus*, as is the case in most other sauropods, but not in *Notocolossus*. The two preserved pedal unguals of *Mendozasaurus* differ from those of UNCUYO-LD 302 in being proximodistally elongate and dorsoventrally tapered distally, as is typical of sauropods, instead of short and distally truncated as they are in *Notocolossus*.

VII. Estimates of body dimensions

The incompleteness of known specimens of *Notocolossus*, coupled with the variability in body proportions observed within Titanosauria (e.g., long-necked taxa such as *Futalognkosaurus*[33] versus relatively short-necked forms such as *Mendozasaurus*[1,2]), renders estimation of the body dimensions of the new taxon problematic. Nevertheless, we estimated the proximodistal lengths of the femora of the two known *Notocolossus* specimens (UNCUYO-LD 301 and 302) and the body mass of the holotype (UNCUYO-LD 301) using methods closely comparable to those employed by Smith *et al.*[34] and Lamanna[35] to estimate these same dimensions in the type specimen of the giant Egyptian titanosaur *Paralititan* (CGM 81119).

To estimate the femoral length of the *Notocolossus* holotype, we first compiled humeral and femoral lengths of articulated or definitively associated titanosaurian skeletons that preserve both of these elements in their entirety, then transformed these data into logarithms (base 10) (see Supplementary Table S4). We then plotted log humeral versus log femoral length and performed a linear regression to produce an allometric equation relating these dimensions, allowing one to be estimated from the other (Supplementary Fig. S9). The humeral length of UNCUYO-LD 301 (1760 mm) was then 'plugged in' to this equation, generating an estimated femoral length of 2166 mm for this specimen (Supplementary Table S4). By comparison, the maximum femoral lengths of the type specimens of the giant titanosaurs *Dreadnoughtus* (MPM-PV 1156)[28], *Futalognkosaurus* (MUCPv-323)[36], and '*Antarctosaurus*' *giganteus* (MLP 26-316)[20] are 1910, 1980, and 2310 mm, respectively, and the estimated femoral length of the holotype of *Paralititan* (CGM 81119) is 2083 mm (Supplementary Table S4). Identical methods applied to the proximodistal length of the femur versus that of metatarsal III and used in concert with the metatarsal III length of UNCUYO-LD 302 (197 mm) yielded an estimated femoral length of 1283 mm for this referred specimen of *Notocolossus* (Supplementary Fig. S10; Supplementary Table S5). The length of metatarsal III of the ?*Alamosaurus* specimen NMMNH P-49967 (270 mm) produced an estimated femoral length of 1632 mm, lower than the 1.7–2.1 m length estimated by D'Emic *et al.*[37].

Having demonstrated that, based on estimated femoral length, the holotype of *Notocolossus* represents a considerably larger individual than does the referred specimen, we then estimated the body mass of the former. Again, we used methods comparable to those employed by Lamanna[35]. We compiled minimum midshaft humeral and femoral circumferences of titanosaurian skeletons that preserve both of these dimensions, then transformed these data into logarithms (base 10) (Supplementary Table S6). We plotted log humeral versus log femoral midshaft circumference and performed a linear regression to produce an allometric equation relating these dimensions, allowing one to be estimated based on the other (Supplementary Fig. S11). The humeral circumference of UNCUYO-LD 301 (770 mm) was then ‘plugged in’ to this equation, generating an estimated femoral circumference of 936 mm for this specimen (Supplementary Table S6). We then used a scaling equation recently proposed by Campione and Evans[38] ($\log BM = 2.749 * \log C_{H+F} – 1.104$, where BM is body mass and C_{H+F} is combined humeral and femoral circumference) to estimate the mass of UNCUYO-LD 301 at 60,398 kg (~60.4 metric tons; the mean percent prediction error of this equation is 25.6%[38], which yields a lower estimate of 44,936 kg and an upper estimate of 75,860 kg for this same specimen). The same equation generates mean estimates of 38,091, 39,513, 49,986, 59,291, and 96,430 kg for the holotypic specimens of *Futalognkosaurus*, ‘*A.*’ *giganteus*, *Paralititan*, and *Dreadnoughtus*, and an isolated femur tentatively referred to *Argentinosaurus*, respectively (Supplementary Table S6). Although the applicability of this scaling equation to unusually large titanosaurs has recently been challenged[39], it may still provide insight into the body masses of these dinosaurs relative to one another. As such, although (due to the incomplete nature of the specimen) this must be regarded as tentative, it appears that the holotype of *Notocolossus* may represent one of the most massive titanosaurian individuals—and terrestrial animals—that has been discovered to date.

VIII. Photogrammetric models

To better document the skeletal morphology of *Notocolossus*, we herein provide three-dimensional digital models of the anterior dorsal vertebra and right humerus of the holotype (UNCUYO-LD 301) and a cast of the complete and articulated right tarsus and pes of the referred specimen (UNCUYO-LD 302). These models were kindly produced by Stephen Poropat of the Australian Age of Dinosaurs Natural History Museum (Winton, Queensland, Australia). Poropat photographed each of these specimens from all feasible perspectives using a Nikon D90 digital camera fitted with a VR 18–105 mm lens, then used Agisoft PhotoScan Professional Edition software to assemble the resulting photos into photogrammetric digital models. He then used this software to convert each model into a three-dimensional (3D) Adobe Portable Document Format (.pdf) file (see Supplementary Figs. S1, S3, and S4). (Viewing and navigating Adobe 3D .pdf files requires Adobe Acrobat or Acrobat Reader. The latter is freely available for download at http://get.adobe.com/reader/.) Users may download and rotate each model into whatever orientation they prefer, zoom in on particular osteological structures, etc. (Note, however, that the posterior surface of the dorsal vertebra and the posterodistal surface of the humerus are obscured by supportive ‘cradles’; as such, these areas are not represented in the digital models.) Surface files (.obj, .stl) of all models are available to qualified researchers upon request to the senior author (B.J.G.R.).

IX. Phylogenetic character list

The following 350 morphological characters were employed in our phylogenetic analysis and are listed by general anatomical region. The vast majority (340) of these characters (numbers 1–130, 134–256, 259–330, 332, 333, and 335–347) were drawn directly from Carballido and Sander[40] and references cited therein. (Their respective character numbers in that analysis are as follows: 1–130, 132–254, 255–326, 327, 328, and 329–341.) Except for minor editing (e.g., correction of typographical errors), the descriptions of these 340 characters are as presented by Carballido and Sander[40]; also, the literature attributions of a few characters (103, 135, 149, 213, 245, and 249) have been corrected. Furthermore, character 133 (character 131 of Carballido and Sander[40]) was slightly modified from that study. A significant aspect of the current analysis is the addition of nine characters that are presented in *italics* below (numbers 131, 132, 257, 258, 331, 334, and 348–350). Of these, characters 131 and 132 were taken from González Riga and Ortiz David[4] and sources in that paper, character 257 was modified from Mannion *et al.*[41], character 258 was modified from Curry Rogers[42], and character 350 was modified from Upchurch[43]; the remaining four characters (331, 334, 348, and 349) are newly formulated herein. As in Carballido and Sander[40], 24 characters (12, 58, 95, 96, 102, 106, 108, 115, 116, 119, 120, 156, 166, 215, 218, 234–237, 260, 271, 302, 303, and 305) were treated as ordered. The three characters that pertain to pedal phalangeal reduction (numbers 348–350) were unordered.

Craniodental characters

1) Posterolateral processes of premaxilla and lateral processes of maxilla, shape: without midline contact (0); with midline contact forming marked narial depression, subnarial foramen not visible laterally (1). (Wilson[44]:ch. 1)

2) Premaxillary anterior margin shape: without step (0); with marked but short step (1); with marked and long step (2). (modified from Wilson[44]:ch. 2 by Carballido and Sander[40]:ch. 2)

3) Premaxilla, shape of ascending process in lateral view: convex (0); concave, with a large dorsal projection (1); sub-rectilinear and directed posterodorsally (2). (Whitlock[45]:ch. 3)

4) Premaxilla, external surface: without anteroventrally-orientated vascular grooves originating from an opening in the maxillary contact (0); vascular grooves present (1). (Whitlock[45]:ch. 2)

5) Maxillary border of external naris, length: short, making up much less than one-fourth narial perimeter (0); long, making up more than one-third narial perimeter (1). (Wilson[44]:ch. 3)

6) Maxilla, foramen anterior to preantorbital fenestra: absent (0); present (1). (Zaher *et al.*[46]:ch. 244)

7) Preantorbital fenestra: absent (0); present, being wide and laterally open (1). (modified from Wilson[44]:ch. 4 by Carballido and Sander[40]:ch. 7)

8) Subnarial foramen and exterior maxillary foramen, position: well distant from one another (0); separated by narrow bony isthmus (1). (Wilson[44]:ch. 5)

9) Antorbital fenestra: much shorter than (less than 85% of) orbital maximum diameter (0); subequal to (greater than 85% of) orbital maximum diameter (1). (modified from Wilson[44]:ch. 6 by Whitlock[45]:ch. 13)

10) Antorbital fenestra, shape of dorsal margin: straight or convex (0); concave (1). (Whitlock[45]:ch. 14)

11) Antorbital fossa: present (0); absent (1). (Wilson[44]:ch. 7)

12) External nares position: terminal (0); retracted to level of orbit (1); retracted to a position between orbits (2). (Wilson[44]:ch. 8)

13) External nares, maximum diameter: shorter (0); or longer than orbital maximum diameter (1). (Wilson[44]:ch. 9)

14) Orbital ventral margin, anteroposterior length: broad, with subcircular orbital margin (0); reduced, with acute orbital margin (1). (Wilson[44]:ch. 10)

15) Lacrimal, anterior process: present (0); absent (1). (Wilson[44]:ch. 11)

16) Jugal contribution to the ventral border of the skull: present (0); absent (1). (Carballido *et al.*[47]:ch. 16)

17) Quadratojugal–maxilla contact: absent or small (0); broad (1). (Whitlock[45]:ch. 10)

18) Jugal–ectopterygoid contact: present (0); absent (1). (Wilson[44]:ch. 12)

19) Jugal, contribution to antorbital fenestra: very reduced or absent (0); large, bordering approximately one-third its perimeter (1). (Wilson[44]:ch. 13)

20) Quadratojugal, position of anterior terminus: posterior to middle of orbit (0); anterior margin of orbit or beyond (1). (Whitlock[45]:ch. 30)

21) Quadratojugal, anterior process length: short, anterior process shorter than dorsal process (0); long, anterior process more than twice as long as dorsal process (1). (Wilson[44]:ch. 32)

22) Quadratojugal, angle between anterior and dorsal processes: less than or equal to 90°, so that the quadrate shaft is directed dorsally (0); greater than 90°, approaching 130°, so that the quadrate shaft slants posterodorsally (1). (Whitlock[45]:ch. 31)

23) Ventral edge of anterior surface of quadratojugal: straight, not expanded ventrally (0); concave due to a ventral expansion of the anterior region (1). (Upchurch *et al.*[26]:ch. 26)

24) Squamosal contribution to supratemporal fenestra: present, squamosal well visible in dorsal view (0); reduced or absent (1). (Curry Rogers[42]:ch. 37)

25) Squamosal–quadratojugal contact: present (0); absent (1). (Wilson[44]:ch. 31)

26) Squamosal, posteroventral margin: smooth (0); with prominent, ventrally directed ‘prong’ (1). (Whitlock[45]:ch. 37)

27) Prefrontal posterior process size: small, not projecting far posterior of frontal–nasal suture (0); elongate, approaching parietal (1). (Wilson[44]:ch. 14)

28) Prefrontal, posterior process shape: flat (0); hooked (1). (Wilson[44]:ch. 15)

29) Prefrontal, anterior process: absent (0); present (1). (Curry Rogers[42]:ch. 30)

30) Prefrontal–frontal contact width: large, equal to or longer than the anteroposterior length of the prefrontal (0); narrow, less than half the anteroposterior length of the prefrontal (1). (Zaher *et al.*[46]:ch. 239)

31) Postorbital, ventral process shape: transversely narrow (0); broader transversely than anteroposteriorly (1). (Wilson[44]:ch. 16)

32) Postorbital, posterior process: present (0); absent (1). (Wilson[44]:ch. 17)

33) Postorbital, posterior margin articulating with squamosal: with tapering posterior process (0); with deep posterior process (1). (Zaher *et al.*[46]:ch. 245)

34) Frontal contribution to supratemporal fossa: present (0); absent (1). (Wilson[44]:ch. 18)

35) Frontals, midline contact (symphysis) in adult individuals: sutured (0); fused (1). (Wilson[44]:ch. 19)

36) Frontal, anteroposterior length: approximately twice (0); or less than minimum transverse breadth (1). (Wilson[44]:ch. 20)

37) Frontal–nasal suture, shape: flat or slightly bowed anteriorly (0); V-shaped, pointing posteriorly (1). (Whitlock[45]:ch. 21)

38) Frontals, dorsal surface: without paired grooves facing anterodorsally (0); grooves present, extend onto nasal (1). (Whitlock[45]:ch. 22)

39) Frontal, contribution to dorsal margin of orbit: less than 1.5 times contribution of prefrontal (0); at least 1.5 times contribution of prefrontal (1). (Whitlock[45]:ch. 23)

40) Parietal occipital process, dorsoventral height: short, less than the diameter of the foramen magnum (0); deep, nearly twice the diameter of the foramen magnum (1). (Wilson[44]:ch. 21)

41) Parietal, contribution to post-temporal fenestra: present (0); absent (1). (Wilson[44]:ch. 22)

42) Parietal, distance separating supratemporal fenestrae: less than (0); or twice the long axis of supratemporal fenestra (1). (Wilson[44]:ch. 24)

43) Postparietal foramen: absent (0); present (1). (Wilson[44]:ch. 23)

44) Paroccipital process distal terminus: straight, slightly expanded surface (0); rounded, tongue-like process (1). (Whitlock[45]:ch. 42)

45) Supratemporal fenestra: present (0); absent (1). (Wilson[44]:ch. 25)

46) Supratemporal fenestra, long axis orientation: anteroposterior (0); transverse (1). (Wilson[44]:ch. 26)

47) Supratemporal fenestra, maximum diameter: much longer than (0); or subequal to that of foramen magnum (1). (Wilson[44]:ch. 27)

48) Supratemporal region, anteroposterior length: temporal bar longer (0); or shorter anteroposteriorly than transversely (1). (Wilson[44]:ch. 28)

49) Supratemporal fossa, lateral exposure: not visible laterally, obscured by temporal bar (0); visible laterally, temporal bar shifted ventrally (1). (Wilson[44]:ch. 29)

50) Supraoccipital, sagittal nuchal crest: broad, weakly developed (0); narrow, sharp and distinct (1). (Whitlock[45]:ch. 45)

51) Laterotemporal fenestra, anterior extension: posterior to orbit (0); ventral to orbit (1). (Wilson[44]:ch. 30)

52) Quadrate fossa: absent (0); present (1). (Wilson[44]:ch. 33)

53) Quadrate fossa, depth: shallow (0); deeply invaginated (1). (Wilson[44]:ch. 34)

54) Quadrate fossa, orientation: posterior (0); posterolateral (1). (Wilson[44]:ch. 35)

55) Quadrate, articular surface shape: quadrangular in ventral view, oriented transversely (0); roughly triangular in shape or thin, crescent-shaped surface with anteriorly-directed medial process (1). (modified *sensu* Mannion *et al.*[48] from Whitlock[45]:ch. 32 by Carballido and Sander[29]:ch. 55)

56) Quadrate, articular surface shape: quadrangular in ventral view, oriented transversely or roughly triangular in shape (0); thin, crescent-shaped surface with anteriorly directed medial process (1). (modified *sensu* Mannion *et al.*[48] from Whitlock[45]:ch. 32 by Carballido and Sander[40]:ch. 56)

57) Palatobasal contact, shape: pterygoid with small facet (0); dorsomedially-orientated hook (1); or rocker-like surface for basipterygoid articulation (2). (Wilson[44]:ch. 36)

58) Pterygoid, transverse flange (i.e., ectopterygoid process) position: posterior to orbit (0); between orbit and antorbital fenestra (1); anterior to antorbital fenestra (2). (Wilson[44]:ch. 37)

59) Pterygoid, quadrate flange size: large, palatobasal and quadrate articulations well separated (0); small, palatobasal and quadrate articulations approach (1). (Wilson[44]:ch. 38)

60) Pterygoid, palatine ramus shape: straight, at level of dorsal margin of quadrate ramus (0); stepped, raised above level of quadrate ramus (1). (Wilson[44]:ch. 39)

61) Pterygoid, sutural contact with ectopterygoid: broad, along medial or lateral surface (0); narrow, restricted to anterior tip of ectopterygoid (1). (Zaher *et al.*[46]:ch. 240)

62) Palatine, lateral ramus shape: plate-shaped (long maxillary contact) (0); rod-shaped (narrow maxillary contact) (1). (Wilson[44]:ch. 40)

63) Epipterygoid: present (0); absent (1). (Wilson[44]:ch. 41)

64) Vomer, anterior articulation: maxilla (0); premaxilla (1). (Wilson[44]:ch. 42)

65) Supraoccipital, height: twice (0); subequal to or less than height of foramen magnum (1). (Wilson[44]:ch. 43)

66) Paroccipital process, ventral non-articular process: absent (0); present (1). (Wilson[44]:ch. 44)

67) Crista prootica, size: rudimentary (0); expanded laterally into dorsolateral process (1). (Wilson[44]:ch. 45)

68) Basipterygoid processes, length: short, approximately twice (0); or elongate, at least four times basal diameter (1). (Wilson[44]:ch. 46)

69) Basipterygoid processes, angle of divergence: approximately 45° (0); less than 30° (1). (Wilson[44]:ch. 47)

70) Basal tubera, anteroposterior depth: approximately half dorsoventral height (0); sheet-like, 20% dorsoventral height (1). (Wilson[44]:ch. 48)

71) Basal tubera, breadth: much broader than (0); or narrower than occipital condyle (1). (Wilson[44]:ch. 49)

72) Basal tubera: distinct from basipterygoid (0); reduced to slight swelling on ventral surface of basipterygoid (1). (Whitlock[45]:ch. 53)

73) Basal tubera, shape of posterior face: convex (0); slightly concave (1). (Whitlock[45]:ch. 54)

74) Basioccipital depression between foramen magnum and basal tubera: absent (0); present (1). (Wilson[44]:ch. 50)

75) Basisphenoid/basipterygoid recess: present (0); absent (1). (Wilson[44]:ch. 51)

76) Basisphenoid/quadrate contact: absent (0); present (1). (Wilson[44]:ch. 52)

77) Basisphenoid, sagittal ridge between basipterygoid processes: absent (0); present (1). (Zaher *et al.*[46]:ch. 242)

78) Basipterygoid processes orientation: perpendicular to (0); or angled approximately 45° to skull roof (1). (Wilson[44]:ch. 53)

79) Basipterygoid, area between basipterygoid processes and parasphenoid rostrum: is a mildly concave subtriangular region (0); forms a deep, slot-like cavity that passes posteriorly between the bases of the basipterygoid processes (1). (Mannion *et al.*[48]:ch. 48)

80) Occipital region of skull, shape: anteroposteriorly deep, paroccipital processes oriented posterolaterally (0); flat, paroccipital processes oriented transversely (1). (Wilson[44]:ch. 54)

81) Dentary, depth of anterior end of ramus: slightly less than that of dentary at midlength (0); 150% minimum depth (1). (Wilson[44]:ch. 55)

82) Dentary, anteroventral margin shape: gently rounded (0); sharply projecting triangular process (1). (Wilson[44]:ch. 56)

83) Dentary symphysis, orientation: angled 15° or more anteriorly to (0); or perpendicular to axis of jaw ramus (1). (Wilson[44]:ch. 57)

84) Dentary, cross-sectional shape of symphysis: oblong or rectangular (0); subtriangular, tapering sharply towards ventral extreme (1); subcircular (2). (Whitlock[45]:ch. 60)

85) Dentary, tuberosity on labial surface near symphysis: absent (0); present (1). (Whitlock[45]:ch. 57)

86) Mandible, coronoid eminence: strongly expressed, clearly rising above plane of dentigerous portion (0); absent (1). (Whitlock[45]:ch. 62)

87) External mandibular fenestra: present (0); absent (1). (Wilson[44]:ch. 58)

88) Surangular depth: less than twice (0); or more than 2.5 times maximum depth of angular (1). (Wilson[44]:ch. 59)

89) Surangular ridge separating adductor and articular fossae: absent (0); present (1). (Wilson[44]:ch. 60)

90) Adductor fossa, medial wall depth: shallow (0); deep, prearticular expanded dorsoventrally (1). (Wilson[44]:ch. 61)

91) Splenial posterior process, position: overlapping angular (0); separating anterior portions of prearticular and angular (1). (Wilson[44]:ch. 62)

92) Splenial posterodorsal process: present, approaching margin of adductor chamber (0); absent (1). (Wilson[44]:ch. 63)

93) Coronoid, size: extending to dorsal margin of jaw (0); reduced, not extending dorsal to splenial (1); absent (2). (Wilson[44]:ch. 64)

94) Tooth rows, shape of anterior portions: narrowly arched, anterior portion of tooth rows V-shaped (0); broadly arched, anterior portion of tooth rows U-shaped (1); rectangular, tooth-bearing portion of jaw perpendicular to jaw rami (2). (Wilson[44]:ch. 65)

95) Tooth rows, length: extending to orbit (0); restricted anterior to orbit (1); restricted anterior to antorbital fenestra (2); restricted anterior to subnarial foramen (3). (modified from Wilson[44]:ch. 66 by Carballido and Sander[40]:ch. 95)

96) Dentary teeth, number: greater than 20 (0); 10–17 (1); 9 or fewer (2). (modified from Wilson[44]:ch. 73 by Carballido and Sander[40]:ch. 96)

97) Replacement teeth per alveolus, number: two or fewer (0); more than four (1). (Wilson[44]:ch. 74)

98) Lateral plate: absent (0); present (1). (Upchurch *et al.*[26]:ch. 9)

99) Teeth, orientation: perpendicular (0); or oriented anteriorly relative to jaw margin (1). (Wilson[44]:ch. 75)

100) Tooth crowns, orientation: aligned along jaw axis, crowns do not overlap (0); aligned slightly anterolingually, tooth crowns overlap (1). (Wilson[44]:ch. 69)

101) Crown-to-crown occlusion: absent (0); present (1). (Wilson[44]:ch. 67)

102) Occlusal pattern: interlocking, V-shaped facets (0); high-angled planar facets (1); low-angled planar facets (2). (Wilson[44]:ch. 68)

103) Tooth crowns, cross-sectional shape at mid-crown: elliptical (0); D-shaped (1); sub-cylindrical (2); cylindrical (3). (modified from Calvo[49] by Wilson[44]:ch. 70)

104) Enamel surface texture: smooth (0); wrinkled (1). (Wilson[44]:ch.71)

105) Thickness of enamel asymmetric labiolingually: absent (0); present (1). (Whitlock[45]:ch. 74)

106) Marginal tooth denticles: present (0); absent on posterior edge (1); absent on both anterior and posterior edges (2). (Wilson[44]:ch. 72)

107) Teeth, longitudinal grooves on lingual aspect: absent (0); present (1). (Wilson[44]:ch. 76)

108) SI values for tooth crowns: less than 3.0 (0); 3.0–4.0 (1); 4.0–5.0 (2); more than 5.0 (3). (Upchurch *et al.*[26]:chs. 67–69)

Cervical vertebral characters

109) Cervical vertebrae, number: 10 or fewer (0); 12 (1); 13–14 (2); 15 (3); 16 or more (4). (modified from Wilson[44]:ch. 80; Upchurch *et al.*[26]:chs. 96–100 by Carballido and Sander[40]:ch. 109)

110) Atlas, intercentrum occipital facet shape: rectangular in lateral view, length of dorsal aspect subequal to that of ventral aspect (0); expanded anteroventrally in lateral view, anteroposterior length of dorsal aspect shorter than that of ventral aspect (1). (Wilson[44]:ch. 79)

111) Cervical centra, articulations: amphicoelous (0); opisthocoelous (1). (Salgado *et al.*[50]:ch. 1; Upchurch[43]:ch. 81; Wilson[44]:ch. 82; Upchurch *et al.*[26]:ch. 103)

112) Cervical centra, ventral surface: flat or slightly convex transversely (0); transversely concave (1). (Upchurch[43]:ch. 84; Upchurch *et al.*[26]:ch. 107)

113) Cervical centra, midline keels on ventral surface: prominent and plate-like (0); reduced to low ridges or absent (1). (Upchurch[43]:ch. 83; Upchurch *et al.*[26]:ch. 106)

114) Cervical centra, lateral pneumatic fossae (‘pleurocoels’): absent (0); present with well-defined anterior, dorsal, and ventral edges, but posterior edge is poorly defined (1); present with all edges well-defined (2); present but very reduced in size (3). (Carballido *et al.*[47]:ch. 114)

115) Cervical centra, ‘pleurocoels’: single, without divisions (0); with well-defined anterior excavation and smooth posterior fossa (1); divided by bony septum, resulting in anterior and posterior lateral excavations (2); divided into three or more lateral excavations, resulting in a complex morphology (3). (modified from Salgado *et al.*[50]:ch. 8; Wilson[44]:chs. 78, 83; Harris[51]:ch. 108 by Carballido and Sander[40]:ch. 115)

116) Cervical vertebrae, height divided by width (measured at posterior articular surface): greater than 1.1 (0); approximately 1.0 (1); between 0.9 and 0.7 (2); less than 0.7 (3). (modified from Upchurch[43]:ch. 85; Wilson[44]:ch. 84; Upchurch *et al.*[26]:ch. 108 by Carballido and Sander[40]:ch. 116)

117) Cervical centra, small notch in dorsal margin of posterior articular surface: absent (0); present (1). (Carballido *et al.*[47]:ch. 117)

118) Cervical vertebrae, neural arch lamination: well developed, with well-marked laminae and fossae (0); rudimentary, with diapophyseal laminae absent or very slightly marked (1). (Wilson[44]:ch. 81)

119) Cervical vertebrae with accessory lamina that extends from postzygodiapophyseal lamina to spinoprezygapophyseal lamina: absent (0); present (1). (modified from Sereno *et al.*[52]:chs. 50, 51; Whitlock[45]:chs. 78, 96 by Carballido and Sander[40]:ch. 119)

120) Cervical centra, internal pneumaticity: absent (0); present, with single and wide cavities (1); present, with several small and complex internal cavities (2). (modified from Carballido *et al.*[53]:ch. 100 by Carballido and Sander[40]:ch. 120)

121) Anterior cervical vertebrae, prespinal lamina: absent (0); present (1). (Carballido *et al.*[47]:ch. 121)

122) Anterior cervical vertebrae, neural spine shape: single (0); bifid (1). (Wilson[44]:ch. 72; Upchurch *et al.*[26]:ch. 118)

123) Middle and posterior cervical vertebrae, prespinal lamina: absent (0); present (1). (Carballido *et al.*[47]:ch. 123)

124) Middle cervical vertebrae, lateral fossae on prezygapophyseal process: absent (0); present (1). (Harris[51])

125) Middle cervical vertebrae, height of neural arch: less than height of posterior articular surface (0); greater than height of posterior articular surface (1). (Wilson[44]:ch. 87; comparable to Upchurch *et al.*[26]:chs. 111–112)

126) Middle cervical centra, anteroposterior length divided by height of posterior articular surface: less than 4 (0); more than 4 (1). (Wilson[44]:ch. 74; Upchurch *et al.*[26]:ch. 102)

127) Middle and posterior cervical vertebrae, morphology of centroprezygapophyseal lamina: single (0); dorsally divided, resulting in lateral and medial laminae, with the medial lamina connected to the intraprezygapophyseal lamina and not the prezygapophysis (1); divided, resulting in the presence of a 'true' divided centroprezygapophyseal lamina, both rami of which are dorsally connected to the prezygapophysis (2). (Carballido *et al.*[47]:ch. 127)

128) Middle and posterior cervical vertebrae, morphology of centropostzygapophyseal lamina: single (0); divided, with medial part contacting intrapostzygapophyseal lamina (1) (Carballido *et al.*[47]:ch. 128)

129) Middle and posterior cervical vertebrae, articular surface of zygapophyses: flat (0); transversely convex (1). (Upchurch *et al.*[26]:ch. 114)

130) Posterior cervical vertebrae, lateral profile of neural spine: displays steeply sloping anterior and posterior faces (0); displays steeply sloping anterior face and noticeably less steep posterior margin (1). (Upchurch *et al.*[26]:ch. 119)

131) Spinodiapophyseal ('supradiapophyseal') fossa in cervical vertebrae, absent (0); shallow or reduced (1); deep and extended (2). (González Riga[2]; González Riga and Ortiz David[4]:ch. 32)

132) Lateral expansions of cervical neural spines originating from lateral laminae: absent (0); present (1). (González Riga and Ortiz David[4]:ch. 27)

133) Posterior cervical neural spines, shape: narrow (0) or laterally expanded (1). (modified from González Riga *et al.*[5]:ch. 30 by this paper)

134) Posterior cervical and anterior dorsal vertebrae, neural spine shape: single (0); bifid (1). (Wilson[44]:ch. 89; Upchurch *et al.*[26]:ch. 118)

135) Posterior cervical and anterior dorsal bifid neural spines, median tubercle: absent (0); present (1). (Wilson[44]:ch. 90)

Dorsal vertebral characters

136) Number of dorsal vertebrae: 14 or more (0); 13 (1); 12 (2); 10 (3). (modified from Wilson[44]:ch. 91; Upchurch *et al.*[26]:chs. 122–125 by Carballido and Sander[40]:ch. 134)

137) Dorsal centra, 'pleurocoels': absent (0); present (1). (Wilson[44]:ch. 78; Upchurch *et al.*[26]:ch. 128)

138) Dorsal vertebrae, transverse processes: directed laterally or slightly dorsally (0); directed strongly dorsolaterally (1). (Upchurch *et al.*[26]:ch. 138)

139) Dorsal vertebrae, distal end of transverse process: curves smoothly into the dorsal surface of the process (0); is set off from the dorsal surface, the latter having a distinct, dorsally facing flattened area (1). (Upchurch *et al.*[26]:ch. 140)

140) Dorsal vertebrae, non-bifid neural spines in anterior or posterior view: possess subparallel lateral margins (0); possess lateral margins which slightly diverge dorsally (1); possess lateral margins which strongly diverge dorsally (2). (modified from Wilson[44]:ch. 107; Upchurch *et al.*[26]:ch. 155 by Carballido and Sander[40]:ch. 138)

141) Dorsal centra, pneumatic structures: absent, dorsal centra with solid internal structure (0); present, dorsal centra with large and simple air spaces (1); present, dorsal centra with small and complex air spaces (2). (modified from Carballido *et al.*[53]:ch. 100 by Carballido and Sander[40]:ch. 139)

142) Anterior and middle dorsal neural spines, spinoprezygapophyseal lamina: absent (0); present (1). (modified from Upchurch *et al.*[54]:ch. 131 by Carballido and Sander[40]:ch. 140)

143) Posterior dorsal neural spines, spinoprezygapophyseal lamina: absent (0); present (1). (modified from Upchurch *et al.*[54]:ch. 132 by Carballido and Sander[40]:ch. 141)

144) Dorsal vertebrae, single (non-bifid) neural spines, single prespinal lamina: absent (0); present (1). (modified from Salgado *et al.*[50]:ch. 14 by Carballido and Sander[40]:ch. 142)

145) Dorsal vertebrae, single (non-bifid) neural spines, single prespinal lamina: rough and wide, present in the dorsalmost part of the neural spine (0); rough and wide, extended through almost all the neural spine (1); smooth and narrow (2). (Carballido *et al.*[47]:ch. 143)

146) Dorsal vertebrae with single neural spines, middle single fossa projected through the midline of the neural spine: present (0); absent (1). (Carballido *et al.*[47]:ch. 144)

147) Dorsal vertebrae with single neural spines, middle single fossa projected through the midline of the neural spine: relatively wide median simple fossa (0); thin median simple fossa (1); extremely reduced median simple fossa (2). (Carballido *et al.*[47]:ch. 145)

148) Anterior dorsal centra, articular face shape: amphicoelous (0); opisthocoelous (1). (Wilson[44]:ch. 94; Upchurch *et al.*[26]:ch. 104)

149) Anterior and middle dorsal centra, 'pleurocoels': have rounded posterior margins (0); have tapering, acute posterior margins (1). (modified from Salgado *et al.*[50]:ch. 20; Upchurch[43]:ch. 96; Upchurch *et al.*[26]:ch. 127 by Carballido and Sander[40]:ch. 147)

150) Middle dorsal neural arches in lateral view, anterior edge of neural spines: project anterior to diapophysis (0); converge with diapophysis (1); project posterior to diapophysis (2). (Carballido *et al.*[47]:ch. 148)

151) Anterior and middle dorsal vertebrae, angle of zygapophyseal articulations: horizontal or slightly posteroventrally oriented (0); somewhat posteroventrally oriented (around 30º) (1); strongly posteroventrally oriented (more than 40º) (2). (Carballido *et al.*[47]:ch. 149)

152) Middle and posterior dorsal centra, ventral surface: convex transversely (0); flattened (1); slightly concave, sometimes with one or two crests (2). (modified from Upchurch *et al.*[26]:ch. 126 by Carballido and Sander[40]:ch. 150)

153) Middle dorsal vertebrae, hyposphene–hypantrum system: present (0); absent (1). (modified from Salgado *et al.*[50]:ch. 25; Wilson[44]:ch. 106; Upchurch *et al.*[26]:ch. 145 by Carballido and Sander[40]:ch. 151)

154) Posterior dorsal vertebrae, hyposphene–hypantrum system: present and well developed, usually with rhomboid shape (0); present and weakly developed, mainly as a laminar articulation (1); absent or only present in posteriormost dorsal vertebrae (2). (Carballido *et al.*[47]:ch. 152)

155) Middle and posterior dorsal vertebrae, transverse process length: short (0); long, at least 1.5 of the articular width of the centrum (1). (Carballido *et al.*[47]:ch. 153)

156) Middle and posterior dorsal vertebrae with a single lamina (the single intrapostzygapophyseal lamina) supporting the hyposphene or postzygapophysis from below: absent (0); present (1). (modified from Upchurch *et al.*[26]:ch. 146 by Carballido and Sander[40]:ch. 154)

157) Middle and posterior dorsal vertebrae, neural canal in anterior view: entirely surrounded by neural arch (0); enclosed in a deep fossa, enclosed laterally by pedicels (1). (Upchurch *et al.*[26]:ch. 136)

158) Middle and posterior dorsal vertebrae, neural spine height: approximately twice centrum length (0); four times centrum length (1). (modified from Upchurch *et al.*[26]:chs. 132, 166, 167 by Carballido and Sander[40]:ch. 156)

159) Middle and posterior dorsal neural spines, orientation: vertical (0); slightly inclined, with an angle of approximately 70º (1); strongly inclined, with an angle not larger than 40º (2). (modified from Wilson[44]:ch. 104 by Carballido and Sander[40]:ch. 157)

160) Middle and posterior dorsal neural arches, centropostzygapophyseal lamina shape: simple (0); divided (1). (Wilson[44]:ch. 95)

161) Middle and posterior dorsal neural arches, anterior centroparapophyseal lamina: absent (0); present (1). (Wilson[44]:ch. 96; Upchurch *et al.*[26]:ch. 133)

162) Middle and posterior dorsal neural arches, prezygoparapophyseal lamina: absent (0); present (1). (Wilson[44]:ch. 97)

163) Middle and posterior dorsal neural arches, posterior centroparapophyseal lamina: absent (0); present (1). (Wilson[44]:ch. 98; Upchurch *et al.*[26]:ch. 137)

164) Middle and posterior dorsal centra in transverse section (i.e., dorsoventral height to transverse width ratio): subcircular (ratio approximately 1.0 or slightly higher) (0); slightly dorsoventrally compressed (ratio between 0.8 and 1.0) (1); strongly dorsoventrally compressed (ratio lower than 0.8) (2). (modified from Upchurch *et al.*[26]:ch. 131 by Carballido and Sander[40]:ch. 162)

165) Middle and posterior dorsal neural spines, triangular aliform processes: absent (0); present but do not project far laterally (not as far as postzygapophyses) (1); present and

project far laterally (as far as postzygapophyses) (2). (modified from Wilson[44]:ch. 102; Upchurch *et al.*[26]:chs. 153–154 by Carballido and Sander[40]:ch. 163)

166) Middle and posterior dorsal vertebrae, spinodiapophyseal lamina: absent (0); present (1). (Upchurch *et al.*[26]:ch. 157)

167) Middle and posterior dorsal vertebrae, accessory spinodiapophyseal lamina: absent (0); present (1). (Upchurch *et al.*[26]:ch. 151)

168) Dorsal vertebrae, spinodiapophyseal webbing: lamina follows curvature of neural spine in anterior view (0); lamina 'festooned' from spine, dorsal margin does not closely follow shape of neural spine and diapophysis (1). (Whitlock[45]:ch.104)

169) Anterior dorsal vertebrae, spinopostzygapophyseal lamina: absent (0); present (1). (Upchurch *et al.*[54]:ch. 133)

170) Middle and posterior dorsal neural spines, lateral spinopostzygapophyseal lamina: absent (0); present (1). (Wilson[44]:ch. 100; Upchurch *et al.*[26]:ch. 159)

171) Middle and posterior dorsal neural arches, spinodiapophyseal lamina–spinopostzygapophyseal lamina contact: absent (0); present (1). (Wilson[44]:ch. 101)

172) Middle and posterior dorsal vertebrae, spinodiapophyseal lamina–spinopostzygapophyseal lamina contact: ventral, well separated from the triangular aliform process (0); dorsal, forms part of the triangular aliform process (1). (Carballido *et al.*[47]:ch. 170)

173) Middle and posterior dorsal vertebrae, height of neural arch ventral to postzygapophyses (i.e., neural arch pedicel): less than height of centrum (0); subequal to or greater than height of centrum (1). (Whitlock[45]:ch. 109)

174) Posterior dorsal vertebrae, medial spinopostzygapophyseal lamina: absent (0); present, forms part of the median posterior lamina (1). (Carballido *et al.*[47]:ch. 172)

175) Posterior dorsal vertebrae, transverse processes: lie posterior or posterodorsal to the parapophysis (0); lie vertically above the parapophysis (1). (Upchurch *et al.*[26]:ch. 139)

176) Posterior dorsal centra, articular face shape: amphicoelous (0); slightly opisthocoelous (1); opisthocoelous (2). (modified from Wilson[44]:ch. 105 by Carballido and Sander[40]:ch. 174)

177) Posterior dorsal vertebrae, neural spine: narrower transversely than anteroposteriorly (0); broader transversely than anteroposteriorly (1). (Wilson[44]:ch. 92)

178) Posterior dorsal vertebrae, posterior centrodiapophyseal lamina: has unexpanded ventral end (0); expands and may bifurcate toward ventral end (1). (Salgado *et al.*[50]:ch. 21)

Cervical and dorsal rib characters

179) Cervical ribs, distal shafts of longest cervical ribs: are elongate and form overlapping bundles (0); are short and do not project beyond the posterior end of the centrum to which they are attached (1). (Wilson[44]:ch. 140)

180) Cervical ribs, angle between capitulum and tuberculum: greater than 90°, so that the rib shaft lies close to the ventral edge of the centrum (0); less than 90°, so that the rib shaft lies below the ventral margin of the centrum (1). (Wilson[44]:ch. 139)

181) Dorsal ribs, proximal pneumatopores: absent (0); present (1). (Wilson[44]:ch. 141)

182) Anterior dorsal ribs, cross-sectional shape: subcircular (0); plank-like, anteroposterior breadth more than three times mediolateral breadth (1). (Wilson[44]:ch. 142)

Sacral characters

183) Sacral vertebrae, number: 3 or fewer (0); 4 (1); 5 (2); 6 (3). (Wilson[44]:ch. 108)

184) Sacrum, sacricostal yoke: absent (0); present (1). (Wilson[44]:ch. 109)

185) Sacral vertebrae contributing to acetabulum: numbers 1–3 (0); numbers 2–4 (1). (Wilson[44]:ch. 110)

186) Sacral neural spines, length: approximately twice length of centrum (0); approximately four times length of centrum (1). (Wilson[44]:ch. 111)

187) Sacral ribs, dorsoventral length: low, not projecting beyond dorsal margin of ilium (0); high, extending beyond dorsal margin of ilium (1). (Wilson[44]:ch. 112)

188) 'Pleurocoels' in lateral surfaces of sacral centra: absent (0); present (1). (Upchurch *et al.*[26]:ch. 165)

Caudal vertebral characters

189) Caudal vertebrae, number: 35 or fewer (0); 40–55 (1); increased to 70–80 (2). (Wilson[44]:ch.114)

190) Caudal bone texture: solid (0); spongy, with large internal cells (1). (Wilson[44]:ch. 113)

191) Caudal transverse processes: persist through caudal 20 or more posteriorly (0); disappear by caudal 15 (1); disappear by caudal 10 (2). (Wilson[44]:ch. 115)

192) First caudal centrum or last sacral vertebra, articular face shape: flat (0); procoelous (1); opisthocoelous (2); biconvex (3). (Wilson[44]:ch. 116)

193) First caudal neural arch, coel on lateral aspect of neural spine: absent (0); present (1). (Wilson[44]:ch. 117)

194) Anterior caudal vertebrae, ventral surface of transverse processes: directed laterally or slightly ventrally (0); directed dorsally (1). (Whitlock[45]:ch. 125)

195) Anterior caudal centra (excluding the first), articular face shape: amphiplatyan or amphicoelous (0); procoelous/distoplatyan (1); slightly procoelous (2); procoelous (3); (González Riga *et al.*[5]:ch. 52)

196) Anterior caudal centra, 'pleurocoels': absent (0); present (1). (Wilson[44]:ch. 119)

197) Anterior caudal vertebrae, ventral surfaces: convex transversely (0); concave transversely (1). (Upchurch *et al.*[26]:ch. 182)

198) Anterior and middle caudal vertebrae, ventrolateral ridges: absent (0); present (1). (Upchurch *et al.*[26]:ch. 183)

199) Anterior and middle caudal vertebrae, triangular lateral processes on neural spine: absent (0); present (1). (Whitlock[45]:ch. 123)

200) Anterior caudal transverse processes, shape: triangular, tapering distally (0); 'wing-like', not tapering distally (1). (Wilson[44]:ch. 128)

201) Anterior caudal neural spines, transverse breadth: approximately 50% of (0); or greater than anteroposterior length (1). (Wilson[44]:ch. 126)

202) Anterior caudal transverse processes, proximal depth: shallow, on centrum only (0); deep, extending from centrum to neural arch (1). (Wilson[44]:ch. 127)

203) Anterior caudal transverse processes, diapophyseal laminae: absent (0); present (1). (Wilson[44]:ch. 129)

204) Anterior caudal transverse processes, anterior centrodiapophyseal lamina, shape: single (0); divided (1). (Wilson[44]:ch. 130)

205) Anterior caudal vertebrae, hyposphenal ridge: absent (0); present (1). (Upchurch *et al.*[26]:ch. 187)

206) Anterior caudal centra, length: approximately the same (0); or doubling over the first 20 vertebrae (1). (Wilson[44]:ch. 120)

207) Anterior caudal neural arches, spinoprezygapophyseal lamina: absent, or present as small, short ridge that rapidly fades into anterolateral margin of neural spine (0); present, extending onto lateral aspect of neural spine (1). (modified from Wilson[44]:ch. 121 by Carballido and Sander[40]:ch. 205)

208) Anterior caudal neural arches, spinoprezygapophyseal–spinopostzygapophyseal lamina contact: absent (0); present, forming a prominent lamina on lateral aspect of neural spine (1). (Wilson[44]:ch. 122)

209) Anterior caudal neural arches, prespinal lamina: absent (0); present (1). (Wilson[44]:ch. 123)

210) Middle caudal centra, shape: cylindrical (0); with flat ventral margin (1); quadrangular, flat ventrally and laterally (2). (modified from Wilson[44]:ch. 131 by Carballido and Sander[40]:ch. 208)

211) Anterior and middle caudal centra, ventral longitudinal hollow: absent (0); present (1). (Wilson[44]:ch. 132)

212) Middle caudal centra, articular face shape: amphiplatyan or amphicoelous (0); procoelous/distoplatyan (1); slightly procoelous (2); procoelous (3). (González Riga *et al.*[5]:ch. 53)

213) Middle caudal vertebrae, location of neural arches: over midpoint of centrum, with approximately subequal amounts of centrum exposed at either end (0); on anterior half of centrum (1). (modified from Salgado *et al.*[50]:ch. 15 by Upchurch *et al.*[26]:ch. 185)

214) Middle caudal vertebrae, height of pedicel ventral to prezygapophysis: low, with curved anterior edge of pedicel (0); high, with vertical anterior edge of pedicel (1). (Carballido *et al.*[47]:ch. 212)

215) Middle caudal vertebrae, orientation of neural spines: anterior (0); vertical (1); slightly posterior (2); strongly posterior (3). (modified from Wilson[44]:ch. 133 by Carballido and Sander[40]:ch. 213)

216) Posterior caudal vertebrae, neural spine strongly displaced posteriorly: absent (0); present (1). (Carballido *et al.*[47]:ch. 214)

217) Middle caudal vertebrae, ratio of centrum length to height: less than 2.0, usually 1.5 or less (0); 2.0 or higher (1). (Upchurch *et al.*[26]:ch. 179)

218) Anterior-most posterior caudal vertebrae (i.e., those that retain a well-developed neural spine), neural spine orientation: vertical (0); slightly posterior (1); strongly posterior (2). (Carballido *et al.*[47]:ch. 216)

219) Posterior caudal centra, articular face shape: amphiplatyan (0); procoelous (1); opisthocoelous (2). (modified from González Riga *et al.*[5]:ch. 54 by Carballido and Sander[40]:ch. 217)

220) Posterior caudal centra, shape: cylindrical (0); dorsoventrally flattened, breadth at least twice height (1). (Wilson[44]:ch. 135)

221) Posterior caudal vertebrae, ratio of length to height: less than 5.0, usually 3.0 or less (0); 5.0 or higher (1). (Upchurch *et al.*[26]:ch. 180)

222) Posterior-most caudal centra, articular face shape: platycoelous (0); biconvex (1). (Wilson[44]:ch. 136)

223) Posterior-most biconvex caudal centra, number: 10 or fewer (0); more than 30 (1). (Wilson[44]:ch. 137)

224) Posterior-most biconvex caudal centra, length to height ratio: less than 4.0 (0); greater than 5.0 (1). (Wilson[44]:ch. 138)

Haemal arch (= chevron) characters

225) Forked haemal arches with anterior and posterior projections: absent (0); present (1). (Wilson[44]:ch. 143)

226) Forked haemal arches, distribution: posterior tail only (0); throughout middle and posterior caudal vertebrae (1). (Wilson[44]:ch. 144)

227) Haemal arches, ‘crus’ bridging dorsal margin of haemal canal: present (0); absent (1). (Wilson[44]:ch. 145)

228) Haemal canal, depth: short, approximately 25% of haemal arch length (0); or long, approximately 50% haemal arch length (1). (Wilson[44]:ch. 146)

229) Haemal arches: persisting throughout at least 80% of tail (0); disappearing by caudal 30 (1). (Wilson[44]:ch. 147)

230) Posterior haemal arches, distal contact: fused (0); unfused (open) (1). (Wilson[44]:ch. 148)

General appendicular skeletal characters

231) Posture: bipedal (0); columnar, obligatory quadrupedal posture (1). (Wilson[44]:ch. 149)

Pectoral girdle characters

232) Scapular acromion process, size: narrow (0); broad, width more than 150% minimum width of blade (1). (Wilson[44]:ch. 150)

233) Scapular blade, orientation with respect to coracoid articulation: perpendicular (0); forming a 45° angle (1). (Wilson[44]:ch. 151)

234) Scapular blade, shape: acromial edge not expanded (0); rounded expansion on acromial side (1); racquet-shaped (2). (Wilson[44]:ch. 152)

235) Scapula, acromion process dorsal margin: concave or straight (0); with V-shaped concavity (1); with U-shaped concavity (2). (Sereno *et al.*[52]:ch. 88)

236) Scapula, highest point of the dorsal margin of the blade: lower than the dorsal margin of the proximal end (0); at the same height than the dorsal margin of the proximal end (1); higher than the dorsal margin of the proximal end (2). (Carballido *et al.*[47]:ch. 234, from Mannion[55])

237) Scapula, development of the acromion process: undeveloped (0); well developed (1). (Carballido *et al.*[47]:ch. 235)

238) Scapular length/minimum blade breadth: 5.5 or less (0); 5.5 or more (1). (Carballido *et al.*[47]:ch. 236)

239) Scapula, ventral margin with well-developed ventromedial process: absent (0); present (1). (Carballido *et al.*[53]:ch. 202)

240) Scapular, acromial process position: lies nearly at the level of the glenoid (0); lies nearly at the midpoint of the scapular body (1). (Carballido *et al.*[47]:ch. 238)

241) Scapular acromion length: less than one-half scapular length (0); at least one-half scapular length (1). (Mannion *et al.*[48]:ch. 168)

242) Scapular glenoid orientation: relatively flat or laterally facing (0); strongly bevelled medially (1). (Wilson[44]:ch. 153)

243) Scapular blade, cross-sectional shape at base: flat or rectangular (0); D-shaped (1). (Wilson[44]:ch. 154)

244) Coracoid, proximodistal length: less than length of scapular articulation (0); approximately twice length of scapular articulation (1). (Wilson[44]:ch. 155)

245) Coracoid, anteroventral margin shape: rounded (0); rectangular (1). (modified from Salgado *et al.*[50]:ch. 29 by Wilson[44]:ch. 156)

246) Dorsal margin of coracoid in lateral view: reaches or surpasses the level of the dorsal margin of the scapular proximal expansion (0); lies below the level of the scapular

proximal expansion and is separated from the latter by a V-shaped notch (1). (Upchurch *et al.*[26]:ch. 207)

247) Coracoid, infraglenoid deep groove: absent (0); present (1). (Carballido *et al.*[47]:ch. 245)

248) Coracoid, infraglenoid lip: absent (0); present (1). (Wilson[44]:ch. 157)

249) Sternal plate, shape: oval (0); crescentic (1). (modified from Salgado *et al.*[50]:ch. 26 by Wilson[44]:ch. 158)

250) Prominent posterolateral expansion of sternal plate producing kidney-shaped profile in dorsal view: absent (0); present (1). (Upchurch *et al.*[26]:ch. 211)

251) Prominent parasagittally-oriented ridge on dorsal surface of sternal plate: absent (0); present (1). (Upchurch *et al.*[26]:ch. 212)

252) Ridge on ventral surface of sternal plate: absent (0); present (1). (Upchurch *et al.*[26]:ch. 213)

253) Ratio of maximum length of sternal plate to humerus length: less than 0.75, usually less than 0.65 (0); greater than 0.75 (1). (Upchurch *et al.*[26]:ch. 209)

Forelimb characters

254) Humerus to femur proximodistal length ratio: less than 0.60 (0); 0.60 to 0.90 (1); greater than 0.90 (2). (Upchurch *et al.*[26]:ch. 216)

255) Humeral deltopectoral attachment, development: prominent (0); reduced to a low crest or ridge (1). (Wilson[44]:ch.160)

256) Humeral deltopectoral crest, shape: relatively narrow throughout length (0); markedly expanded distally (1). (Wilson[44]:ch.161)

257) Humerus, ratio of mediolateral width of proximal end to total proximodistal length: less than 0.4 (0); 0.4 or greater (1). (modified from Mannion et al.[41]*:ch. 41 by this paper)*

258) Humerus, ratio of minimum mediolateral width of diaphysis to total proximodistal length: less than 0.2 (0); 0.2 or greater (1). (modified from Curry Rogers[42]:ch. 265 by this paper)

259) Humeral midshaft cross-section, shape: circular (0); elliptical (1). (Mannion *et al.*[48]:ch. 170)

260) Humerus, Robustness Index (*sensu* Wilson and Upchurch[56]): gracile (less than 0.27) (0); medium (0.28–0.32) (1); robust (more than 0.33) (2). (Carballido *et al.*[47]:ch. 256)

261) Humeral distal condyles, articular surface shape: restricted to distal portion of humerus (0); exposed on anterior portion of humeral shaft (1). (Wilson[44]:ch. 163)

262) Humeral distal condyle, shape: divided (0); flat (1). (Wilson[44]:ch. 164)

263) Humeral, lateral margin: medially deflected (0); almost straight until midlength or more (1). (Carballido *et al.*[47]:ch. 259)

264) Humeral proximolateral corner, shape: rounded, proximal surface is smoothly convex (0); pronounced or square, proximal surface is low, almost flat (1). (Wilson[44]:ch. 159)

265) Ulnar proximal condyle, shape: subtriangular (0); triradiate, with deep radial fossa (1). (Wilson[44]:ch. 165)

266) Ulnar proximal condylar processes, relative lengths: subequal (0); anterior process longer (1). (Wilson[44]:ch. 166)

267) Ulnar olecranon process, development: prominent, projecting above proximal articulation (0); rudimentary, level with proximal articulation (1). (Wilson[44]:ch. 167)

268) Ulna, length to proximal breadth ratio: gracile (0); stout (1). (Wilson[44]:ch. 168)

269) Radial distal condyle, shape: round (0); subrectangular, flattened posteriorly and articulating in front of ulna (1). (Wilson[44]:ch. 169)

270) Radius, distal breadth: slightly greater than midshaft breadth (0); approximately twice midshaft breadth (1). (Wilson[44]:ch.170)

271) Radius, distal condyle orientation: perpendicular to long axis of shaft (0); bevelled approximately 20º proximolaterally relative to long axis of shaft (1). (Wilson[44]:ch. 171)

272) Carpal bones, number: three or more (0); two or fewer (1). (Wilson[44]:ch. 173)

273) Carpal bones, shape: round (0); block-shaped, with flattened proximal and distal surfaces (1). (Wilson[44]:ch. 174)

274) Metacarpus, shape: spreading (0); bound, with subparallel shafts and articular surfaces that extend half their length (1). (Wilson[44]:ch. 175)

275) Metacarpals, shape of proximal surface in articulation: gently curving, forming a 90° arc (0); U-shaped, subtending a 270° arc (1). (Wilson[44]:ch. 176)

276) Longest metacarpal to radius ratio: close to 0.3 (0); 0.45 or more (1). (Wilson[44]:ch. 177)

277) Metacarpal I, length: shorter than metacarpal IV (0); longer than metacarpal IV (1). (Wilson[44]:ch. 178)

278) Metacarpal I, distal condyle shape: divided (0); undivided (1). (Wilson[44]:ch. 179)

279) Metacarpal I distal condyle, transverse axis orientation: bevelled approximately 20º proximodistally with respect to axis of shaft (0); perpendicular with respect to axis of shaft (1). (Wilson[44]:ch. 180)

280) Manual digits II and III, phalangeal number: 2-3-4-3-2 or more (0); reduced, 2-2-2-2-2 or less (1); absent or unossified (2). (Wilson[44]:ch. 181)

281) Manual phalanx I-1, shape: rectangular (0); wedge-shaped (1). (Wilson[44]:ch. 182)

282) Manual non-ungual phalanges, shape: longer proximodistally than broad transversely (0); broader transversely than long proximodistally (1). (Wilson[44]:ch. 183)

Pelvic girdle characters

283) Pelvis, anterior breadth: narrow, ilia longer anteroposteriorly than distance separating preacetabular processes (0); broad, distance between preacetabular processes exceeds anteroposterior length of ilia (1). (Wilson[44]:ch. 184)

284) Ilium, ischial peduncle size: large, prominent (0); low, rounded (1). (Wilson[44]:ch. 185)

285) Ilium, dorsal margin shape: flat (0); semi-circular (1). (Wilson[44]:ch. 186)

286) Ilium, preacetabular process shape: pointed, arching ventrally (0); semi-circular, with posteroventral excursion of cartilage cap (1). (Wilson[44]:ch. 188)

287) Ilium, preacetabular process orientation: anterolateral to body axis (0); perpendicular to body axis (1). (Wilson[44]:ch. 189)

288) Highest point on dorsal margin of ilium: lies posterior to base of pubic peduncle (0); lies anterior to base of pubic peduncle (1). (Upchurch *et al.*[26]:ch. 245)

289) Pubis length with respect to ischium: pubis slightly shorter than or subequal to ischium (0); pubis longer (>120%) than ischium (1). (Carballido *et al.*[47]:ch. 285)

290) Pubis, ambiens process development: small, confluent with anterior margin of pubis (0); prominent, projects anteriorly from anterior margin of pubis (1). (Wilson[44]:ch. 189)

291) Pubic apron, shape: flat (straight symphysis) (0); canted anteromedially (gentle S-shaped symphysis) (1). (Wilson[44]:ch. 190)

292) Puboischial contact, length: approximately one third total length of pubis (0); one-half total length of pubis (1). (Wilson[44]:ch. 191)

293) Ischium, acetabular articular surface: maintains approximately the same transverse width throughout its length (0); is transversely narrower in its central portion and strongly expanded as it approaches the iliac and pubic articulations (1). (Mannion *et al.*[48]:ch. 180)

294) Ischium, iliac peduncle with constriction or 'neck': absent (0); present (1). (Whitlock[45]:ch. 173)

295) Ischium, elongate muscle scar on proximal end: absent (0); present (1). (Whitlock[45]:ch. 174)

296) Ischial blade, shape: emarginated distal to pubic peduncle (0); no emargination distal to pubic peduncle (1). (Wilson[44]:ch. 193)

297) Ischium, proximodistal length of pubic peduncle: less than or equal to anteroposterior length of pubic peduncle (0); greater than anteroposterior length of pubic peduncle (1). (Salgado *et al.*[50]:ch. 13)

298) Ischium, anteroposterior width of pubic peduncle divided by total length of ischium: less than 0.5 (0); 0.5 or greater (1); large (2). (Carballido *et al.*[47]:ch. 294)

299) Ischial distal shaft, shape: triangular, depth of ischial shaft increases medially (0); bladelike, medial and lateral depths subequal (1). (Upchurch *et al.*[26]:ch. 194)

300) Ischial distal shafts, cross-sectional shape: V-shaped, forming an angle of nearly 50º with each other (0); flat, nearly coplanar (1). (Wilson[44]:ch. 195)

301) Ischia, distal end: slightly expanded (0); strongly expanded dorsoventrally (1). (Upchurch[43]:ch. 183)

302) Ischium, angle formed between shaft and acetabular margin: forming a nearly right angle (80–110°) (0); forming an acute angle (less than 70°) (1). (Carballido *et al.*[47]:ch. 298)

Hind limb characters

303) Femur, fourth trochanter development: prominent (0); reduced to crest or ridge (1); extremely reduced (2). (modified from Wilson[44]:ch. 196 following Whitlock[45]:ch. 186 by Carballido and Sander[40]:ch. 299)

304) Femur, lesser trochanter: present (0); absent (1). (Wilson[44]:ch. 197)

305) Femur midshaft transverse diameter: subequal to anteroposterior diameter (0); 125–150% anteroposterior diameter (1); at least 185% anteroposterior diameter (2). (Wilson[44]:ch. 198)

306) Femur, lateral bulge (marked by the lateral expansion and proximomedial inclination of the proximolateral margin of the femur, which begins distal to the distal margin of the femoral head): absent (0); present (1). (Salgado *et al.*[50]:ch. 19)

307) Femur, pronounced ridge on posterior surface between greater trochanter and head: absent (0); present (1). (Whitlock[45]:ch. 181)

308) Femur, head position: perpendicular to shaft, rises at same level as greater trochanter (0); proximally directed, rises well above level of greater trochanter (1). (modified from Upchurch *et al.*[26]:ch. 263 by Carballido and Sander[40]:ch. 304)

309) Femur, relative transverse breadth of distal condyles: subequal (0); tibial condyle much broader than fibular condyle (1). (Wilson[44]:ch. 200)

310) Femur, orientation of distal condyles: perpendicular or slightly bevelled dorsolaterally (0); or bevelled dorsomedially approximately 10° relative to femoral shaft (1). (Wilson[44]:ch. 201)

311) Femur, shape of articular surface of distal condyles: restricted to distal portion of femur (0); expanded onto anterior portion of femoral shaft (1). (Wilson[44]:ch. 202)

312) Position of femoral fourth trochanter: on posterior surface of shaft, near mediolateral midline (0); on posteromedial margin of shaft (1). (Upchurch *et al.*[26]:ch. 268)

313) Tibial proximal condyle, shape: narrow, long axis anteroposterior (0); expanded transversely, condyle subcircular (1). (Wilson[44]:ch. 203)

314) Tibial cnemial crest, orientation: projecting anteriorly (0); or laterally (1). (Wilson[44]:ch. 204)

315) Tibia, distal breadth: approximately 125% (0); more than twice midshaft breadth (1). (Wilson[44]:ch. 205)

316) Tibial distal posteroventral process, size: broad transversely, covering posterior fossa of astragalus (0); shortened transversely, posterior fossa of astragalus visible posteriorly (1). (Wilson[44]:ch. 206)

317) Fibula, proximal tibial scar, development: not well-marked (0); well-marked and deepening anteriorly (1). (Wilson[44]:ch. 207)

318) Fibula, lateral trochanter: absent (0); present (1). (Wilson[44]:ch. 208)

319) Fibular distal condyle, size: subequal to shaft (0); expanded transversely, more than twice midshaft breadth (1). (Wilson[44]:ch. 209)

320) Astragalus, shape: rectangular (0); wedge-shaped, with reduced anteromedial corner (1). (Wilson[44]:ch. 210)

321) Astragalus, fibular facet: faces laterally (0); faces posterolaterally, anterior margin visible in posterior view (1). (Whitlock[45]:ch. 186)

322) Astragalus, foramina at base of ascending process: present (0); absent (1). (Wilson[44]:ch. 211)

323) Astragalus, ascending process length: limited to anterior two-thirds of astragalus (0); extending to posterior margin of astragalus (1). (Wilson[44]:ch. 212)

324) Astragalus, posterior fossa shape: undivided (0); divided by vertical crest (1). (Wilson[44]:ch. 213)

325) Astragalus, transverse length: 50% more than (0); or subequal to proximodistal height (1). (Wilson[44]:ch. 214)

326) Calcaneum: present (0); absent or unossified (1). (Wilson[44]:ch. 215)

327) Distal tarsals 3 and 4: present (0); absent or unossified (1). (Wilson[44]:ch. 216)

328) Metatarsus, posture: bound (0); spreading (1). (Wilson[44]:ch. 217)

329) Metatarsal I proximal condyle, transverse axis orientation: perpendicular to axis of shaft (0); angled ventromedially approximately 15º to axis of shaft (1). (Wilson[44]:ch. 218)

330) Metatarsal I distal condyle, transverse axis orientation: perpendicular to (0); angled dorsomedially to axis of shaft (1). (Wilson[44]:ch. 219)

331) Metatarsal III length divided by metatarsal I length less (0) or more (1) than 1.3. (this paper)

332) Metatarsal I distal condyle, posterolateral projection: absent (0); present (1). (Wilson[44]:ch. 220)

333) Metatarsal I, minimum shaft width: less than that of metatarsals II–IV (0); or greater than that of metatarsals II–IV (1). (Wilson[44]:ch. 221)

334) Longest metatarsal: metatarsal III (0); metatarsal IV (1). (this paper)

335) Metatarsals I and V, proximal condyle size: smaller than (0); or subequal to those of metatarsals II and IV (1). (Wilson[44]:ch. 222)

336) Metatarsal III length: more than 30% (0); or less than 25% that of tibia (1). (Wilson[44]:ch. 223)

337) Metatarsals III and IV, minimum transverse shaft diameters: subequal to (0); or less than 65% that of metatarsals I or II (1). (Wilson[44]:ch. 224)

338) Metatarsal V, length: shorter than (0); or at least 70% length of metatarsal IV (1). (Wilson[44]:ch. 225)

339) Pedal non-ungual phalanges, shape: longer proximodistally than broad transversely (0); broader transversely than long proximodistally (1). (Wilson[44]:ch. 226)

340) Pedal digits II–IV, penultimate phalanges, development: subequal in size to more proximal phalanges (0); rudimentary or absent (1). (Wilson[44]:ch. 227)

341) Pedal unguals, orientation: aligned with (0); or deflected lateral to digit axis (1). (Wilson[44]:ch. 228)

342) Pedal digit I ungual, length relative to pedal digit II ungual: subequal (0); 25% larger than that of digit II (1). (Wilson[44]:ch. 229)

343) Pedal digit I ungual, length: shorter (0); or longer than metatarsal I (1). (Wilson[44]:ch. 230)

344) Pedal ungual I, shape: broader transversely than dorsoventrally (0); sickle-shaped, much deeper dorsoventrally than broad transversely (1). (Wilson[44]:ch. 231)

345) Pedal ungual II–III, shape: broader transversely than dorsoventrally (0); sickle-shaped, much deeper dorsoventrally than broad transversely (1). (Wilson[44]:ch. 232)

346) Pedal digit IV ungual, development: subequal in size to unguals of pedal digits II and III (0); rudimentary or absent (1). (Wilson[44]:ch. 233)

347) Unguals of pedal digits II and III, proximal dimensions: as broad as deep (0); significantly broader than deep (1). (Allain and Aquesbi[57]:ch. 253)

348) Number of phalanges in pedal digit II: three (0); two (1). (this paper)

349) Number of phalanges in pedal digit III: four (0); three (1); two (2). (this paper)

350) Number of phalanges in pedal digit IV: three or more (0); two (1); one (2). (modified from Upchurch[43]:chs. 200 and 201 by this paper)

X. Phylogenetic data matrix

We scored all taxa included in our phylogenetic analysis for the nine newly added anatomical characters (as above, numbers 131, 132, 257, 258, 331, 334, and 348–350) and the slightly modified character (133) proposed herein using the technical literature and personal observations. The remaining 340 characters (1–130, 134–256, 259–330, 332, 333, and 335–347) were assembled by Carballido and Sander[40] and reemployed by Lacovara *et al.*[28] and Poropat *et al.*[58]. Scores for *Dreadnoughtus schrani* and *Futalognkosaurus dukei* for these 340 characters are taken from Lacovara *et al.*[28], whereas those for *Diamantinasaurus matildae* are from Poropat *et al.*[58]. Scores for characters 239, 272, and 310 are also as presented by Lacovara *et al.*[28]:supplementary information (characters 237, 268, and 306 in that analysis and in Carballido and Sander[40] and Poropat *et al.*[58]).

Because this study deals in large part with the evolution of the sauropod hind foot, we placed particular emphasis on the scoring of pedal morphologies in our phylogenetic data matrix. We revised Carballido and Sander's[40] scores for 14 pedal characters (our numbers 328–330, 333, 335, and 339–347) in *Cedarosaurus weiskopfae* based both on the holotype of this titanosauriform (DMNH 39045; see Tidwell *et al.*[59]) and the recently referred distal hind limb FMNH PR 977[60,61]. We also recoded the somphospondylan *Ligabuesaurus leanzai* for ten characters (328–330, 332, 333, and 335–339) based on the well-preserved metatarsus of the holotype (MCF-PVPH 233; see Bonaparte *et al.*[62]). Further, we rescored *Alamosaurus*

sanjuanensis for 14 pedal characters (328–330, 332, 333, 335, 337–339, 341, 343–345, and 347) based on the nearly complete distal hind limb that is likely referable to this taxon (NMMNH-P 49967; see D'Emic *et al.*[37]). We recoded six characters (our numbers 112, 225, and 344–347) in *Mendozasaurus*, 38 characters (186, 201–204, 208, 211, 213, 217, 225–231, 239, 249, 254, 256, 272–282, 290, 296, 298, 308, 326, 327, 347) in *Epachthosaurus sciuttoi*, one character (138) in *Malawisaurus dixeyi*, and one character (134) in *Opisthocoelicaudia skarzynskii* based on personal observations and/or reinterpretations of the published morphologies of these species. Scores for *Notocolossus* are based on the data presented herein. Other scores for the 340 characters that were also employed by Carballido and Sander[40] are as in that analysis. The matrix is provided below; also, .nex and .tnt versions are available from the senior author (B.J.G.R.) upon request.

Plateosaurus engelhardti
0000000000 0000000000 0000000010 0000000010 000?00000? 0000??0000 0000000000
0??00000?0 000???0000 000000000? 0-00?00000 0?00-10100 0?000?0000 0000-00000
0000-1-0-0 0000000000 000000--00 --0000000? ???0?000?0 ???000??0? 000-??0-00
00002?0100 00????0??? 00000000?0 00000?0?0? ??00000000 0000000?00 0000001000
000000000? 0000000?00 0000000000 0?00000000 0000000000 1000000000 0000000000

Shunosaurus lii
0100100000 1101100000 10000000?? 000001001? ?000000110 1100000100 0110000000
00??000001 100?0000?1 01?110?101 1011?20120 1001000100 0000100001 0000-10100
0110-1-1-0 0000000000 11101(01)--11 (01)?00001000 0010-00010 1000000000 0100100-00
?0002001?0 0---111010 100000010? ?000010000 1001100011 0100101010 0010000011
0101100000 100000???0 ?011100110 001101?100 ?10?001111 1010110111 ?111110010

Omeisaurus spp.
11001?0000 1101110000 10???000?? ?001010011 0000010110 11?000?1?? ?11100?0?0
??00?0?001 10000000?1 ???111?101 1011010040 111230000(12) 0001011001 0000-21000
(12)111001100 000100100? 1111010011 1-00021001 0?2110100? 1000000000 0100100-00
?0002001?0 0???110000 1100000??0 00?0000000 100110(01)011 0100101010 0010000001
1101100000 1000000010 ?111100000 0001011??0 0101001111 (01)110111111 1111110010

Camarasaurus spp.
1110100000 1111110100 0000000000 1001010001 0100010110 1110001101 ?111000000
0000100001 1000001111 1111110?01 1011020010 101232?0(01)1 0100101101 0001121001
1110-00101 1000001000 1101210011 1100021001 0?21101110 1000000000 1100100-10
0000200100 00--(01)0(01)010 1101000101 0010010000 0001100011 0100111010 0111111001
1111100000 1100001011 0011100010 0111011101 0111001111 (01)010111111 111111000?

Euhelopus zdanskyi
01?0100001 11??110??? ???0?????? ?00??????? ????0????? ?11?????01 ?1???????? ??????????
100???1??? ???111??11 1011?2004? 1112201012 000010?1?0 1001111111 2101101111
10??0?100? 1110111011 1100021101 113??01??? ?????????? ?????????? ?????????? ??????????
1100000?11 0?000?00?? ????100011 0101?????? ?????????? ??1110?100 1100001011
0011110010 0011011101 ?11?00?111 1010?11?1? ??11??0???

Brachiosaurus altithorax
12101?00?? 11??1????? ??????000? 10?1110?01 000?01011? ?110?????? ????0?0000
00111??0?1 ?????????? ???1210?0? 1011?201?? 11?232?002 000111???1 00????10?1
1111001100 000010100? 1111210011 10010211?? ?????????? ???0?????? ??????????
?????????? ?????????? ?????????? ?????????? ????100010 0001?????? ?????????? ??????????
?????????? ??11200010 00???????? 0????????? ?????????? ??????????

Giraffatitan brancai
1210100000 1111110100 1000100000 1001010001 0000010110 1110000101 ?111000000
001110?001 1000001111 1121210?00 10110201?0 111232(01)002 0001111101 0000-21001
1111001101 0200101010 11(01)1210011 1001021?01 11211011?0 1000100000 0100100-10
0010200100 0?????10?? 1101000?00 0010011000 0002100010 0101111010 0111111111
0111110110 1100001011 0011210010 0111011111 ?111001111 ?01?11?11? 1??11?0???

Venenosaurus dicrocei
?????????? ?????????? ?????????? ?????????? ?????????? ?????????? ?????????? ??????????
?????????? ?????????? ?????????? ?????????? ?????????? ?????????? ?????????? ??????????
?????????? ?????????? 11???????0 ???0-10??0 0?00100010 0010000?00 0?????????
11?00001?1 0?1??????? ?????????? ????1?1010 0????1???? ???????10 1?0000101? 00????????
?????????? ?????????? ?????????? ??????????

Cedarosaurus weiskopfae
?????????? ?????????? ?????????? ?????????? ?????????? ?????????? ?????????? ??????????
?????????? ?????????? ?????????? ?????????? ?????????? ??????100? 2????????? ????0?????
1????????? ?????2?1?? ?????????0 ???010??00 0?0?0?0010 ?110000010 0?????????
??0?00010? ??1011???? ???2100000 0111111??0 0????????? ?????????? ??????????
??11210110 ????0????? ???????111 1?1?1???11 1111100???

Chubutisaurus insignis
?????????? ?????????? ?????????? ?????????? ?????????? ?????????? ?????????? ??????????
?????????? ?????????? ?????????? ?????????? ?????????? ??????1??? 2?10-0011? 110??01?10
???1?10??1 0-00?210?? 11???????0 10?01000?0 ?10??0?-?0 001???0?00 0?????10??
1110000111 001??????? ???1100010 01111??011 0??111111? ?????????? ???0001011
0111210110 011101???? ?????????? ?????1???? ??????????

Wintonotitan wattsi
?????????? ?????????? ?????????? ?????????? ?????????? ?????????? ?????????? ??????????
?????????? ?????????? ?????????? ?????????? ?????????? ??????1??? 21?121-?1? 20????????
?????1??1? ?????21??? ???????00 1??01011?? ?????0?010 001???0?00 0?????10??
1110000111 0?1??????? ?????00010 00??1??0?1 0??111???? ???11????? ???00010?1
0????????? ?????????? ?????????? ?????????? ??????????

Ligabuesaurus leanzai
?????????? ?????????? ?????????? ?????????? ?????????? ?????????? ?????????? ??????????
?????????? ????(012)?0?00 1011?201?? 1?12?3?002 ??0???0000 1010-?1001 210111-111

110000101? 11?1110011 1100021l?? ?????????? ?????????? ?????????? ?????????? ??????????
?110000111 011??????? ???2100010 0011?????? ?????????? ?????????? ?????????? ??11210?10
0111011101 ???????111 11101111?? ??????????

Phuwiangosaurus sirindhornae
?????????? ?????????? ???10????? 100101???? ????0????? ?111?????? ????100011 0?1100?0?1
?????????? ?????????? 1221?201(234)? 101223?0?2 0100111001 1?01?21(01)01 2111100111
1101001010 111121(01)011 110002101? ??2????1?? ???0100?00 010-1?0011 0011100100
00--0-10?? 1100000100 01??????11 ????10?0?0 000?110111 0????????? ???1100110
110001?011 001121?010 011111110? ?????????? ?????????? ??????????

Andesaurus delgadoi
?????????? ?????????? ?????????? ?????????? ?????????? ?????????? ?????????? ??????????
?????????? ?????????? ?????????? ?????????? ?????????? ??????10?1 2?10-1-?11 1??0?0101?
111?110011 11000211?? ????????00 1?10201100 0?0?00???0 ?01020010? 0?????????
????????0? ?????????? ?????????? ?????????? ?????????? ????????10 11000110?? 00??2?????
?1???????? ?????????? ?????????? ??????????

Mendozasaurus neguyelap
?????????? ?????????? ?????????? ?????????? ?????????? ?????????? ?????????? ??????????
?????????? ?????????? ?????????? 111????002 ??0???0001 2110-?1000 21?121-112 ????0??01?
??????0?1? ?????????? ?????????0 ???0200000 0100000010 ?210200110 ????0?????
1110000110 011?????1? ????1000?1 00011??0?? ???11????? ?????????? ??????????
??112101?? ???10??1?? ?????????? 1?11?1???? ???1110121

Argentinosaurus huinculensis
?????????? ?????????? ?????????? ?????????? ?????????? ?????????? ?????????? ??????????
?????????? ?????????? ?????????? ?????????? ?????????? ??????1001 20?121-112 110000001?
1111211011 11000211?? ??(23)??????? ?????????? ?????????? ?????????? ??????????
?????????? ?????????? ?????????? ?????????? ?????????? ??1??????? ?????????? ??????????
????0????? ?????????? ?????????? ??????????

Epachthosaurus sciuttoi
?????????? ?????????? ?????????? ?????????? ?????????? ?????????? ?????????? ??????????
?????????? ?????????? ?????????? ?????????? ?????????? ??????1001 2110-1-111 1?0000002?
1112111011 11000211?? 103??0???? 110030??00 ?10010?010 1310100010 ????0-1111
1????????? ????????1? ???1100011 00011?00?1 01-1111112 --11?11?10 1??00?????
??11210110 01?101?101 ????11111 1011111111 ?011110111

Malawisaurus dixeyi
1120?????? ?1??1????? ?????????? ?????????? ?????????? ?????????? 0????????? ??????0???
100??????? ???1?10??? 1?21?20??? 101303?102 1010110?01 0000-?1001 211121-112
2?1200?020 1?01110?11 110?02110? 1????????? 1??0300?00 0100??0-10 1010100000
????0-11?? 1????????? ???00?0011 0???100011 011110001? 0??1??111? ???1??????
???0011111 00?1210??? ???11110? ???????1?? ?????????? ???1??????

Rapetosaurus krausei
00201?1?10 12?1010?01 ?1110?0011 ?00001?0?0 110?01010? 1111??2111 1???11?010
0???1110?1 101??011?? ???1210?00 1231?20?40 101303?102 1010110101 0010-?1000
2(01)0121-112 2112000020 1112011011 1-011211?? ??3??????? ???03????? ??00??0???
?3?020?21? ?????????? 1110000??? ???00?001? ????100012 10011?0011 0????1?1??
??11110110 110001??11 ?011?11110 1????????? ?????????? 1??0?????? ??????????

Isisaurus colberti
?????????? ?????????? ?????????? ?????????? ?????????? ?????????? ?????????? ??????????
?????????? ?????????? ?????????? 10?301?102 1010100001 1010-?1000 200121-111
2?12001000 1110010011 1-0?1210?1 ?13110?0?? ???030?100 1100000-10 1310200110
????0-11?0 10100000?0 01000?00?? ????100011 11?11101?? ?????????? ??110111?0
1100011111 01???????? ?????????? ?????????? ?????????? ??????????

Tapuiasaurus macedoi
002?111?00 12?1010?01 11100??011 1011?1000? 0???01011? 1?1????111 11???1?000
0???1110?? 101????0?? 1??1210?00 1231020(23)?? 1??????0?2 ?0??11???? ?0????1000
21?121-111 2?120?0000 110??11011 1????11??? 11???????? ?????????? ?????????? ??????????
?????????? ?????????? ???10?101? ????????1? ????110011 ?????1???? ?????????? ??????????
??1?2?????? ??????1??? ?????????? ????????11 10?111????

Trigonosaurus pricei
?????????? ?????????? ?????????? ?????????? ?????????? ?????????? ?????????? ??????????
?????????? ?????????? ?????????? 101103?102 0?00110001 1000-31(01)11 210121-112
2(02)1200002? 1112111011 1?0?1211?? ?????????? ?????????? ?????????? ??????????
?????????? ?????????? ?????????? ?????????? ?????????? ?????????? ?????????? ??????????
?????????? ?????????? ?????????? ?????????? ??????????

Alamosaurus sanjuanensis
?????????? ?????1???? ?????????? ?????????? ?????????? ?????????? ?????????? ??????????
?????????? ?????????? 1231?201?? 1?1303?102 000?000001 0000-?10?1 (12)10121-1?1
2212000020 110211?011 11000210?1 ?1(23)1?0??00 2310300100 1100000-10 1310111010
0???0-1111 1110000010 0111111011 0?1?11?010 1001110011 11-1111112 --?????1??
??00?11111 00112???11 1??111111? ??????1111 11111?111? 1?111?0???

Opisthocoelicaudia skarzynskii
?????????? ?????????? ?????????? ?????????? ?????????? ?????????? ?????????? ??????????
?????????? ?????????? ?????????? ?????????? ?????????? ???1??1001 200121-11(12)
2212001020 1112111011 11001210?? 1131?0?00? 2210-01100 1100100-10 1-10200020
01000-1111 10000000?0 0111101111 0111111112 1011110111 11-1111112 --11111110
110001?111 0111210111 0111111101 0110111111 1010111111 1011110122

Neuquensaurus australis
?????????? ?????????? ?????????? ?????????? ?????????? ?????????? ?????????? ??????????
?????????? ?????????? ?????????? 101(23)030002 ??00??0001 1000-?1111 200121-112
211200002? 1112111011 11001211?? ??31?0???1 ?30030??00 1100??0-10 13?021?011

?1?0?????? 11100?0000 010110111? ????111112 1001110111 1????????? ???1111?10
1?00011111 0011210111 1?1111?101 01101??111 ?0??????1? ??????????

Saltasaurus loricatus
?????????? ?????????? ??????00?? ???1010??? 010?01???? ?????????? ????010001 0??1?????1
?????????? ?????????? ?????????? 1013030002 0010100001 1000-?1111 200121-112
2112000020 1111111011 11001211?? ??311010?1 ?1?0301100 1100??0-10 1310211011
01?0??11?0 1110000000 0101101111 0???111112 1001110111 1????????? ??11111110
1100011111 0011210111 111111110? ?????????? ?????????? ??????????

Apatosaurus spp.
00201?1111 1201111?11 1100101100 ?001010001 1101010110 110010020? 01?0000100
0000000101 ?1?001???? ???2311?00 013102?331 111231?002 01-1102101 10-1131000
?110?02101 1000011001 1110010011 1-01001011 0021?11120 1100200001 1111101110
00002012?0 1111110010 11000001?0 011001?000 00?110(01)(01)12 0100101010 0111100001
1111100001 1000001?00 1011110010 0111011101 1111011111 0111111?11 11?1110001

Diplodocus spp.
0020101111 1201111111 1100101100 1001010001 1101010110 1100100201 0110000100
0010100101 1100011011 01?2311?10 0131020331 111231?002 01-0112111 10-1131000
111??02101 1000011001 1110010011 1-01011011 0021111120 1100211101 1111111112
10003012?0 1111110010 11000000?1 001001?000 0001101011 0100111010 0?????????
??11100001 1000000?00 1011100010 111101?101 1111001111 0110111111 1?111100?1

Europasaurus holgeri
11101?0?00 1111?00100 10000????? 10?1?00?01 001?01011? 1110000101 ????100000
001110?0?1 100???1111 ?111110101 1011?200?0 1012021001 1011101101 1000-?1001
1111101102 1000001000 1100110011 1010021001 0021?001?0 ?000000000 0100000010
0010200100 00--0-10?? 1100000101 0010010000 ????100011 0100111010 0??11?????
?111101?0 110000?011 0?11100010 0?11011101 01110?11?1 ?????1??1? 1??11?????

Futalognkosaurus dukei
?????????? ?????????? ?????????? ?????????? ?????????? ?????????? ?????????? ??????????
?????????? ?????????? ????????20 1??0-?0002 ?00?10?0?1 2110-31000 2??121-11? ?2120??0??
???101?01? ?-???2?1?? ??3??010?0 ?10??????? ?????????? ?????????? ?????????? 1?????????
?????????? ?????????? ?????????? ?????????? ????????1? ?000001111 00???????? ??????????
?????????? ?????????? ??????????

Dreadnoughtus schrani
?????????? ?????????? ?????????? ?????????? ?????????? ?????????? ?????????? ??????????
?????????? ?????????? 1231?202?? 1110-30102 ??0?110001 1?00-?1010 2(01)1121-111
1112001010 11(01)1111011 11000211?? 1131?????0 13?0300000 0100000-10 0310(12)00010
0???0-1110 11?0?00110 0111000111 0101101112 0111100110 1????????? ??11110110
1000011011 00112111?0 01?1111101 01100??111 ?01??????? 1?11??????

Diamantinasaurus matildae

?????????? ?????????? ?????????? ?????????? ?????????? ?????????? ?????????? ?????????? ?????????? ?????????? ?????????? ?????????? ?????????? ??????1001 211121-111 2212001000 111111?011 ??0??210?1 11???????? ?????????? ?????????? ?????????? ?????????? 1?00?0?010 0011?????? ???1101112 0101110111 1??1111001 ?1?1111110 1100011111 0011210101 0111111101 01100????? ?????????? ??????????

Notocolossus gonzalezparejasi
?????????? ?????????? ?????????? ?????????? ?????????? ?????????? ?????????? ?????????? ?????????? ?????????? ?????????? ?????????? ?????????? ???0-?1000 201120-11? 0????????? ???????0?? ?????????? ?????????? ???0300000 1000000000 0????????? ??????11?? 1????????? ?????????? ????101011 0111?????? ?????????? ?????????? ?????????? ?????????? ?????????1 0110111111 00111?1111 1001110121

XI. Supplementary Figures S1–S11

To keep the size of our Supplementary Information file to a minimum, we have included preview images of each of the three three-dimensional photogrammetric digital models of *Notocolossus gonzalezparejasi* bones (Supplementary Figures S1, S3, and S4). Readers may access and download the models themselves (as 3D .pdfs) from the data repository Figshare by following the hyperlinks provided in the caption of each figure.

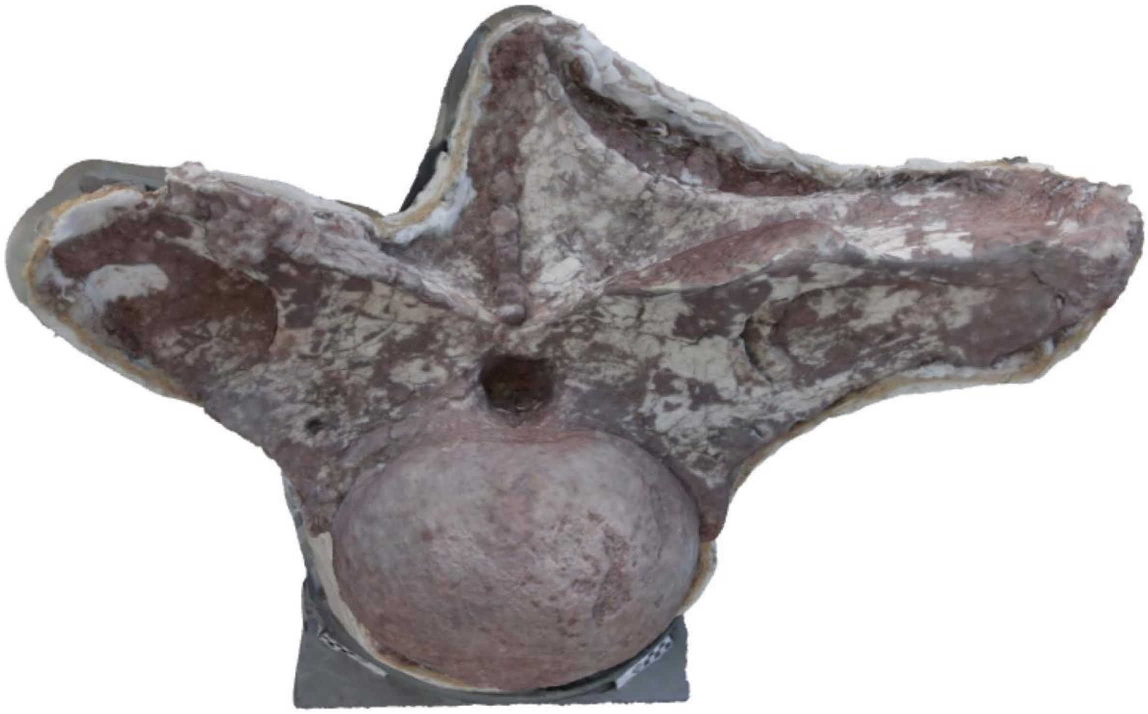

Supplementary Fig. S1. Digital photogrammetric reconstruction of the anterior dorsal vertebra of the holotype of *Notocolossus gonzalezparejasi* (UNCUYO-LD 301) in anterior view (courtesy of Stephen Poropat of the Australian Age of Dinosaurs Natural History Museum [Winton, Queensland, Australia]). Downloadable, interactive 3D .pdf file available here: http://figshare.com/s/54e3e5c87c3d11e59aec06ec4bbcf141.

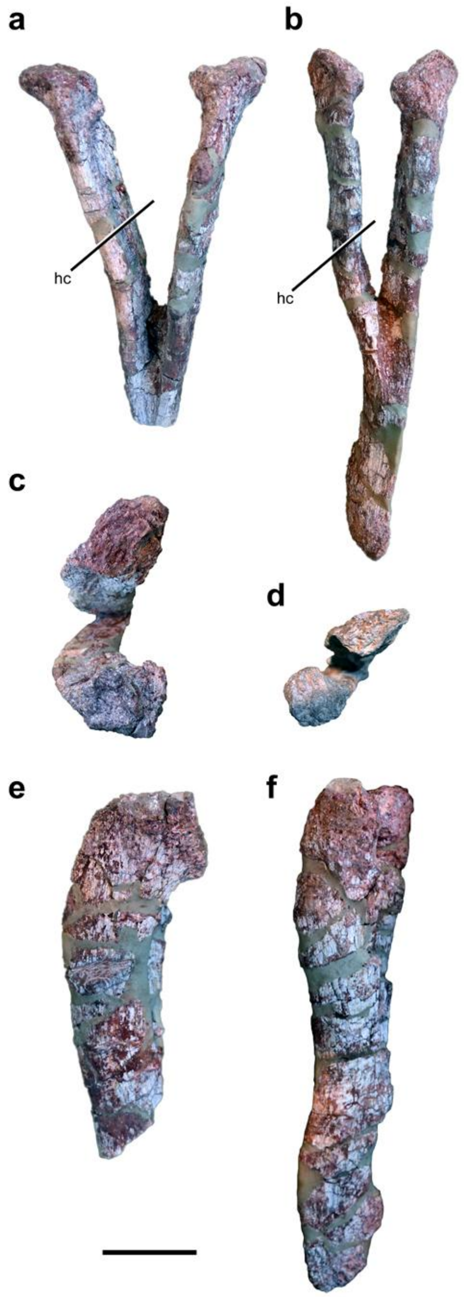

Supplementary Fig. S2. Haemal arches of the referred specimen of *Notocolossus gonzalezparejasi* (UNCUYO-LD 302). First preserved haemal arch in the sequence in (**a**) anterior, (**c**) proximal, and (**e**) left lateral views. Third preserved haemal arch in (**b**) anterior, (**d**) proximal, and (**f**) left lateral views. Abbreviation, hc, haemal canal. Scale bar, 10 cm.

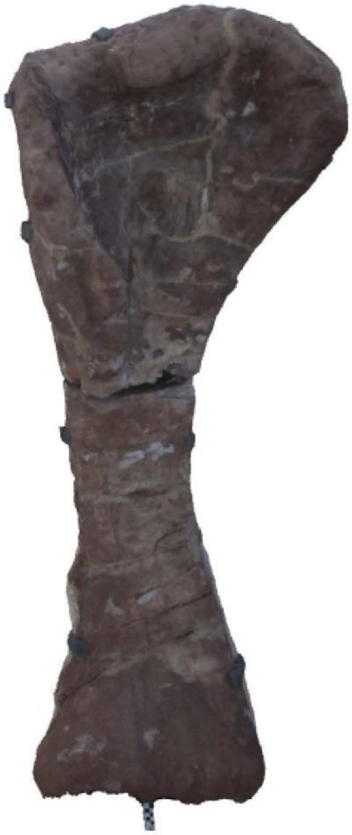

Supplementary Fig. S3. Digital photogrammetric reconstruction of the right humerus of the holotype of *Notocolossus gonzalezparejasi* (UNCUYO-LD 301) in anterior view (courtesy of Stephen Poropat of the Australian Age of Dinosaurs Natural History Museum [Winton, Queensland, Australia]). Downloadable, interactive 3D .pdf file available here: http://figshare.com/s/51de356c7c3e11e5a5db06ec4b8d1f61.

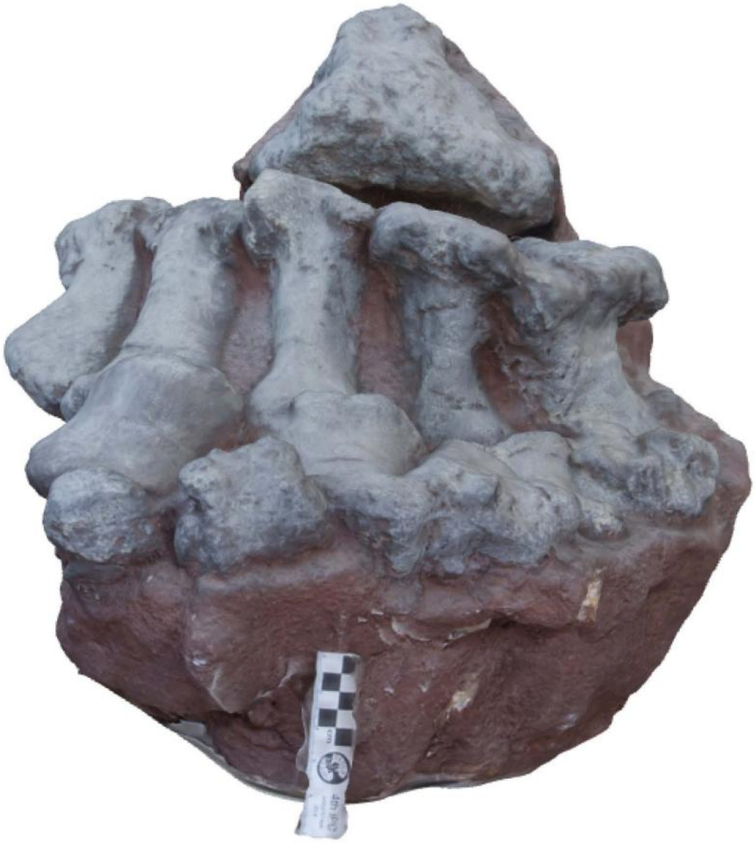

Supplementary Fig. S4. Digital photogrammetric reconstruction of a cast replica of the articulated right tarsus and pes of the referred specimen of *Notocolossus gonzalezparejasi* (UNCUYO-LD 302) in anterior view (courtesy of Stephen Poropat of the Australian Age of Dinosaurs Natural History Museum [Winton, Queensland, Australia]). The cast was made prior to the disarticulation and full preparation of these elements; as such, it documents the precise disposition of the tarsus and pes as they were discovered in the field. Downloadable, interactive 3D .pdf file available here: http://figshare.com/s/7b57cffc7c3e11e5bb5506ec4b8d1f61.

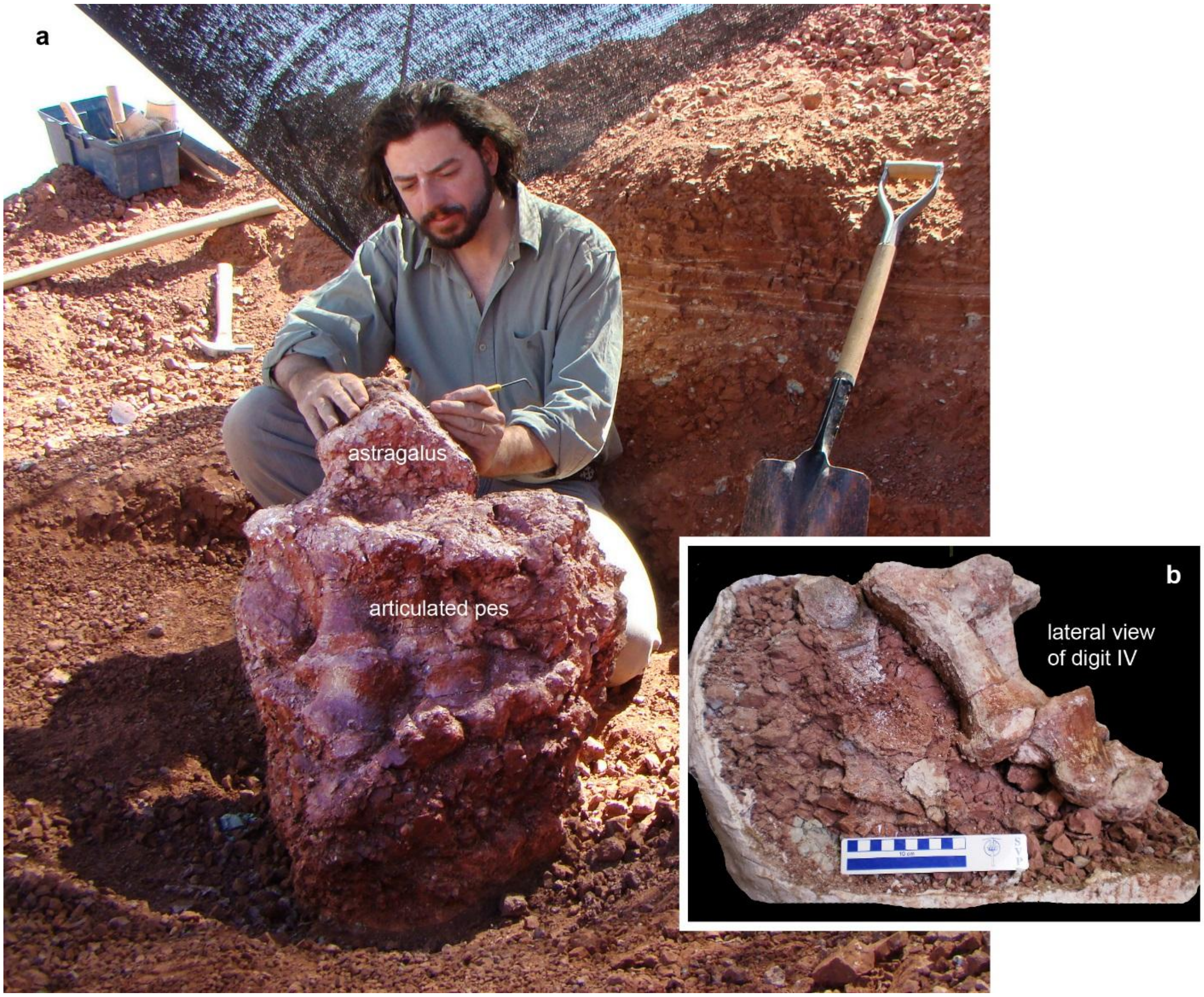

Supplementary Fig. S5. Disposition of the complete and articulated right tarsus and pes of the referred specimen of *Notocolossus gonzalezparejasi* (UNCUYO-LD 302) as they were discovered in the field. (**a**) Photograph of the tarsus and pes during excavation, with the senior author (B.J.G.R.) for scale. All elements were *in situ*, fully articulated, and preserved in approximate life position 60 cm below the modern outcrop surface. (**b**) Articulated pes in lateral view, showing the disposition of digit IV as preserved.

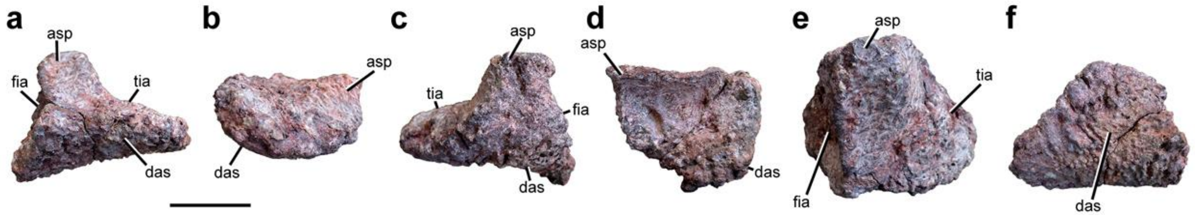

Supplementary Fig. S6. Right astragalus of the referred specimen of *Notocolossus gonzalezparejasi* (UNCUYO-LD 302) in (**a**) dorsal (= anterior), (**b**) medial, (**c**) ventral (= posterior, plantar), (**d**), lateral, (**e**) proximal, and (**f**) distal views. Abbreviations: asp, ascending process; das, distal articular surface; fia, fibular articular surface; tia, tibial articular surface. Scale bar, 10 cm.

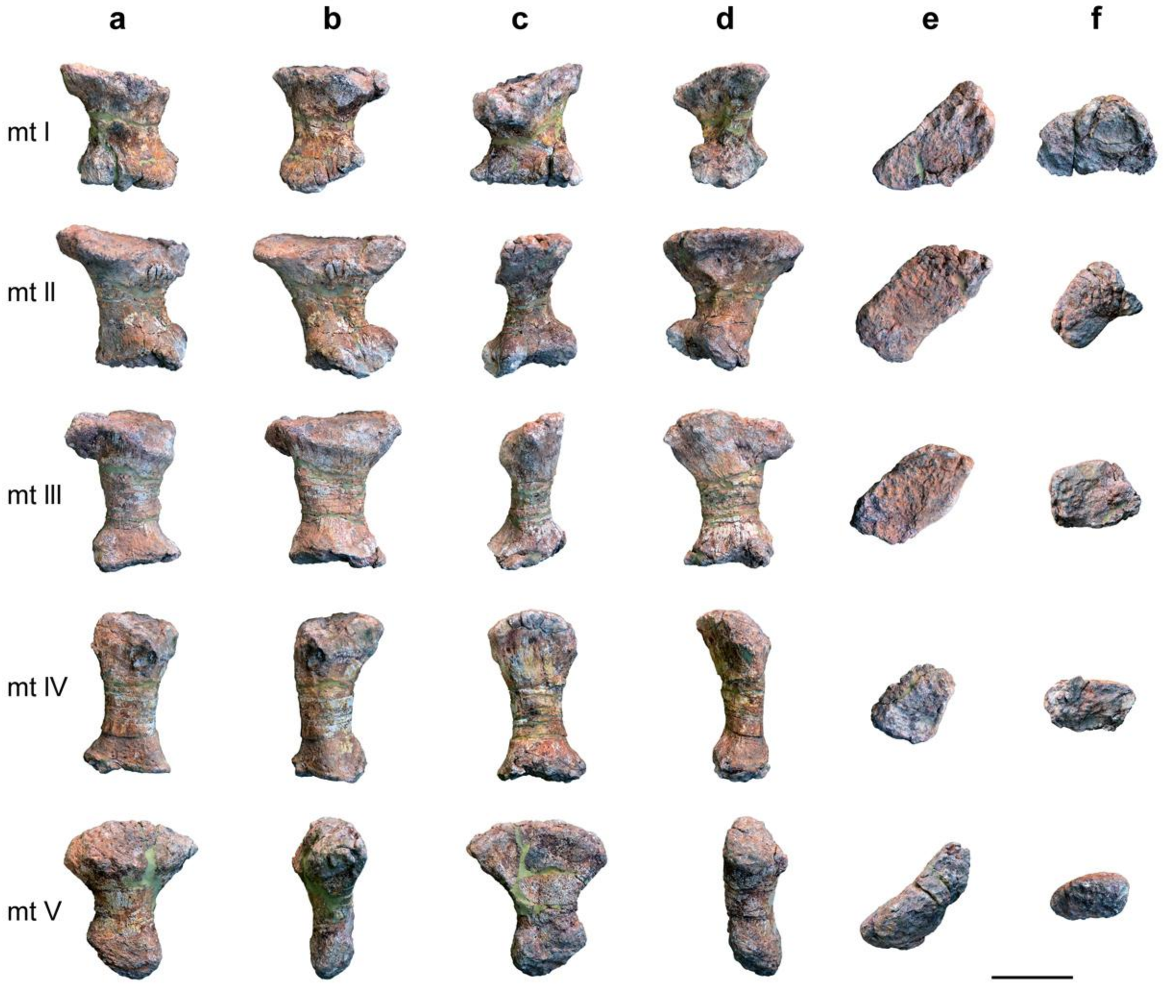

Supplementary Fig. S7. Right metatarsals of the referred specimen of *Notocolossus gonzalezparejasi* (UNCUYO-LD 302) in (**a**) dorsal (= anterior), (**b**) medial, (**c**) ventral (= posterior, plantar), (**d**) lateral, (**e**) proximal (dorsal toward bottom), and (**f**) distal (dorsal toward top) views. Abbreviations: I–V, metatarsal number; mt, metatarsal. Scale bar, 10 cm.

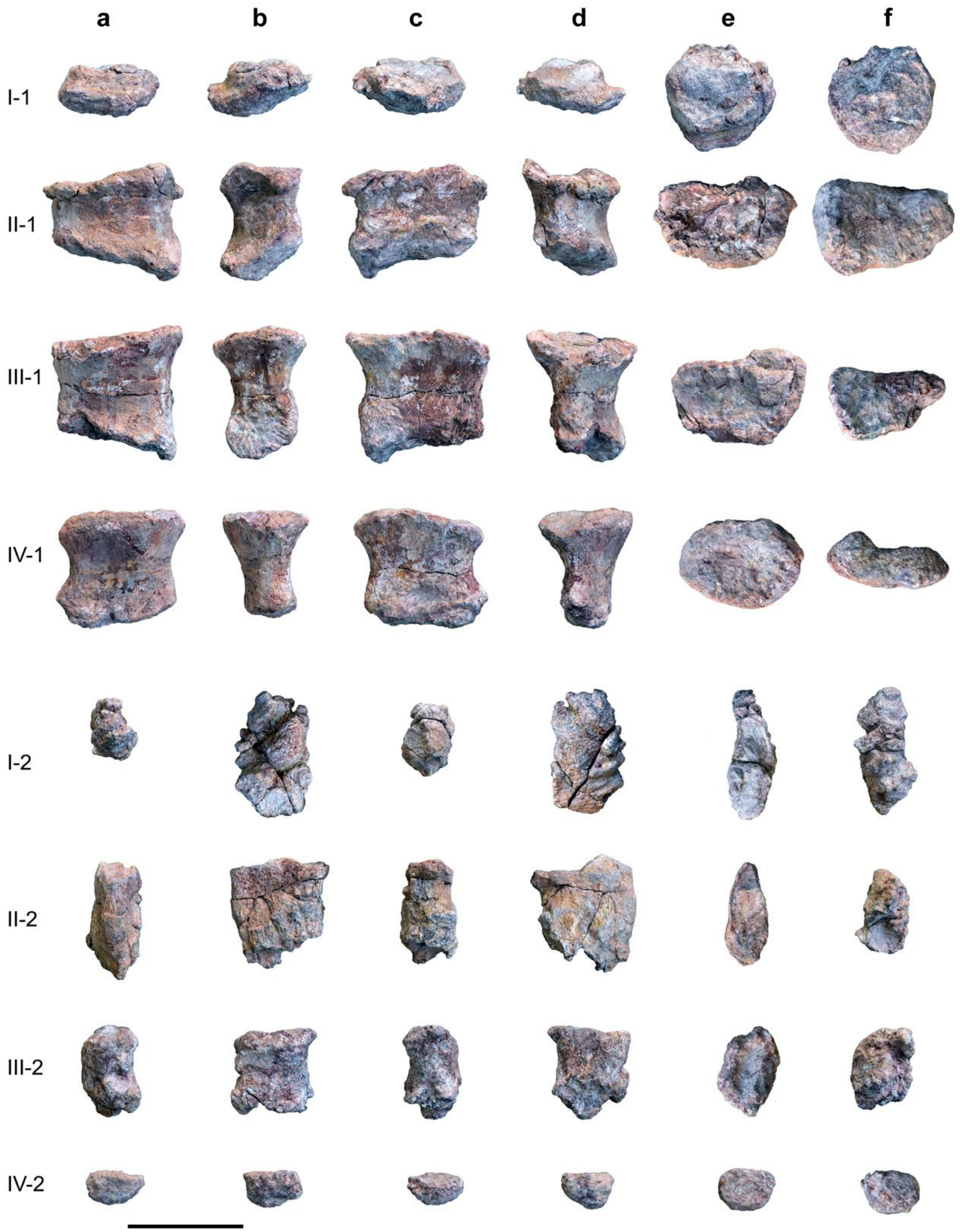

Supplementary Fig. S8. Right pedal phalanges (including unguals) of the referred specimen of *Notocolossus gonzalezparejasi* (UNCUYO-LD 302) in (**a**) dorsal (= anterior), (**b**) medial, (**c**) ventral (= posterior, plantar), (**d**) lateral, (**e**) proximal (dorsal toward bottom), and (**f**) distal

(dorsal toward top) views. Abbreviations: I–V, digit number; 1–2, phalanx number. Scale bar, 10 cm.

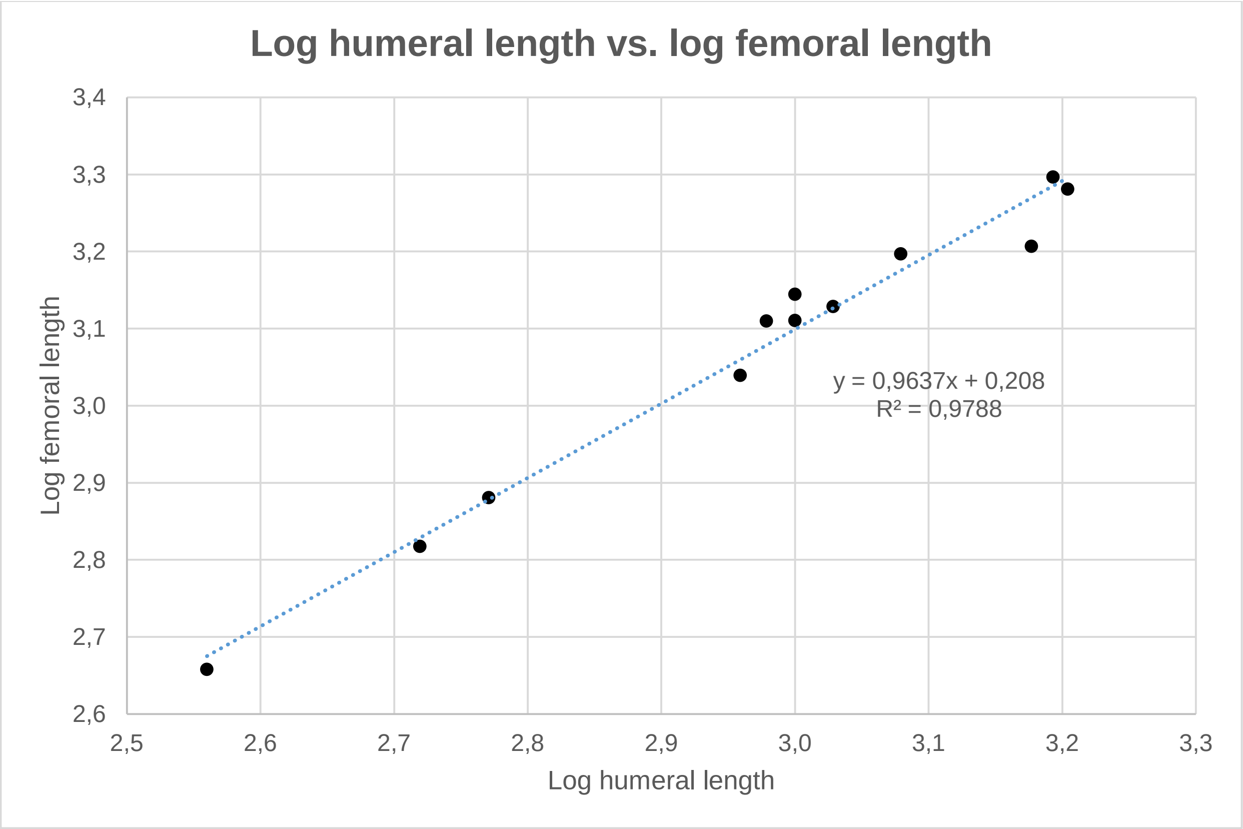

Supplementary Fig. S9. Linear regression of logarithms (base 10) of titanosaurian humeral and femoral proximodistal lengths. Associated data are presented in Supplementary Table S4. The equation generated was used to estimate the length of the unknown femur of the holotypic specimen of *Notocolossus gonzalezparejasi* (UNCUYO-LD 301) at 2166 mm.

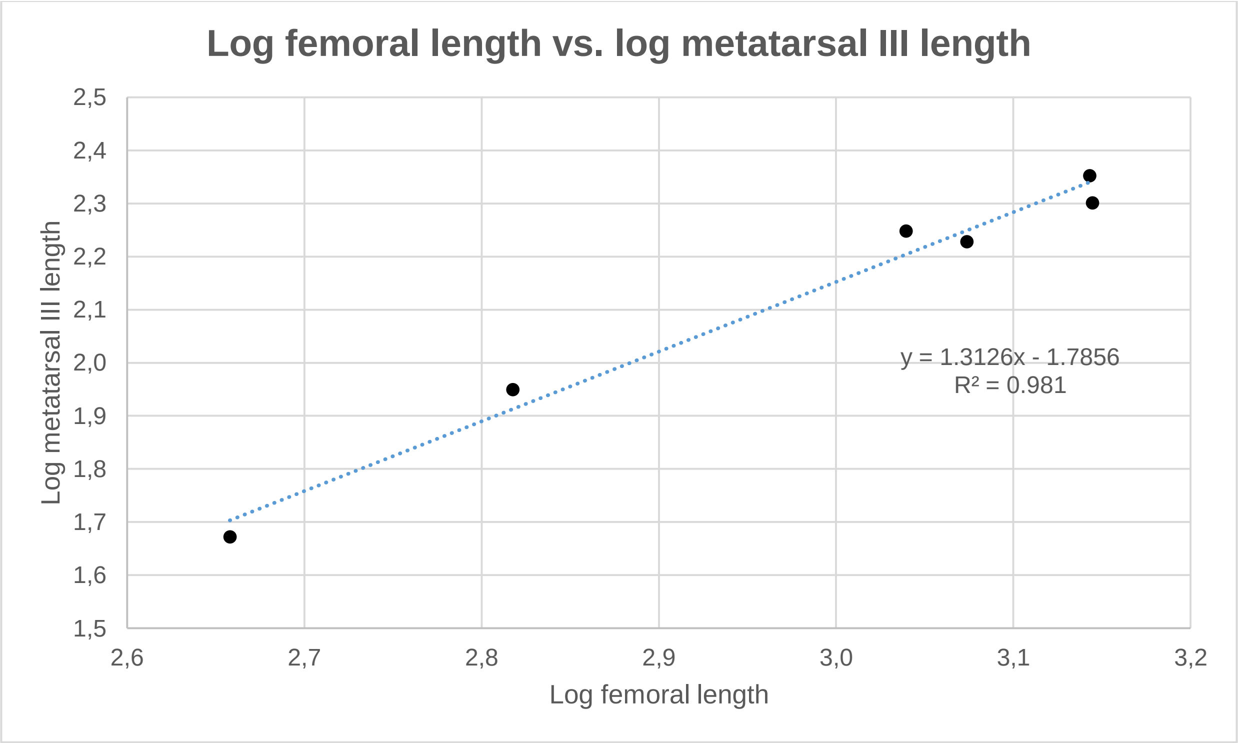

Supplementary Fig. S10. Linear regression of logarithms (base 10) of proximodistal lengths of titanosaurian femora and third metatarsals. Associated data are presented in Supplementary Table S5. The equation generated was used to estimate the length of the unknown femur of the referred specimen of *Notocolossus gonzalezparejasi* (UNCUYO-LD 302) at 1283 mm.

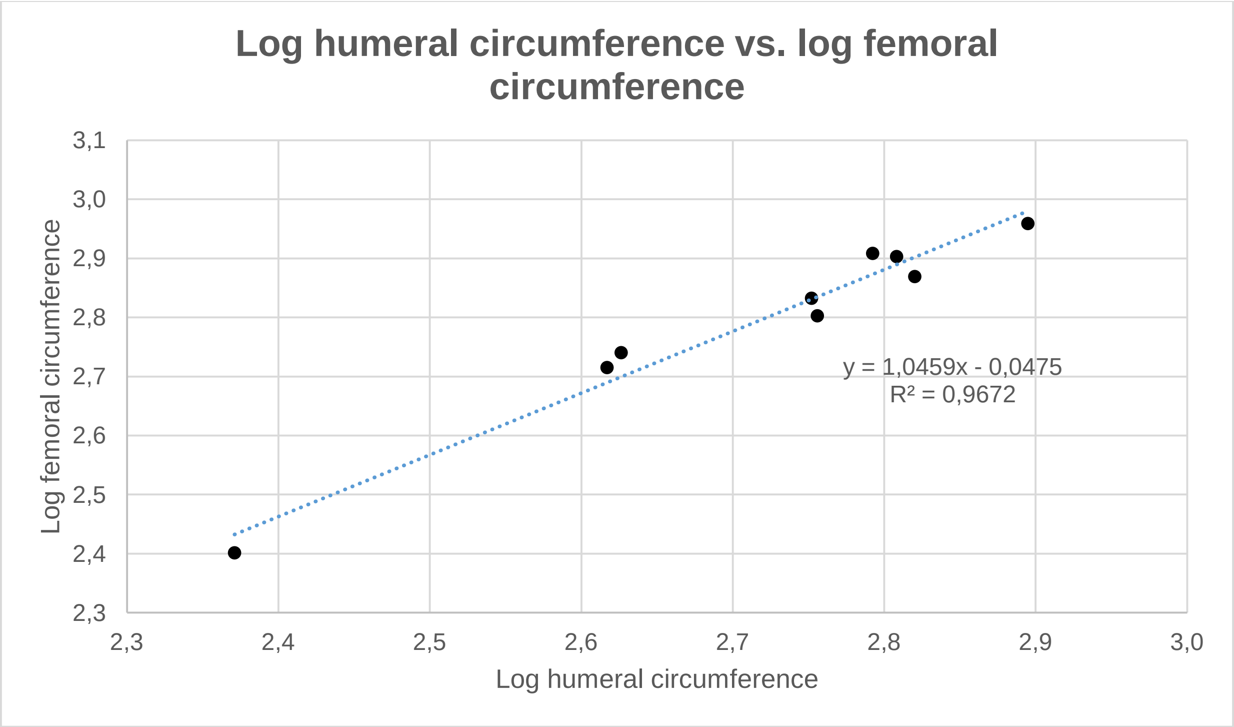

Supplementary Fig. S11. Linear regression of logarithms (base 10) of titanosaurian humeral and femoral minimum midshaft circumferences. Associated data are presented in Supplementary Table S6. The equation generated was used to estimate the circumference of the unknown femur of the holotypic specimen of *Notocolossus gonzalezparejasi* (UNCUYO-LD 301) at 936 mm.

XII. Supplementary Tables S1–S6

Supplementary Table S1. Measurements (mm) of the holotype of *Notocolossus gonzalezparejasi* (UNCUYO-LD 301). * = element incomplete, measurement as preserved; ~ = measurement estimated by doubling bilateral structure preserved on only one side of element.

**Axial Skeleton**	
**Anterior dorsal vertebra**	
Dorsoventral height, centrum, anterior	320
Transverse width, centrum, anterior	435
Dorsoventral height, total	750*
Transverse width, total	~1500
Dorsoventral height, neural canal, anterior	70
Transverse width, neural canal, anterior	91
Dorsoventral height, left parapophysis	192
Anteroposterior length, left parapophysis	73
Mediolateral width, left prezygapophysis	285
Anteroposterior length, left prezygapophysis	123
Dorsoventral height, neural spine	350*
**Anterior caudal vertebra**	
Anteroposterior length, centrum (w/ condyle)	302
Anteroposterior length, centrum (w/o condyle)	180
Dorsoventral height, centrum, anterior	260
Transverse width, centrum, anterior	291
Dorsoventral height, centrum, posterior	245
Transverse width, centrum, posterior	250
Dorsoventral height, total	550
Transverse width, total	~515
Dorsoventral height, neural spine	245
Anteroposterior length, neural spine	115
Transverse width, neural spine base	83
Transverse width, neural spine apex	149
**Appendicular Skeleton**	
**Right humerus**	
Proximodistal length	1760
Proximodistal length, deltopectoral crest	720
Mediolateral width, proximal	720
Mediolateral width, midshaft	250
Anteroposterior thickness, proximal (head)	245
Anteroposterior thickness, proximal (maximum)	385
Anteroposterior thickness, distal	147
Mediolateral width, distal	520
Circumference, midshaft	770
**Left pubis**	
Anteroposterior length, iliac peduncle	303
Mediolateral width, iliac peduncle	178

Supplementary Table S2. Measurements (mm) of the articulated anterior caudal sequence of the referred specimen of *Notocolossus gonzalezparejasi* (UNCUYO-LD 302). Numbers assigned correspond to positions in the sequence rather than positions in the complete caudal series. * = element incomplete, measurement as preserved; ** = measurement estimated; -- = element highly incomplete, measurement not provided.

**Caudal vertebrae**	**1**	**2**	**3**	**4**	**5**	**6**	**7**
Anteroposterior length, centrum (w/ condyle)	207	--	188	--	--	--	--
Anteroposterior length, centrum (w/o condyle)	157	--	143	--	--	--	--
Dorsoventral height, centrum, anterior	196**	--	145*	--	142*	--	--
Dorsoventral height, centrum, posterior	163	111*	143*	125*	123*	133*	121*
Transverse width, centrum, posterior	150	106*	106*	102*	--	125*	114*
Transverse width, total	350**	310**	266**	202**	188**	--	--

**Haemal arches**	**1**	**2**	**3**	**4**	**5**	**6**	**7**
Anteroposterior length, proximal	81	--	58	--	58	--	54
Anteroposterior length, distal	--	76	67	63	61	--	--
Proximodistal depth, total	--	--	372	--	--	--	--
Proximodistal depth, haemal canal	180	--	178	--	143	--	120

Supplementary Table S3. Measurements (mm) of the articulated right tarsus and pes of the referred specimen of *Notocolossus gonzalezparejasi* (UNCUYO-LD 302). Abbreviations: I–V, metatarsal/digit number; 1–2, phalanx number; Mt, metatarsal; Ph, phalanx.

Element	Proximodistal length	Dorsoventral depth, proximal	Mediolateral width, proximal	Dorsoventral depth, distal	Mediolateral width, distal
Astragalus	175	93	67	117	219
Mt I	164	174	94	102	144
Mt II	185	184	92	90	114
Mt III	197	170	86	91	113
Mt IV	218	114	80	71	114
Mt V	196	173	69	55	102
Ph I-1	45	71	90	55	87
Ph II-1	96	83	123	72	106
Ph III-1	115	82	115	62	105
Ph IV-1	98	76	117	42	94
Ph I-2	64	101	34	105	46
Ph II-2	102	90	37	73	41
Ph III-2	80	70	44	73	51
Ph IV-2	37	62	48	51	40

Supplementary Table S4. Proximodistal lengths (mm) of the humerus and femur in articulated or definitively associated titanosaurian specimens. Measurements for *Bonatitan reigi* are from 'Individual D' of Salgado *et al.*[63]. The left femur of '*Antarctosaurus*' *giganteus* (MLP 26-316) is 2310 mm in length[20]; however, the average length of the two femora of the specimen is 2265 mm, as reported below. Specimens are listed by increasing femoral length. All specimens that preserve both the humerus and femur in their entirety (i.e., MACN-PV RN 821/RN 1061 ['Individual D'] through MUCPv-323) were used to generate the regression equation presented in Supplementary Fig. S9; for the remaining specimens (MMCH NR through MLP 26-316), humeral or femoral length was estimated using this equation. The estimated femoral length of the holotypic specimen of *Notocolossus gonzalezparejasi* (UNCUYO-LD 301) is 2166 mm. Abbreviations: L_F, femoral length; L_H, humeral length; log, logarithm (base 10); NR, not reported. Institutional abbreviations see section I above. a = measurement averaged from left and right elements of specimen in question; ** = measurement estimated.

Taxon	Specimen(s)	Source(s)	L_H	L_F	log L_H	log L_F
Bonatitan reigi	MACN-PV RN 821/RN 1061	63, 64	363	455	2.55991	2.65801
Rapetosaurus krausei	FMNH PR 2209	65	524	657	2.71933	2.81757
Quebrada La Higuera indet.	NR	66	590	760	2.77085	2.88081
Epachthosaurus sciuttoi	UNPSJB-PV 920	31	910a	1095	2.95904	3.03941
Jainosaurus cf. *septentrionalis*	NHMUK R5931/R5903	67	952	1288	2.97864	3.10992
Aegyptosaurus baharijensis	BSPG 1912 VIII 61	68	1000	1290	3.00000	3.11059
Diamantinasaurus matildae	AODF 603	58	1068	1345	3.02857	3.12872
Opisthocoelicaudia skarzynskii	ZPAL MgD-I/48	69	1000	1395	3.00000	3.14457
Petrobrasaurus puestohernandezi	MAU-Pv-PH-449	23	1200	1574a	3.07918	3.19700
Alamosaurus sanjuanensis	TMM 41541-1	70, Lehman pers. comm.	1503	1610	3.17696	3.20683
Dreadnoughtus schrani	MPM-PV 1156	28	1600	1910	3.20412	3.28103
Futalognkosaurus dukei	MUCPv-323	36	1560	1980	3.19312	3.29667
Huincul indet.	MMCH NR	71	1660	2047**	3.22011	3.31122**
Paralititan stromeri	CGM 81119	34	1690	2083**	3.22789	3.31871**
Notocolossus gonzalezparejasi	UNCUYO-LD 301	This paper	1760	2166**	3.24551	3.33570**
Argentinosaurus huinculensis?	MCF-PVPH NR	72	1831**	2250	3.26262**	3.35218
'*Antarctosaurus*' *giganteus*	MLP 26-316	20	1843**	2265a	3.26561**	3.35507

Supplementary Table S5. Proximodistal lengths (mm) of the femur and metatarsal III in articulated or definitively associated titanosaurian specimens. Measurements for *Bonatitan reigi* are from 'Individual D' of Salgado *et al.*[63]. Specimens are listed by increasing femoral length. All specimens that preserve both the femur and metatarsal III in their entirety (i.e., MACN-PV RN 1061 ['Individual D'] through ZPAL MgD-I/48) were used to generate the regression equation presented in Supplementary Fig. S10; this equation was then used to estimate the femoral lengths of the referred specimen of *Notocolossus gonzalezparejasi* (UNCUYO-LD 302) and ?*Alamosaurus sanjuanensis* specimen NMMNH P-49967 at 1283 mm and 1632 mm, respectively. Abbreviations: L_F, femoral length; L_{MTIII}, metatarsal III length; log, logarithm (base 10). Institutional abbreviations see section I above. ** = measurement estimated.

Taxon	Specimen(s)	Source(s)	L_F	L_{MTIII}	log L_F	log L_{MTIII}
Bonatitan reigi	MACN-PV RN 1061	63, 64	455	47	2.65801	1.67210
Rapetosaurus krausei	FMNH PR 2209	65	657	89	2.81757	1.94939
Epachthosaurus sciuttoi	UNPSJB-PV 920	31	1095	177	3.03941	2.24797
Bonitasaura salgadoi	MPCA 460	73	1185	169	3.07372	2.22789
Antarctosaurus wichmannianus	MACN-PV 6904	20	1390	225	3.14301	2.35218
Opisthocoelicaudia skarzynskii	ZPAL MgD-I/48	69	1395	200	3.14457	2.30103
Notocolossus gonzalezparejasi	UNCUYO-LD 302	This paper	1283**	197	3.10839**	2.29447
?*Alamosaurus sanjuanensis*	NMMNH P-49967	37	1632**	270	3.21268**	2.43136

Supplementary Table S6. Minimum midshaft circumferences (mm) of the humerus and femur and estimated body mass (kg) in articulated or definitively associated titanosaurian specimens. All specimens that preserve both the humeral and femoral circumference (i.e., FMNH PR 2209 through MPM-PV 1156) were used to generate the regression equation presented in Supplementary Fig. S11; for the remaining specimens (CGM 81119 through MCF-PVPH NR), humeral or femoral circumference was estimated using this equation. The estimated femoral circumference of the holotypic specimen of *Notocolossus gonzalezparejasi* (UNCUYO-LD 301) is 936 mm. Actual or actual/estimated humeral and femoral circumferences were then used to estimate the body mass of each specimen using a scaling equation presented by Campione and Evans[38] (equation 1 of those authors; $\log BM = 2.749 * \log C_{H+F} – 1.104$, where BM is body mass and C_{H+F} is combined humeral and femoral circumference). Abbreviations: BM, body mass; C_F, femoral circumference; C_H, humeral circumference; C_{H+F}, combined humeral and femoral circumference; log, logarithm (base 10); NR, not reported. Institutional abbreviations see section I above. ** = measurement estimated.

Taxon	Specimen(s)	Source(s)	C_H	C_F	log C_H	log C_F	C_{H+F}	log C_{H+F}	BM
Rapetosaurus krausei	FMNH PR 2209	Curry Rogers pers. comm.	235	252	2.37107	2.40140	487	2.68753	1923**
Jainosaurus cf. *septentrionalis*	NHMUK R5931/R5903	McIntosh pers. comm.	414	519	2.61700	2.71517	933	2.96988	11487**
Epachthosaurus sciuttoi	UNPSJB-PV 920	31	423	550	2.62634	2.74036	973	2.98811	12892**
Diamantinasaurus matildae	AODF 603	58, 72	570	635	2.75587	2.80277	1205	3.08099	23208**
Opisthocoelicaudia skarzynskii	ZPAL MgD-I/48	74	565	680	2.75205	2.83251	1245	3.09517	25388**
Alamosaurus sanjuanensis	TMM 41541-1	70	661	740	2.82020	2.86923	1401	3.14644	35120**
Elaltitan lilloi	PVL 4628	22	620	810	2.79239	2.90849	1430	3.15534	37155**
Futalognkosaurus dukei	MUCPv-323	72	643	800	2.80821	2.90309	1443	3.15927	38091**
Dreadnoughtus schrani	MPM-PV 1156	28	785	910	2.89487	2.95904	1695	3.22917	59291**
Paralititan stromeri	CGM 81119	Lamanna unpublished data	720	873**	2.85733	2.94098**	1593**	3.20220**	49986**
Notocolossus gonzalezparejasi	UNCUYO-LD 301	This paper	770	936**	2.88649	2.97148**	1706**	3.23209**	60398**
'*Antarctosaurus*' *giganteus*	MLP 26-316	74	662**	800	2.82110**	2.90309	1462**	3.16506**	39513**
Argentinosaurus huinculensis?	MCF-PVPH NR	72	909**	1114	2.95859**	3.04689	2023**	3.30601**	96430**

XIII. References for Supplementary Information

1 González Riga, B. J. A new titanosaur (Dinosauria, Sauropoda) from the Upper Cretaceous of Mendoza Province, Argentina. *Ameghiniana* **40**, 155–172 (2003).

2 González Riga, B. J. Nuevos restos fósiles de *Mendozasaurus neguyelap* (Sauropoda, Titanosauria) del Cretácico tardío de Mendoza, Argentina. *Ameghiniana* **42**, 535–548 (2005).

3 González Riga, B. J. & Astini, R. A. Preservation of large titanosaur sauropods in overbank fluvial facies: a case study in the Cretaceous of Argentina. *J. S. Am. Earth Sci.* **23**, 290–303 (2007).

4 González Riga, B. J. & Ortiz David, L. A new titanosaur (Dinosauria, Sauropoda) from the Upper Cretaceous (Cerro Lisandro Formation) of Mendoza Province, Argentina. *Ameghiniana* **51**, 3–25 (2014).

5 González Riga, B. J., Previtera, E. & Pirrone, C. A. *Malarguesaurus florenciae* gen. et sp. nov., a new titanosauriform (Dinosauria, Sauropoda) from the Upper Cretaceous of Mendoza, Argentina. *Cretaceous Res.* **30**, 135–148 (2009).

6 Sereno, P. C. *et al.* Evidence for avian intrathoracic air sacs in a new predatory dinosaur from Argentina. *PLoS ONE* **3**, e3303 (2008).

7 González Riga, B. J. *et al.* Sauropod and theropod dinosaur tracks from the Upper Cretaceous of Mendoza (Argentina): trackmakers and anatomical evidences. *J. S. Am. Earth Sci.* **61**, 134–141 (2015).

8 González Riga, B. J., Monardez, C., Ortiz David, L. D. & Redonte, G. in *Reunión de comunicaciones de la Asociación Paleontologica Argentina (Roca) actas* 57 (2012).

9 González Riga, B. J. *et al.* in *Abstract book, 1st Brazilian Dinosaur Symposium* (eds Candeiro, C. R. A., Machado, E. B. & Alves, Y. M.) 35 (Universidade Federal de Uberlândia, Campus Pontal-Ituiutaba, 2013).

10 González Riga, B. J. & Lamanna, M. C. in *4th International Palaeontological Congress, The History of Life: A View from the Southern Hemisphere abstract volume* (ed Cerdeño, E.) 316 (International Palaeontological Association, 2014).

11 Digregorio, J. H. & Uliana, M. A. in *Segundo Simposio de Geología Regional Argentina* (ed García Castellanos, T.) 985–1032 (Academia Nacional de Ciencias, 1980).

12 Legarreta, L. & Uliana, M. A. in *Sedimentation, tectonics, and eustasy, IAS Special Publication* **12**, 429–450 (International Association of Sedimentology, 1991).

13 Leanza, H. A., Apesteguía, S., Novas, F. E. & de la Fuente, M. S. Cretaceous terrestrial beds from the Neuquén Basin (Argentina) and their tetrapod assemblages. *Cretaceous Res.* **25**, 61–87 (2004).

14 de la Fuente, M. S. *et al.* Tetrápodos continentales del Cretácico de la Argentina: una síntesis actualizada. *Asoc. Paleontol. Argentina, Publ. Esp.* **11**, 137–153 (2007).

15 González Riga, B. J. in *Paleontología y dinosaurios desde América Latina* (eds Calvo, J., Porfiri, J., González Riga, B. J. & Dos Santos. D.) 125–141 (Universidad Nacional de Cuyo, 2011).

16 Calvo, J. O. *et al.* in *18° Congreso Geológico Argentino (Neuquén), relatorio.* 539–556.

17 Leanza, H. A. & Hugo, C. A. Cretaceous red beds from southern Neuquén Basin (Argentina): age, distribution and stratigraphic discontinuities. *Asoc. Paleontol. Argentina, Publ. Esp.* **7**, 117–122 (2001).

18 Ramos, V. A. *Descripción geológica de la hoja 33 c, Los Chihuidos Norte, provincia del Neuquén: carta geológico-económica de la República Argentina, escala 1: 200.000*. (Servicio Geológico Nacional, 1981).
19 Garrido, A. C. Estratigrafía del Grupo Neuquén, Cretácico Superior de la Cuenca Neuquina (Argentina): nueva propuesta de ordenamiento litoestratigráfico. *Rev. Mus. Argent. Cienc. Nat., n.s.* **12**, 121–177 (2010).
20 Huene, F. von. Los saurisquios y ornitisquios del Cretáceo Argentino. *An. Mus. La Plata* **3**, 1–196 (1929).
21 Bonaparte, J. F. & Gasparini, Z. B. Los saurópodos de los grupos Neuquén y Chubut, y sus relaciones cronologicas. *Actas VII Congr. Geol. Argentino, Neuquén* **2**, 393–406 (1979).
22 Powell, J. E. Revision of South American titanosaurid dinosaurs: palaeobiological, palaeobiogeographical and phylogenetic aspects. *Rec. Queen Victoria Mus.* **111**, 1–173 (2003).
23 Filippi, L. S. *et al.* A new sauropod titanosaur from the Plottier Formation (Upper Cretaceous) of Patagonia (Argentina). *Geol. Acta* **9**, 1–12 (2011).
24 Calvo, J. O., González Riga, B. J. & Porfiri, J. D. A new titanosaur sauropod from the Late Cretaceous of Neuquén, Patagonia, Argentina. *Arq. Mus. Nac., Rio de Janeiro* **65**, 485–504 (2007).
25 Filippi, L. S., Martinelli, A. G. & Garrido, A. C. Registro de un dinosaurio Aeolosaurini (Sauropoda, Titanosauria) en el Cretácico Superior (Formación Plottier) del norte de la Provincia de Neuquén, Argentina, y comentarios sobre los Aeolosaurini sudamericanos. *Rev. Brasileira Paleontol.* **16**, 147–156 (2013).
26 Upchurch, P., Barrett, P. M. & Dodson, P. Sauropoda in *The Dinosauria 2nd edn* (eds Weishampel, D. B., Dodson, P. & Osmólska, H.) Ch. 13, 259–322 (University of California Press, 2004).
27 Paul, G. S. The brachiosaur giants of the Morrison and Tendaguru with a description of a new subgenus, *Giraffatitan,* and a comparison of the world's largest dinosaurs. *Hunteria* **2**, 1–14 (1988).
28 Lacovara, K. J. *et al.* A gigantic, exceptionally complete titanosaurian sauropod dinosaur from southern Patagonia, Argentina. *Sci. Rep.* **4**, 6196 (2014).
29 Gilmore, C. W. Reptilian fauna of the North Horn Formation of central Utah. *US Dept. Interior Prof. Pap.* **210-C**, 1–53 (1946).
30 Kellner, A. W. A., Campos, D. de A. & Trotta, M. N. F. Description of a titanosaurid caudal series from the Bauru Group, Late Cretaceous of Brazil. *Arq. Mus. Nac., Rio de Janeiro* **63**, 529–564 (2005).
31 Martínez, R. D., Giménez, O., Rodríguez, J., Luna, M. & Lamanna, M. C. An articulated specimen of the basal titanosaurian (Dinosauria: Sauropoda) *Epachthosaurus sciuttoi* from the early Late Cretaceous Bajo Barreal Formation of Chubut Province, Argentina. *J. Vertebr. Paleontol.* **24**, 107–120 (2004).
32 Wilson, J. A., D'Emic, M. D., Ikejiri, T., Moacdieh, E. M. & Whitlock, J. A. A nomenclature for vertebral fossae in sauropods and other saurischian dinosaurs. *PLoS ONE* **6**, e17114 (2011).
33 Calvo, J. O., Porfiri, J. D., González Riga, B. J. & Kellner, A. W. A. Anatomy of *Futalognkosaurus dukei* Calvo, Porfiri, González Riga & Kellner, 2007 (Dinosauria,

Titanosauridae) from the Neuquén Group (Late Cretaceous), Patagonia, Argentina. *Arq. Mus. Nac., Rio de Janeiro* **65**, 511–526 (2007).
34 Smith, J. B. *et al.* A giant sauropod dinosaur from an Upper Cretaceous mangrove deposit in Egypt. *Science* **292**, 1704–1706 (2001).
35 Lamanna, M. C. *Late Cretaceous dinosaurs and crocodyliforms from Egypt and Argentina*. Unpublished doctoral dissertation, University of Pennsylvania, Philadelphia, 305 p. (2004).
36 Calvo, J. O. New fossil remains of *Futalognkosaurus dukei* (Sauropoda, Titanosauria) from the Late Cretaceous of Neuquén, Argentina in *4th International Palaeontological Congress, The History of Life: A View from the Southern Hemisphere abstract volume* (ed Cerdeño, E.) 325 (International Palaeontological Association, 2014).
37 D'Emic, M. D., Wilson, J. A. & Williamson, T. E. A sauropod dinosaur pes from the latest Cretaceous of North America and the validity of *Alamosaurus sanjuanensis* (Sauropoda, Titanosauria). *J. Vertebr. Paleontol.* **31**, 1072–1079 (2011).
38 Campione, N. E. & D. C. Evans. A universal scaling relationship between body mass and proximal limb bone dimensions in quadrupedal terrestrial tetrapods. *BMC Biology* **10**, 60 (2012).
39 Bates, K. T., Falkingham, P. L., Macaulay, S., Brassey, C. & Maidment, S. C. R. Downsizing a giant: re-evaluating *Dreadnoughtus* body mass. *Biol. Lett.* **11** (2015).
40 Carballido, J. L. & Sander, P. M. Postcranial axial skeleton of *Europasaurus holgeri* (Dinosauria, Sauropoda) from the Upper Jurassic of Germany: implications for sauropod ontogeny and phylogenetic relationships of basal Macronaria. *J. Syst. Palaeontol.* **12**, 335–387 (2014).
41 Mannion, P. D., Upchurch, P., Barnes, R. N. & Mateus, O. Osteology of the Late Jurassic Portuguese sauropod dinosaur *Lusotitan atalaiensis* (Macronaria) and the evolutionary history of basal titanosauriforms. *Zool. J. Linn. Soc.* **168**, 98–206 (2013).
42 Curry Rogers, K. Titanosauria: a phylogenetic overview in *The sauropods: evolution and paleobiology* (eds Curry Rogers, K. & Wilson, J. A.) Ch. 2, 50–103 (University of California Press, 2005).
43 Upchurch, P. The phylogenetic relationships of sauropod dinosaurs. *Zool. J. Linn. Soc.* **124**, 43–103 (1998).
44 Wilson, J. A. Sauropod dinosaur phylogeny: critique and cladistic analysis. *Zool. J. Linn. Soc.* **136**, 217–276 (2002).
45 Whitlock, J. A. A phylogenetic analysis of Diplodocoidea (Saurischia: Sauropoda). *Zool. J. Linn. Soc.* **161**, 872–915 (2011).
46 Zaher, H. *et al.* A complete skull of an Early Cretaceous sauropod and the evolution of advanced titanosaurians. *PLoS ONE* **6**, e16663 (2011).
47 Carballido, J. L., Salgado, L., Pol, D., Canudo, J. I. & Garrido, A. A new basal rebbachisaurid (Sauropoda, Diplodocoidea) from the Early Cretaceous of the Neuquén Basin; evolution and biogeography of the group. *Hist. Biol.* **24**, 631–654 (2012).
48 Mannion, P. D., Upchurch, P., Mateus, O., Barnes, R. N. & Jones, M. E. H. New information on the anatomy and systematic position of *Dinheirosaurus lourinhanensis* (Sauropoda: Diplodocoidea) from the Late Jurassic of Portugal, with a review of European diplodocoids. *J. Syst. Palaeontol.* **10**, 521–551 (2012).
49 Calvo, J. O. Jaw mechanics in sauropod dinosaurs. *Gaia* **10**, 183–193 (1994).

50 Salgado, L., Coria, R. A. & Calvo, J. O. Evolution of titanosaurid sauropods. I: phylogenetic analysis based on the postcranial evidence. *Ameghiniana* **34**, 3–32 (1997).
51 Harris, J. D. The significance of *Suuwassea emilieae* (Dinosauria: Sauropoda) for flagellicaudatan intrarelationships and evolution. *J. Syst. Palaeontol.* **4**, 185–198 (2006).
52 Sereno, P. C., Wilson, J. A., Witmer, L. M., Whitlock, J. A. & Maga, A. Structural extremes in a Cretaceous dinosaur. *PLoS ONE* **2**, e1230 (2007).
53 Carballido, J. L., Pol, D., Cerda, I. & Salgado, L. The osteology of *Chubutisaurus insignis* del Corro, 1975 (Dinosauria: Neosauropoda) from the 'middle' Cretaceous of central Patagonia, Argentina. *J. Vertebr. Paleontol.* **31**, 93–110 (2011).
54 Upchurch, P., Barrett, P. M. & Galton, P. M. A phylogenetic analysis of basal sauropodomorph relationships: implications for the origin of sauropod dinosaurs. *Spec. Pap. Palaeontol.* **77**, 57–90 (2007).
55 Mannion, P. D. A rebbachisaurid sauropod from the Lower Cretaceous of the Isle of Wight, England. *Cretaceous Res.* **30**, 521–526 (2009).
56 Wilson, J. A. & Upchurch, P. A revision of *Titanosaurus* Lydekker (Dinosauria – Sauropoda), the first dinosaur genus with a 'Gondwanan' distribution. *J. Syst. Palaeontol.* **1**, 125–160 (2003).
57 Allain, R. & Aquesbi, N. Anatomy and phylogenetic relationships of *Tazoudasaurus naimi* (Dinosauria, Sauropoda) from the late Early Jurassic of Morocco. *Geodiversitas* **30**, 345–424 (2008).
58 Poropat, S. F. *et al.* Revision of the sauropod dinosaur *Diamantinasaurus matildae* Hocknull *et al.* 2009 from the mid-Cretaceous of Australia: implications for Gondwanan titanosauriform dispersal. *Gondwana Res.* **27**, 995–1033 (2015).
59 Tidwell, V., Carpenter, K. & Brooks, W. New sauropod from the Lower Cretaceous of Utah, USA. *Oryctos* **2**, 21–37 (1999).
60 Gallup, M. R. Functional morphology of the hindfoot of the Texas sauropod *Pleurocoelus* sp. indet. *Geol. Soc. Am. Spec. Pap.* **238**, 71–74 (1989).
61 D'Emic, M. D. Revision of the sauropod dinosaurs of the Lower Cretaceous Trinity Group, southern USA, with the description of a new genus. *J. Syst. Palaeontol.* **11**, 707–726 (2013).
62 Bonaparte, J. F., González Riga, B. J. & Apesteguía, S. *Ligabuesaurus leanzai* gen. et sp. nov. (Dinosauria, Sauropoda), a new titanosaur from the Lohan Cura Formation (Aptian, Lower Cretaceous) of Neuquén, Patagonia, Argentina. *Cretaceous Res.* **27**, 364–376 (2006).
63 Salgado, L., Gallina, P. A. & Paulina Carabajal, A. Redescription of *Bonatitan reigi* (Sauropoda: Titanosauria), from the Campanian–Maastrichtian of the Río Negro Province (Argentina). *Hist. Biol.* **27**, 525–548 (2015).
64 Martinelli, A. G. & Forasiepi, A. Late Cretaceous vertebrates from Bajo de Santa Rosa (Allen Formation), Río Negro Province, Argentina, with the description of a new sauropod dinosaur (Titanosauridae). *Rev. Mus. Argent. Cienc. Nat., n.s.* **6**, 257–305 (2004).
65 Curry Rogers, K. The postcranial osteology of *Rapetosaurus krausei* (Sauropoda: Titanosauria) from the Late Cretaceous of Madagascar. *J. Vertebr. Paleontol.* **29**, 1046–1086 (2009).
66 Iriarte, J., Moreno, K., Rubilar, D. & Vargas, A. A titanosaurid from the Quebrada La Higuera Formation (Upper Cretaceous), III Región, Chile. *Ameghiniana* **36**, 102 (1999).

67 Wilson, J. A., Barrett, P. M. & Carrano, M. T. An associated partial skeleton of *Jainosaurus* cf. *septentrionalis* (Dinosauria: Sauropoda) from the Late Cretaceous of Chhota Simla, Central India. *Palaeontol.* **54**, 981–998 (2011).
68 Stromer, E. Ergebnisse der Forschungreisen Prof. E. Stromers in den Wüsten Ägyptens II. Wirbeltierreste der Baharîje-Stufe (unterstes Cenoman) II. Sauropoda. *Abh. Bayerischen Akad. Wissensch. N. F.* **10**, 3–21 (1932).
69 Borsuk-Bialynicka, M. A new camarasaurid sauropod *Opisthocoelicaudia skarzynskii* gen. n., sp. n. from the Upper Cretaceous of Mongolia. *Palaeontol. Pol.* **37**, 5–63 (1977).
70 Lehman, T. M. & Coulson, A. B. A juvenile specimen of the sauropod dinosaur *Alamosaurus sanjuanensis* from the Upper Cretaceous of Big Bend National Park, Texas. *J. Paleontol.* **76**, 156–172 (2002).
71 Simón, M. E. A giant sauropod from the Upper Cretaceous of El Chocón, Neuquén, Argentina. *Ameghiniana* **38**, 19R (2001).
72 Benson, R. B. *et al.* Rates of dinosaur body mass evolution indicate 170 million years of sustained ecological innovation on the avian stem lineage. *PLoS Biol.* **12**, e1001853 (2014).
73 Gallina, P. A. & Apesteguía, S. Postcranial anatomy of *Bonitasaura salgadoi* (Sauropoda, Titanosauria) from the Late Cretaceous of Patagonia. *J. Vertebr. Paleontol.* **35**, e924957 (2015).
74 Anderson, J. F., Hall-Martin, A. & Russell, D. A. Long-bone circumference and weight in mammals, birds and dinosaurs. *J. Zool., London (A)* **207**, 53–61 (1985).
